# A Molecular Host Response Assay to Discriminate Between Sepsis and Infection-Negative Systemic Inflammation in Critically Ill Patients: Discovery and Validation in Independent Cohorts

**DOI:** 10.1371/journal.pmed.1001916

**Published:** 2015-12-08

**Authors:** Leo McHugh, Therese A. Seldon, Roslyn A. Brandon, James T. Kirk, Antony Rapisarda, Allison J. Sutherland, Jeffrey J. Presneill, Deon J. Venter, Jeffrey Lipman, Mervyn R. Thomas, Peter M. C. Klein Klouwenberg, Lonneke van Vught, Brendon Scicluna, Marc Bonten, Olaf L. Cremer, Marcus J. Schultz, Tom van der Poll, Thomas D. Yager, Richard B. Brandon

**Affiliations:** 1 Immunexpress, Seattle, Washington, United States of America; 2 Biosurgical Ingenuity, Paddington, Queensland, Australia; 3 Department of Intensive Care Medicine, Mater Health Services, South Brisbane, Queensland, Australia; 4 Department of Pathology, Mater Health Services, South Brisbane, Queensland, Australia; 5 School of Medicine, University of Queensland, St. Lucia, Queensland, Australia; 6 Mater Research Institute, University of Queensland, St. Lucia, Queensland, Australia; 7 Burns Trauma and Critical Care Research Centre, University of Queensland, St. Lucia, Queensland, Australia; 8 Department of Intensive Care Medicine, Royal Brisbane & Women’s Hospital, Herston, Queensland, Australia; 9 Emphron Informatics, Toowong, Queensland, Australia; 10 Department of Intensive Care, University Medical Center Utrecht, Utrecht, the Netherlands; 11 Department of Medical Microbiology, University Medical Center Utrecht, Utrecht, the Netherlands; 12 Center of Experimental and Molecular Medicine, Academic Medical Center, Amsterdam, the Netherlands; 13 Division of Infectious Diseases, Academic Medical Center, Amsterdam, the Netherlands; 14 Department of Intensive Care Medicine, Academic Medical Center, Amsterdam, the Netherlands; 15 Laboratory of Experimental Intensive Care and Anesthesiology, Academic Medical Center, Amsterdam, the Netherlands; University College London, UNITED KINGDOM

## Abstract

**Background:**

Systemic inflammation is a whole body reaction having an infection-positive (i.e., sepsis) or infection-negative origin. It is important to distinguish between these two etiologies early and accurately because this has significant therapeutic implications for critically ill patients. We hypothesized that a molecular classifier based on peripheral blood RNAs could be discovered that would (1) determine which patients with systemic inflammation had sepsis, (2) be robust across independent patient cohorts, (3) be insensitive to disease severity, and (4) provide diagnostic utility. The goal of this study was to identify and validate such a molecular classifier.

**Methods and Findings:**

We conducted an observational, non-interventional study of adult patients recruited from tertiary intensive care units (ICUs). Biomarker discovery utilized an Australian cohort (*n* = 105) consisting of 74 cases (sepsis patients) and 31 controls (post-surgical patients with infection-negative systemic inflammation) recruited at five tertiary care settings in Brisbane, Australia, from June 3, 2008, to December 22, 2011. A four-gene classifier combining *CEACAM4*, *LAMP1*, *PLA2G7*, and *PLAC8* RNA biomarkers was identified. This classifier, designated SeptiCyte Lab, was validated using reverse transcription quantitative PCR and receiver operating characteristic (ROC) curve analysis in five cohorts (*n* = 345) from the Netherlands. Patients for validation were selected from the Molecular Diagnosis and Risk Stratification of Sepsis study (ClinicalTrials.gov, NCT01905033), which recruited ICU patients from the Academic Medical Center in Amsterdam and the University Medical Center Utrecht. Patients recruited from November 30, 2012, to August 5, 2013, were eligible for inclusion in the present study. Validation cohort 1 (*n* = 59) consisted entirely of unambiguous cases and controls; SeptiCyte Lab gave an area under curve (AUC) of 0.95 (95% CI 0.91–1.00) in this cohort. ROC curve analysis of an independent, more heterogeneous group of patients (validation cohorts 2–5; 249 patients after excluding 37 patients with an infection likelihood of “possible”) gave an AUC of 0.89 (95% CI 0.85–0.93). Disease severity, as measured by Sequential Organ Failure Assessment (SOFA) score or Acute Physiology and Chronic Health Evaluation (APACHE) IV score, was not a significant confounding variable. The diagnostic utility of SeptiCyte Lab was evaluated by comparison to various clinical and laboratory parameters available to a clinician within 24 h of ICU admission. SeptiCyte Lab was significantly better at differentiating cases from controls than all tested parameters, both singly and in various logistic combinations, and more than halved the diagnostic error rate compared to procalcitonin in all tested cohorts and cohort combinations. Limitations of this study relate to (1) cohort compositions that do not perfectly reflect the composition of the intended use population, (2) potential biases that could be introduced as a result of the current lack of a gold standard for diagnosing sepsis, and (3) lack of a complete, unbiased comparison to C-reactive protein.

**Conclusions:**

SeptiCyte Lab is a rapid molecular assay that may be clinically useful in managing ICU patients with systemic inflammation. Further study in population-based cohorts is needed to validate this assay for clinical use.

## Abstract 2

Thomas Yager and colleagues develop and validate a molecular host assay to discriminate between sepsis and infection-negative systemic inflammation in critically ill patients.

## Editors' Summary

**Background:**

Our immune system protects us from disease by recognizing and killing bacteria and other infectious organisms (pathogens). An important part of the immune response is inflammation, a process that is triggered by infection, tissue injury, and other stimuli. Localized inflammation, which is characterized by swelling, redness, heat, pain, and loss of function, restricts the tissue damage caused by these stimuli to the affected site. Unfortunately, the immune system occasionally initiates a series of reactions that lead to widespread (systemic) inflammation. Systemic inflammation can damage vital organs and can be life-threatening. For example, it has been estimated that 30%–50% of people who develop severe infection-positive systemic inflammation (sepsis) die. Clinical management of systemic inflammation depends on whether the condition is caused by an infection or by another stimulus: many patients with systemic inflammation need to be given corticosteroids and other anti-inflammatory agents, but only patients with infection-positive systemic inflammation need to be given antibiotics.

**Why Was This Study Done?:**

Clinicians need to be able to distinguish between sepsis and infection-negative systemic inflammation quickly and accurately when treating critically ill patients. Patients with sepsis need to be given antibiotics as soon as possible to clear the infection, but giving antibiotics to someone with infection-negative systemic inflammation may do more harm than good. Current diagnostic approaches for identifying patients with sepsis rely on isolating and identifying the causative pathogen, but it can take more than 24 hours to obtain a result and pathogens are only isolated from about 30% of patients with clinically confirmed sepsis. Analysis of the host immune response might provide a quicker, more accurate way to differentiate between sepsis and infection-negative systemic inflammation. Thus, measurement of blood levels of procalcitonin (a pro-inflammatory biomarker) or of C-reactive protein (which the liver releases in response to inflammation) can sometimes differentiate between the two conditions. However, because the immune response is complex, measurement of a single biomarker in the blood is unlikely to be diagnostically helpful in every patient. Here, the researchers identify and validate a set of RNA molecules present in the blood capable of discriminating between sepsis and infection-negative systemic inflammation in critically ill patients (a “molecular classifier”).

**What Did the Researchers Do and Find?:**

Using microarray analysis (a technique that measures the RNA levels of thousands of different genes) to compare the RNA molecules present in the blood in a discovery cohort of 74 patients with sepsis and 31 post-surgical patients with infection-negative systemic inflammation, the researchers identified a molecular classifier (SeptiCyte Lab) consisting of four RNA biomarkers. They validated this classifier using five additional patient cohorts, RT-qPCR (a technique that measures the amounts of specific RNAs in biological samples), and receiver operating characteristic (ROC) curve analysis (a graphical method for determining diagnostic test performance). The overall AUC for the test in the five validation cohorts was 0.88. The AUC (area under a ROC curve) quantifies the ability of a test to discriminate between individuals with and without a disease. A perfect test that yields no false positives or false negatives has an AUC of 1.00; a test no better at identifying true positives than flipping a coin has an AUC of 0.5. Importantly, disease severity did not affect the performance of SeptiCyte Lab, and the assay was better at discriminating sepsis from infection-negative systemic inflammation than all tested clinical and laboratory parameters (singly and in combination) that can currently be obtained within 24 hours of admission to an intensive care unit.

**What Do These Findings Mean?:**

These findings suggest that SeptiCyte Lab might be useful in the management of critically ill patients with systemic inflammation. In the validation cohorts tested, this rapid diagnostic test (SeptiCyte Lab currently takes 4–6 hours to perform but could be developed to provide a result in about 90 minutes) was able to correctly identify 90% of patients with sepsis, although it also mistakenly identified some patients as having sepsis when they had infection-negative systemic inflammation. Further clinical studies are needed before SeptiCyte Lab can be used clinically because of limitations in the current study. For example, the patients in the validation cohorts imperfectly reflect the composition of real-world patients with systemic inflammation. However, the researchers suggest that, in combination with other clinical parameters and clinical judgment, SeptiCyte Lab might help physicians make appropriate therapeutic decisions for patients with systemic inflammation.

**Additional Information:**

This list of resources contains links that can be accessed when viewing the PDF on a device or via the online version of the article at http://dx.doi.org/10.1371/journal.pmed.1001916.

- The UK National Health Service Choices website provides information on sepsis
- The US National Institute of General Medical Sciences provides a fact sheet on sepsis
- The UK Sepsis Trust is a not-for-profit organization that provides information and personal stories about sepsis
- The World Sepsis Day website also provides information and personal stories about sepsis
- MedlinePlus provides links to further information about sepsis (in English and Spanish)
- Wikipedia has pages on sepsis, systemic inflammatory response syndrome, and ROC curve analysis (note that Wikipedia is a free online encyclopedia that anyone can edit; available in several languages)

## Introduction

Patients in the early stages of sepsis are often very difficult to distinguish from patients who have infection-negative systemic inflammation. Making an incorrect distinction between these two clinical presentations has significant clinical and economic ramifications. Incorrect diagnosis can lead to inappropriate patient management, overprescription of antibiotics, and, in worst-case scenarios, patient death or long-term debilitation [1,2].

Diagnostic approaches for identifying sepsis patients are generally based on either pathogen detection or evaluation of host response using biomarkers [3]. The mainstay and de facto “gold standard” for diagnosis of most bacterial infections, including sepsis, is microbial growth of a causative pathogen followed by taxonomic identification. However, culture-based methods suffer from multiple limitations [4–6]: (1) positive results usually take ≥24 h; (2) in clinically confirmed sepsis cases, positive cultures are produced in only ~1/3 of blood cultures and ~2/3 of all cultures from any site including blood [7,8], and, consequently, negative culture results cannot be interpreted definitively; (3) there is a reduced chance of positive culture if the patient is already on antibiotics; (4) interpretation is confounded by false positives produced by contaminants; and (5) positive blood cultures can result from transient bacteremia in the absence of a severe inflammatory response [9]. Thus, culture methods by themselves have inadequate sensitivity, specificity, and predictive value for diagnosing sepsis [10,11].

Analysis of the host immune response provides an alternative approach to diagnosing sepsis [3]. Perhaps the most studied host response biomarker is procalcitonin (PCT), which reportedly differentiates sepsis from infection-negative systemic inflammation [12,13], although growing evidence suggests PCT does not deliver definitive diagnoses [14–16]. Because of the inherent complexity of the host response, a single biomarker with sufficient accuracy for identifying sepsis or stratifying patients for particular treatments may not exist [17]. Recognizing this difficulty, researchers (including our group) have turned to investigating panels of biomarkers for interrogation of the host response [18–22].

We hypothesized that a molecular classifier based on a small number of RNAs expressed in peripheral blood could be discovered that would (1) determine which patients with systemic inflammation had sepsis, (2) be robust across independent patient cohorts, (3) be insensitive to disease severity, and (4) provide diagnostic utility. In the present study we characterize SeptiCyte Lab, a four-gene classifier for discriminating sepsis from infection-negative systemic inflammation in critically ill patients. The classifier was discovered through analysis of an Australian cohort of cases (confirmed or probable sepsis) versus controls (post-surgical patients with infection-negative systemic inflammation). It was then converted from microarray to reverse transcription quantitative polymerase chain reaction (RT-qPCR) format, and validated on five additional independent, heterogeneous cohorts of patients from the Netherlands.

## Methods

### Patient Recruitment and Ethics Statements

#### Discovery phase

Cases and controls were recruited from the intensive care units (ICUs) of five tertiary care settings (Wesley Hospital, Mater Adult Hospital, Mater Private Hospital, Princess Alexandra Hospital, and Royal Brisbane & Women’s Hospital) within the Brisbane, Australia, metropolitan area. The patients were recruited within two formal clinical studies designated GCP-1 (Australian Department for Health and Ageing CTN number 044/2008) and RTT (Australian New Zealand Clinical Trials Registry identifier ACTRN12610000465055). Ethics approvals for the GCP-1 study were conferred by the UnitingCare Health Human Research Ethics Committee (HREC) on behalf of the Wesley Hospital (reference number 20081), the Royal Brisbane & Women’s Hospital HREC (reference number 2008/141), and the Mater Health Services HREC (reference numbers 1095A, 1192A/P, 1192A). Ethics approvals for the RTT study were conferred by the Royal Brisbane & Women’s Hospital HREC (reference number HREC/09/QRBC/295), the Mater Heath Services HREC (reference number 1400AP), and the Metro South Hospital and Health Services HREC on behalf of the Princess Alexandra Hospital (reference number HREC/10/QPAH/246). The study protocols were finalized and the requisite ethics approvals were obtained prior to the recruitment of patients in each study. Summaries of the GCP-1 and RTT study protocols are available in S1 Text. All study participants provided written informed consent either as individuals or through surrogate decision-makers.

#### Validation phase

The MARS study recruited ICU patients across two tertiary teaching hospitals: the Academic Medical Center in Amsterdam and the University Medical Center Utrecht. Patients in the MARS study consisted of adults admitted to the ICU, with the exclusion of cardiac elective surgery patients with an uncomplicated short stay. Patients admitted to the ICU and enrolled in the MARS study over the period November 30, 2012, to August 5, 2013, were eligible for inclusion in the present study, by virtue of having donated additional blood samples in PAXgene Blood RNA tubes that were made available to Immunexpress for analysis. The medical ethical committees of both study centers gave approval for an opt-out consent method (institutional review board approval number 10-056C). Patients and their representatives were informed about the project via brochures handed out at ICU admission and were given an opt-out card that could be completed if participation was declined [24,25]. All patient data were encrypted for privacy reasons.

### Discovery Cohort

All study participants in the discovery cohort were recruited from ICUs in the above hospitals and were 18 y or older. Patients were excluded if they had body mass index ≥ 40 kg/m^2^; displayed any systemic immunological disorders; were transplant recipients, currently receiving chemotherapy treatment for cancer, or immunosuppressed for any other known reason; or had chronic localized bacterial or fungal infections.

Patients were recruited sequentially at each study site, subject to the stated inclusion and exclusion criteria. Patients for the GCP-1 study were recruited from June 3, 2008, to July 21, 2009, and patients for the RTT study were recruited from May 21, 2010, to December 22, 2011. Final diagnosis of sepsis or infection-negative systemic inflammation was made by retrospective physician assessment using all available clinical and microbiological data and according to the American College of Chest Physicians/Society of Critical Care Medicine consensus statement for sepsis [23]. Consensus evaluations were made by two ICU physicians for the GCP-1 study and four ICU physicians for the RTT study, and were completed before microarray analysis of blood samples was initiated. Microarrays for the two studies were run over the period from November 3, 2011, to April 4, 2012.

Each patient’s demographic parameters, vital signs, hematology, clinical chemistry, and blood pathogen detection results were recorded. Multiple blood samples for clinical chemistry, hematology, and gene expression analyses were collected within 24 h of the surgical procedure for the control group, or within 24 h of ICU admission for the sepsis group. Blood samples (2 × 2.5 ml) for gene expression analysis were collected into PAXgene Blood RNA tubes (PreAnalytiX).

### Validation Cohorts

Patients for the validation cohorts were selected from the Molecular Diagnosis and Risk Stratification of Sepsis (MARS) study (ClinicalTrials.gov, NCT01905033), a prospective observational cohort study in the Netherlands designed to produce molecular information relevant to sepsis diagnosis and management. A summary of the MARS study is available in S1 Text. The MARS study recruited ICU patients across two tertiary teaching hospitals: the Academic Medical Center in Amsterdam, and the University Medical Center Utrecht. Patients in the MARS study consisted of adults admitted to the ICU, with the exclusion of cardiac elective surgery patients with an uncomplicated short stay. As described above, patients admitted to the ICU and enrolled in the MARS study from November 30, 2012, to August 5, 2013, were eligible for inclusion in the present study. All relevant clinical, microbiological, interventional, and demographic data was stored in a database after multiple quality control steps.

For patients enrolled in the MARS study, a sepsis event was defined *operationally* to have occurred when a patient displayed two or more signs of systemic inflammation and was given therapeutic systemic antibiotics by the attending physician within 24 h of ICU admission. In other words, a sepsis event was deemed to have occurred when the ICU clinician had sufficient suspicion of sepsis to prescribe therapeutic systemic antibiotics. In some instances, a sepsis event was adjudicated retrospectively to have occurred several days before ICU admission but to have been unrecognized at the time of occurrence. Patients having a sepsis event within the interval from 3 d before ICU admission to 2 d after ICU admission were considered for inclusion in the present study. Patients were excluded if the sepsis event nearest to ICU admission fell outside this interval. For the sepsis event nearest to ICU admission, a physician-assessed infection likelihood of none, possible, probable, or definite was assigned retrospectively according to US Centers for Disease Control and Prevention and International Sepsis Forum consensus definitions [4,24,26]. See S2 Text for additional detail.

Patients in each of the five validation cohorts were classified as either cases (sepsis) or controls (infection-negative systemic inflammation). Patients were classified as cases if they experienced a sepsis event and were then adjudicated to have an infection likelihood of probable or definite for that event. Patients were classified as controls if (1) they displayed two or more symptoms of systemic inflammation but were never given therapeutic systemic antibiotics (i.e., did not have a sepsis event) or (2) they displayed two or more symptoms of systemic inflammation and were given therapeutic systemic antibiotics (i.e., operationally defined to have had a sepsis event) but were then retrospectively adjudicated to have had an infection likelihood of none. Patients were assigned an infection likelihood of possible if they operationally had a sepsis event but upon retrospective adjudication could not be classified with certainty as either a case or control. These patients were excluded from performance analyses but included in an analysis of factors leading to classification uncertainty.

Peripheral blood samples were collected from each patient at <24 h after admission to the ICU. Routine hematology and biochemistry were performed as part of patient management. PCT was measured retrospectively, on frozen blood samples, using the Vidas B.R.A.H.M.S. PCT test (bioMérieux). Per the manufacturer’s instructions, PAXgene Blood RNA tubes (PreAnalytiX) were kept at room temperature for 2 h, then transferred to −20°C overnight, and finally transferred to −80°C, where they were stored until workup.

All final classification of patients as either cases or controls was completed within 3 mo of ICU admission and before gene expression analysis of blood samples was initiated. The precise dates of ICU admission and data generation for the validation cohorts were as follows: validation cohort 1: ICU admission December 7, 2012, to March 25, 2013, data generated in July 2013; validation cohort 2: ICU admission December 2, 2012, to July 20, 2013, data generated in July and October 2013 (two batches); validation cohort 3: ICU admission December 3, 2012, to July 13, 2013, data generated in November 2013; validation cohort 4: ICU admission March 16, 2013, to June 18, 2013, data generated in August 2014; validation cohort 5: ICU admission November 30, 2012, to August 5, 2013, data generated in April 2014.

### Purification of RNA from PAXgene Blood Samples

PAXgene Blood RNA tubes were shipped on dry ice to Asuragen (Austin, Texas, US), where total RNA was isolated on a KingFisher Flex Magnetic Particle Processor (ThermoFisher Scientific). Tubes were thawed for 16 h at room temperature. After centrifugation and washing to collect cell pellets, cells were lysed in a guanidinium-containing buffer. Organic extraction was performed prior to adding binding buffer and magnetic beads in preparation for the KingFisher run. The RNA isolation procedure included a DNase treatment step and cleanup prior to elution from the magnetic beads.

The purity and quantity of the purified RNA samples were determined by absorbance readings at 260 nm/280 nm using a NanoDrop ND-1000 UV spectrophotometer (ThermoFisher Scientific). For RT-qPCR analysis, the extracted RNA was considered suitable if the yield was ≥2 ng/μl in a final extraction volume of 80 μl. For microarray analysis, additional checks on RNA integrity were performed by microfluidic electrophoresis on (1) an Agilent Bioanalyzer 2100, using the Agilent Bioanalyzer 2100 Nano Assay, or (2) a Caliper LabChip system (Agilent Technologies). RNA preparations were considered suitable for microarray profiling if the A_260_/A_280_ ratio was >1.6 and the RNA integrity number (Agilent) or RNA quality score (Caliper) was >5.

### Data Acquisition and Analysis

#### Microarrays (discovery phase)

Biotin-labeled sense strand cDNA was prepared from 300 ng of total RNA per sample using a modified Affymetrix GeneChip Whole Transcript Sense Target Labeling Assay. Yields of intermediate cRNA and final cDNA were quantified by UV spectrophotometry. Fragmentation and labeling of cDNA was performed in 5-μg aliquots. Hybridization to Affymetrix Human Exon 1.0 ST arrays was carried out at 45°C for 16 h in an Affymetrix model 640 hybridization oven. Arrays were washed and stained on an Affymetrix Fluidics Station 450. The arrays were scanned on an Affymetrix GeneChip Scanner 3000 7G, and for each scanned array a set of DAT, CEL, JPG, and XML flat files were generated.

Microarray datasets were processed in batches using Affymetrix Power Tools (APT) and normalized using the robust multichip average method. Background was corrected using detection above background (DABG) *p*-values and normalized using the quantile method. The analysis considered only those probe sets that were defined in the Affymetrix core dataset, which consisted of >30,000 transcripts annotated using RefSeq and processed by the APT software [27].

Quality metrics were based on analysis of all probe sets, bacterial spike-ins, poly(A) spike-ins, and positive and negative control probe sets. These metrics form the set of residuals for each probe, from the robust multichip average probe set model. The mean absolute deviation of these residuals was then calculated for each probe set, and the mean of the mean absolute deviations was then calculated for all probe sets in the collection. Identification of outlier arrays was made separately on each subset of data as it became available. As array data became available from batched processing, they were iteratively added to the dataset, and the entire APT quality control process was repeated to include comparison of the new included dataset against robust estimates of mean and standard deviation of the mean for each chip in the historical dataset [28]. Transcripts were considered to be differentially expressed between two samples if each signal had >100 intensity units and the fold change was >2.0.

Microarray-derived classifiers to distinguish cases from controls were based on relative hybridization signal intensity levels between probe sets in the microarray data. Candidate genes were selected by initial analyses with machine learning enrichment techniques, including recursive feature elimination support vector machines [29] and backwards elimination random forests [30]. Classifiers involving gene combinations were developed iteratively over many subsets of the samples using the machine learning methods cited above. Genes that consistently appeared in multiple classifiers were deemed to be informative, while genes appearing in few or no classifiers were deemed to be uninformative.

Individual gene candidates that survived the above selection process were then reanalyzed in terms of intensity (*I*) ratios:

$${\text{ratio}}_{A,B}=\log\left(I\left[\text{transcript}\;A\right]\right)-\log\left(I\left[\text{transcript}\;B\right]\right)$$ (1)

This “fold-change ratio transform” reveals properties of the underlying system that are not captured using standard tests on the raw gene expression values. An analogy to this value-adding transform step is the Fourier transform, which has common applications in many fields of science and engineering and is often used to build classifiers that perform better or more simply in the transformed space than on the raw data [31].

All two-gene ratios defined by Eq 1 were analyzed by receiver operating characteristic (ROC) curves and ranked by area under curve (AUC) for their ability to separate cases from controls [32]. In total, 11,100 two-gene ratios were generated by the above procedure and ranked according to AUC.

A greedy search [33] was then employed in an additive model that began with the best two-gene ratio and then added another two-gene ratio from a pool of top-ranking candidates. A number of candidate ratio pairs were generated, and combined scores were calculated:

$${\text{comb}}_{A,B,C,D}={\text{ratio}}_{A,B}+{\text{ratio}}_{C,D}$$ (2)

ROC curve analysis was used to evaluate the performance of two-ratio classifiers as defined by Eq 2. This approach allowed the identification of high-performing classifiers having fewer genes than classifiers identified by an approach we used previously, LogitBoost regression [21]. LogitBoost regression resulted in classifiers that measured the absolute values of each of the input genes. In contrast, the present method transformed the data into relative expression levels between pairs of genes before selecting classifier elements. For the above ROC analyses, binormal smoothing was used for ROC curve generation. A resampling method was used to estimate the 95% confidence interval (CI) of the AUC associated with each ROC curve. AUC and CI values were reported to two significant figures. Venkatraman’s method [34], as implemented in the pROC package in R, was used to compare the AUC values between different classifiers; *p-*values for the comparisons were calculated to two significant figures, with *p <* 0.05 considered statistically significant. In this context, AUC is used only as the objective function of the greedy search algorithm and does not imply future performance against independent datasets.

Of all the two-ratio classifiers generated, the classifier with highest AUC was designated SeptiCyte Lab, as specified by Eq 3:

SeptiCyte Lab=log(I[PLAC8]/I[PLA2G7])+log(I[LAMP1]/I[CEACAM4])=log(I[PLAC8])−log(I[PLA2G7])+log(I[LAMP1])−log(I[CEACAM4]) (3)

As a final step, ROC curve analysis was used to test the SeptiCyte Lab classifier against an independent, publicly available dataset (E-MTAB-1548 from the EMBL-EBI ArrayExpress database). This dataset presents mRNA profiles from PAXgene Blood RNA samples collected in Spain from 39 cases (post-surgical patients with septic shock) versus 34 controls (patients with systemic inflammatory response syndrome).

#### RT-qPCR (validation phase)

By analogy to the two-ratio microarray classifier defined by Eq 3, the output of SeptiCyte Lab in terms of RT-qPCR is a quantitative score:

$$\text{SeptiScore}=-C_{\text{t},1}+C_{\text{t},2}+-C_{\text{t},3}+C_{\text{t},4}$$ (4)

where *C*_t_ is the threshold cycle number for *PLAC8* (*C*_t,1_), *PLA2G7* (*C*_t,2_), *LAMP1* (*C*_t,3_), and *CEACAM4* (*C*_t,4_).

Note the inverse relationship between intensity (in microarray data) and *C*_t_ value (in RT-qPCR data), which explains the difference in signs between Eqs 3 and 4. The coefficients used to combine the four *C*_t_ values to produce the SeptiScore were restricted to +1, −1.

#### Multiple platforms

Data were acquired on multiple platforms as follows: discovery cohort on Affymetrix microarrays; validation cohort 1 on the Applied Biosystems (ABI) 7900HT Fast Real-Time PCR System, using TaqMan Low Density Array cards; validation cohorts 2, 3, and 5 on the ABI 7500 Fast Real-Time PCR system using Life Technologies TaqMan Gold RT-qPCR chemistry in strip tubes; and validation cohort 4 on the ABI 7500 Fast Real-Time PCR system using Asuragen RT-qPCR chemistry in strip tubes. All quantitative PCR reactions were singleplex. S1 Data presents the primers, probes, dyes, and quenchers for the four singleplex quantitative PCR assays; the reverse transcription and quantitative PCR buffers and thermal cycling programs; and also a series of comparison tests from which linear shift formulae were derived for comparing and combining data across platforms. SeptiScores were adjusted to values that would be observed with the Asuragen RT-qPCR chemistry, using the linear shift formulae specified in S1 Data.

### Statistical Analyses

#### ROC curves

For all classifiers, accuracy of classifying cases versus controls was evaluated by ROC curve analysis, with AUC used to quantify performance. AUCs and 95% CIs were computed by resampling using the pROC package, version 1.5.4 [35], and were reported to two significant figures. Differences in AUC between pairs of ROC curves were evaluated for significance by Venkatraman’s method [34] for microarray data from the discovery cohort, and by DeLong’s test [36] for RT-qPCR data from the validation cohorts; *p*-values for the comparisons were calculated to two significant figures, with *p <* 0.05 considered statistically significant. We did not use 2 × 2 contingency tables as the primary method of assessing performance to avoid loss of any diagnostic information contained in the full ROC curves [37–40].

#### Other measures of assay performance

Besides ROC curves, we also evaluated assay performance with other measures derived from 2 × 2 contingency tables. We assumed a cutoff of 3.100 for the SeptiScore, which biases toward sensitivity at the expense of specificity. We then calculated accuracy, sensitivity, specificity, positive predictive value (PPV), negative predictive value (NPV), positive likelihood ratio (LR+), and negative likelihood ratio (LR−) [41]. Similar calculations were performed for PCT assuming a cutoff value of 2 ng/ml. Finally, we also computed the net reclassification index for positive samples (NRI+) and the net reclassification index for negative samples (NRI−) [42,43], which describe the gain in classification performance when SeptiCyte Lab is substituted for PCT. Calculation of these parameters, along with their 95% CIs and *p*-values, was performed using the R package DTComPair, version 1.00 [44]; values are reported to two significant figures.

#### Comparison of statistical distributions

Cumulative distributions were compared with the Kolmogorov-Smirnov (KS) test. Frequency distributions were compared with a *t*-test if normally distributed, or with a Wilcoxon rank-sum test if not normally distributed. Binomial 95% CIs for the evaluation of imbalances in the demographic characteristics of the discovery and validation cohorts were calculated using the online calculator available at http://statpages.org/confint.html.

#### Multivariate analysis

We compared the diagnostic performance of SeptiCyte Lab to that of various combinations of clinical and laboratory parameters that were readily available within 24 h of ICU admission and that might be used as a basis for diagnosing sepsis [23]. This analysis was performed on the combination of validation cohorts 1, 3, and 5, with patients having an infection likelihood of possible removed from cohort 5 (total number of patients analyzed = 211). (Validation cohorts 2 and 4 were not included because PCT data were not available for these cohorts.) Clinical parameters were tested individually for significance in differentiating cases from controls, and those with *p* > 0.05 were excluded. A forward logistic regression process using a greedy search algorithm [33] was then used to sequentially add the best-performing clinical parameters into alternate models. This approach gave stepwise maximum incremental improvement with the addition of each parameter. Potential overfitting was mitigated by conducting a 50 × 2 cross-validation with training and test samples selected in a 1:1 ratio. Some logistic models, as indicated, were deliberately constrained to either exclude or include SeptiCyte Lab or PCT.

### Data Integrity and Accessibility

The STARD checklist [45,46] was completed successfully, with no significant deviations or omissions, and is available as S3 Text. The microarray data generated in the discovery phase have been uploaded to the Gene Expression Omnibus under the identifier GSE74224. An overlapping set of microarray data was previously uploaded under the identifier GSE28750. The complete line data for both the discovery phase and the validation phase are available in S2 Data. All data have been patient-deidentified.

## Results

### Discovery Cohort

The discovery cohort (*n* = 105) consisted of consecutively enrolled patients classified (as described above) as either cases (*n* = 74) or controls (*n* = 31). Characteristics of the discovery cohort are described in Table 1.

**Table 1:** Characteristics of the discovery cohort.

Characteristic	Controls (*n* = 31)	Cases (*n* = 74)	Total (*n* = 105)	Statistical Significance of Any Differences between Groups
**Number of patients, *n* (percent of total)** ^1^	31 (100.0%)	74 (100.0%)	105 (100.0%)	Case and control groups are unbalanced with respect to size.^2^
**Patient age (y)**				Case and control groups have different age distributions (*p* = 0.007; *t*-test).
Median	66	62.5	64	
Interquartile range	60–71	46–71	54–71	
Range	51–86	20–89	20–89	
**Patient sex male, *n* (percent of total)**	22 (71.0%)	41 (55.4%)	63 (60.0%)	Case and control groups are unbalanced with respect to sex.^2^
**Patient race, *n* (percent of total)**				Distribution of white and non-white individuals matches the racial distribution for Australia.^2^ ^,^ ^3^
White	31 (100.0%)	67 (90.5%)	98 (93.3%)	
Asian/East Indian	0 (0%)	3 (4.0%)	3 (2.9%)	
Black	0 (0%)	0 (0%)	0 (0%)	
Aboriginal and Torres Strait Islander	0 (0%)	4 (5.4%)	4 (3.8%)	
**Microbiology, *n* (percent total)**				All proportions except for “blood culture not performed” are significantly different between cases and controls.^2^
Blood culture not performed	1 (3.2%)	1 (1.4%)	2 (1.9%)	
Blood culture positive	0 (0%)	25 (33.8%)	25 (23.8%)	
Blood culture negative	30 (96.8%)	48 (64.9%)	78 (74.2%)	
Gram-positive isolations^4^	0 (0%)	45 (60.8%)	45 (42.8%)	
Gram-negative isolations^4^	0 (0%)	41 (55.4%)	41 (39.0%)	
Fungal isolations^4^	0 (0%)	18 (24.3%)	18 (17.1%)	
Mixed infections^5^	0 (0%)	32 (43.2%)	32 (30.5%)	
**PCT (ng/ml)**				*p <* 0.001 (Mann-Whitney U test)
Median	0.05	2.90		
Interquartile range	0.05 to 0.06	0.36 to 20.1		
Range	<0.05 to 7.83	<0.05 to >200		

^1^The discovery cohort in the present study consisted of 74 cases and 31 controls. This differs from a poster presented at the Paris Sepsis Forum 2012 [22], which was based on the same Australian clinical studies and which claimed to report on 87 cases and 31 controls. The cohort in the present study was subject to additional rigorous exclusion criteria and data quality checks, and, consequently, the number of patients in the present cohort is reduced compared to the Paris Sepsis Forum 2012 analysis From an initial set of 144 patients, the following 39 exclusions were made. Four patients were excluded on the basis of systemic immunological disorders: one patient with Stevens-Johnson syndrome, one with coeliac disease, and two with Hashimoto disease. One patient was excluded for having a chronic bacterial/fungal infection localized to one toe, combined with normal vital signs and blood count. Nine patients were excluded because they dropped out of the trials early, because blood was collected prior to surgery, or because they could not be diagnosed unambiguously as control or sepsis. Twenty-five patients were excluded for technical reasons relating to failed or substandard sample extraction, cRNA or cDNA preparation, or microarray data quality.

^2^Case and control groups were considered imbalanced if the observed values (*n*_case_, *n*_control_) fell outside the 95% CI for a binomial distribution centered on (*n*_case_ + *n*_control_)/2.

^3^Data for Australia from http://www.statista.com.

^4^Isolation from any clinical material including blood culture.

^5^A mixed infection consists of at least two of the three categories of Gram-positive bacteria, Gram-negative bacteria, and fungi.

#### Culture results

Blood cultures were performed for 97% (30/31) of the controls; all were negative. Of the cases, 99% (73/74) had blood cultures performed, and of these 34% (25/73) were positive. Sixty-six patients in the discovery cohort had one or more bacteria or fungi isolated from any culture material including blood, urine, lavage, aspirate, or swab. Of these, 68% (45/66) had one or more Gram-positive bacteria isolated, 62% (41/66) had one or more Gram-negative bacteria isolated, 27% (18/66) had one or more fungi isolated, and 48% (32/66) had mixed infections involving at least two classes of pathogen. From an examination of patient records, it appears that at most one of the 18 fungal infections was a blood infection; the remainder appear to be opportunistic infections at other body sites.

#### Heterogeneity of cases (septic patients)

Besides the specific exclusions listed in footnote 1 of Table 1, no patients were excluded based on predisposing condition, organ system affected, co-morbidities, presence of natural immunosuppression or immunosenescence, therapies, or type of pathogen detected. Patients with both systemic and local infections were included. A wide variety of pathogens were identified, including Gram-positive bacteria, Gram-negative bacteria, fungi, and mixed infections. Viral infections were generally not tested for.

### Validation Cohorts

**Fig 1:**
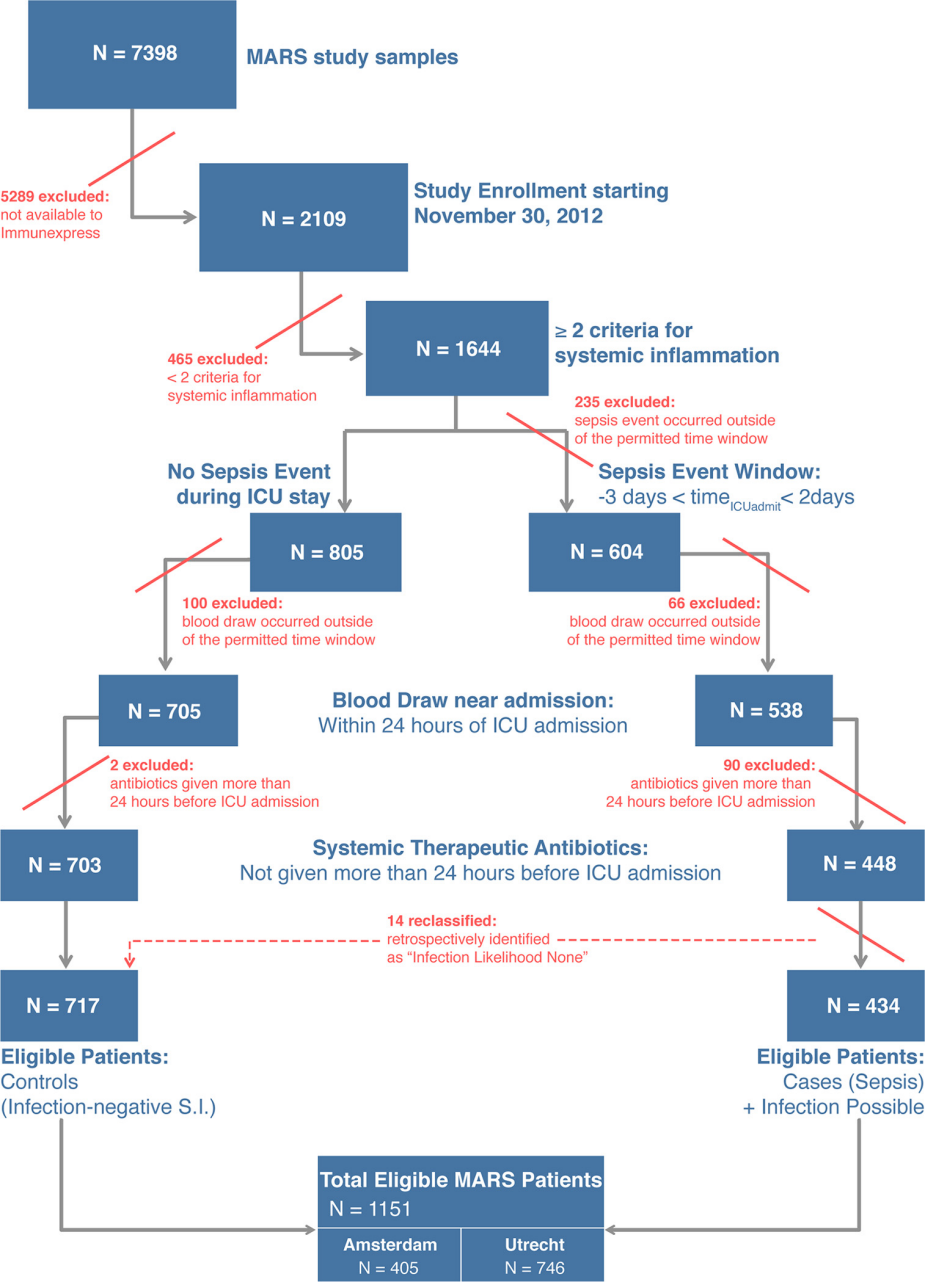
Flow diagram for selection of patients composing the validation cohorts. From the MARS study, only patients admitted to the ICU between the dates of November 30, 2012, and August 5, 2013, were eligible for possible inclusion in the present study. A detailed description of the inclusion and exclusion criteria, and the classification algorithm, is given in S2 Text. In the last step of the cohort selection process as described in this figure, 14 patients were reassigned as controls because the attending physicians had retrospectively adjudicated the patients to have an infection likelihood of none for their sepsis events. S.I., systemic inflammation.

A total of 345 patients from the MARS study were selected for analysis in the present study. Fig 1 presents a flow diagram indicating the inclusion and exclusion criteria used for patient selection, and the individual selection steps whereby patients were excluded. The selection process led to definition of five validation cohorts comprising 59, 36, 106, 87, and 57 patients (described below), which were chosen for different purposes and accordingly had different clinical and demographic characteristics (Table 2; S2 Text). A mapping of the patients in the validation cohorts back to the MARS study sites is given in S2 Text.

**Table 2:** Characteristics of the validation cohorts.

Characteristic	Cohort 1		Cohort 2		Cohort 3		Cohort 4		Cohort 5		Statistical Significance of any Differences Between Groups
	Controls	Cases	Controls	Cases	Controls	Cases	Controls	Cases	Controls	Cases	
**Patient number, *n* (percent of total)** ^1^	35 (59.3%)	24 (40.7%)	27 (90.0%)	3 (10.0%)	77 (72.6%)	29 (27.4%)	47 (70.1%)	20 (29.9%)	25 (54.3%)	21 (45.7%)	Cohorts 2,3, and 4 display significant size imbalances between case and control groups.^2^
**Patient age (y)**											No significant difference in age distribution between case and control groups, for any cohort (*t-*test).
Median	64	65	62	59	55	54	64	61	51	56	
Interquartile range	55–71	61–68	52–68	52–65	46–66	49–66	52–72	53–69	34–72	43–63	
Range	21–83	36–82	21–82	45–71	18–94	38–80	19–99	26–76	21–78	25–79	
**Patient sex male, *n* (percent of total)**	21 (60.0%)	13 (54.2%)	20 (74.1%)	1 (33.3%)	35 (45.4%)	17 (58.6%)	23 (48.9%)	13 (65.0%)	16 (64.0%)	8 (38.1%)	Cohort 2 displays a significant male/female imbalance.^2^
**Patient race, *n* (percent of total)**											No significant difference in proportion of white individuals between case and control groups (cohorts 1–4) or in proportion of black individuals between case and control groups (cohort 5) (Fisher’s exact test).
White	33 (94.3%)	23 (95.8%)	27 (100.0%)	2 (66.6%)	72 (93.5%)	24 (82.8%)	46 (97.9%)	20 (100.0%)	0	0	
Asian	2 (5.7%)	0	0	0	1 (1.3%)	1 (3.4%)	0	0	13 (52.0%)	5 (23.8%)	
Black	0	0	0	1 (33.3%)	2 (2.6%)	4 (13.8%)	0	0	12 (48.0%)	16 (76.2%)	
Other/not recorded	0	1 (4.2%)	0	0	2 (2.6%)	0	1 (2.1%)	0	0	0	
**Weight (kg)**											No significant difference in weight distribution between case and control groups, for any cohort (*t*-test).
Median	80	80	85	72	75	80	75	84	70	70	
Interquartile range	75–89	71–90	76–94	64–86	66–85	65–90	66–92	69–94	58–85	65–79	
Range	46–120	60–153	56–110	55–110	36–153	49–120	45–130	44–130	48–110	40–130	
**White cell count (number/μl) × 10^-3^**											A significant difference in white cell count between case and control groups, for cohort 4 only (*p* < 0.05, *t*-test).
Median	11.8	12.2	14.2	12.4	13.2	10.6	11.2	14.5	12.0	10.2	
Interquartile range	8.4–15.2	6.4–16.4	11.2–18.2	11.9–41	9.0–16.4	6.8–15.1	8.2–16.4	10.8–22.8	9.4–15.9	5.6–13.4	
Range	6.6–25	1.2–31.9	9.4–32.6	11.8–52.7	3.4–35.3	2.2–23.3	2.7–40.5	5.1–35.1	7.5–28.0	2.2–34.9	
**Lactate (mM)**											No significant difference in lactate distribution between case and control groups, for any cohort (*t*-test).
Median	2.2	2.8	3.9	3.4	2.6	2.5	2.4	1.6	3.2	2.5	
Interquartile range	1.3–3.6	1.5–4.7	2.6–5.4	2.8–4.6	1.6–4.2	1.7–3.9	1.4–3.8	1.3–3.1	1.5–5.0	2.0–6.0	
Range	0.7–9.3	0.8–9.9	1.1–17.2	2.1–5.8	0.8–17.4	0.7–25.9	0.5–11.5	0.7–8.9	0.7–13.3	1.1–20.1	
**PCT (ng/ml)**											A significant difference in PCT distribution between case and control groups was found for cohort 1 (*p <* 0.008), cohort 3 (*p <* 0.003), cohort 4 (*p <* 0.005), and cohort 5 (*p <* 0.007) by *t*-test. The *t-*test could not be done for cohort 2.
Median	0.4	4.7	1.9	NA	0.3	9.5	0.5	7.4	2.1	9.5	
Interquartile range	0.1–0.9	0.7–30.1	0.6–6.9	NA	0.1–1.0	1.0–32.2	0.2–2.2	2.6–18.9	0.6–5.7	2.2–89.2	
Range	0.05–21.0	0.1–230	0.04–166	NA	0.02–190	0.09–150	0.04–41.3	0.2–200	0.04–65.6	0.2–280	
Patients tested	31 (88.6%)	19 (79.2%)	15 (55.6%)	0 (0.0%)	70 (90.9%)	27 (93.1%)	41 (87.2%)	19 (95.0%)	20 (80.0%)	20 (95.2%)	
Patients not tested	4 (11.4%)	5 (20.8%)	12 (44.4%)	3 (100.0%)	7 (9.1%)	2 (6.9%)	6 (12.8%)	1 (5.0%)	5 (20.0%)	1 (4.8%)	
**CRP (mg/l)**											A significant difference in CRP distribution between case and control groups was found for cohort 1 (*p <* 0.001), cohort 3 (*p <* 0.001), cohort 4 (*p <* 0.025), and cohort 5 (*p <* 0.012) by *t-*test.
Median	9.0	96	18	NA	7.0	109	49	249	13	183	
Interquartile range	4.0–62	47–246	3.2–50	NA	3.0–18	38–244	7.5–79	130–351	5.5–21	75–262	
Range	1.0–297	1.0–480	1.0–294	NA	1.0–208	2.0–358	2.0–167	11–453	1.0–71	31–438	
Patients tested	31 (88.6%)	24 (100.0%)	18 (66.7%)	0 (0.0%)	47 (61.0%)	15 (51.7%)	10 (21.3%)	3 (15.0%)	6 (24.0%)	11 (52.4%)	
Patients not tested	4 (11.4%)	0 (0.0%)	9 (33.3%)	3 (100.0%)	30 (39.0%)	14 (48.3%)	37 (78.7%)	17 (85.0%)	19 (76.0%)	10 (47.6%)	
**APACHE IV score**											A significant difference in Apache IV score distribution between case and control groups was found for cohort 1 (*p <* 0.03) and cohort 5 (*p <* 0.03) by *t-*test.
Median	67	80	67	87	62	76	60	71	62	92	
Interquartile range	48–90	69–94	50–86	83–117	44–88	58–93	45–81	58–88	47–84	71–107	
Range	22–161	51–158	29–155	79–147	20–142	17–149	28–152	38–116	20–144	24–153	
**Affected organ system (*n*)**											
Respiratory	0	13	1	2	0	13	5	6	1	7	
Central nervous system	0	4	0	0	0	1	0	2	0	2	
Cardiovascular	0	1	0	0	1	2	0	5	0	0	
Urinary tract	0	0	1	0	0	4	2	2	1	5	
Abdominal/gastrointestinal	0	4	0	0	0	6	0	3	0	3	
Skin/soft tissue	0	1	0	1	0	1	0	1	0	2	
Post-operative wound	0	0	0	0	0	1	0	0	0	1	
Other/unknown	0	1	0	0	0	1	2	1	0	1	
Data not available	35	0	25	0	76	0	38	0	23	0	
**Positive microbiology results (*n*)**	8/35 (22.8%)	18/24 (75.0%)	8/27 (29.6%)	1/3 (33.3%)	16/77 (20.8%)	21/29 (72.4%)	9/47 (19.1%)	15/20 (75.0%)	6/25 (24.0%)	15/21 (71.4%)	
Gram-positive bacteria	2	13	3	1	4	16	8	8	3	12	
Gram-negative bacteria	6	9	8	1	11	14	1	8	3	10	
Mixed	4	11	4	0	3	9	0	6	1	8	
Fungal	5	11	2	0	5	2	0	6	1	2	

^1^A total of 37 patients with an infection likelihood of possible (six from validation cohort 2, 20 from validation cohort 4, and 11 from validation cohort 5) were excluded from consideration. Accordingly, the calculations in this table were performed using a total sample size *n* = 345 − 37 = 308.

^2^Case and control groups were considered imbalanced if the observed values (*n*_case_, *n*_control_) fell outside the 95% CI for a binomial distribution centered on (*n*_case_ + *n*_control_)/2.

APACHE, Acute Physiology and Chronic Health Evaluation; CRP, C-reactive protein; LB, lower bound; UB, upper bound.

#### Validation cohort 1 (*n* = 59 consisting of 24 cases, 35 controls)

Validation cohort 1 contained only patients diagnosed with high confidence as having either sepsis or infection-negative systemic inflammation. This cohort consisted of patients admitted to the Utrecht (University Medical Center Utrecht) ICU from December 7, 2012, to March 25, 2013, but not sequentially.

#### Validation cohort 2 (*n* = 36 consisting of three cases, 27 controls; six with infection likelihood of possible)

Validation cohort 2 contained patients who were randomly picked from the Amsterdam (Academic Medical Center) ICU (*n* = 19) or Utrecht ICU (*n* = 17) with ICU admission dates spanning the entire time frame of interest (December 2, 2012, to July 20, 2013). This cohort was used to test whether the score generated by SeptiCyte Lab exhibited any bias with respect to ICU admission date.

#### Validation cohort 3 (*n* = 106 consisting of 29 cases, 77 controls)

Validation cohort 3 was drawn from a consecutive sequence of 775 patients admitted to the Amsterdam ICU and Utrecht ICU from December 3, 2012, to July 13, 2013. From this initial set of patients, 91 with an infection likelihood of possible (91/775 = 11.7%) were deliberately excluded. An additional four patients were excluded because insufficient data were captured to meet the minimum reporting requirements for retrospective physician adjudication of infection likelihood. From the remaining pool (*n* = 680), patients were randomly drawn to define this cohort (*n* = 52 from Amsterdam and *n* = 54 from Utrecht).

#### Validation cohort 4 (*n* = 87 consisting of 20 cases, 47 controls; 20 with infection likelihood of possible)

Validation cohort 4 consisted of patients who were consecutively admitted to the Amsterdam ICU from March 16 to June 18, 2013. This cohort was used to assess performance in a real-world setting (i.e., sequential patients).

#### Validation cohort 5 (*n* = 57 consisting of 21 cases, 25 controls; 11 with infection likelihood of possible)

Validation cohort 5 contained exclusively black and Asian patients who were consecutively admitted to the Amsterdam ICU (*n* = 46) or Utrecht ICU (*n* = 11) from November 30, 2012, through August 5, 2013. This cohort was used to determine whether the performance of SeptiCyte Lab was affected by race in a real-world setting (i.e., sequential patients).

### Initial Discovery of the SeptiCyte Lab Classifier

#### High-ranking ratios in microarray analysis

From the Affymetrix core dataset of >30,000 RefSeq-annotated transcripts, the top-ranking ratio was *PLA2G7/PLAC8*, with an AUC of 0.98. In an effort to further improve the AUC, other high-ranking ratios were added to this top ratio. By adding *CEACAM4/LAMP1*, the AUC could be improved to 1.00. It must be stressed that neither the AUC of 0.98 nor the AUC of 1.00 obtained in this stage of analysis should be considered realistic estimates of the performance of this classifier on independent samples. These AUCs are merely the maximized values of the objective function used in the greedy search algorithm.

Highest discriminative power was obtained by combining the four RNA expression values into two ratios, and the addition of other RNA expression values or ratios did not significantly enhance performance. The combination of *PLA2G7/PLAC8* and *CEACAM4*/ *LAMP1* RNA expression ratios, specified by Eq 3, is referred to as the SeptiCyte Lab classifier. Descriptions of these four genes, including their purported biological roles, are summarized in Table 3.

**Table 3:** Genes used in the SeptiCyte Lab classifier.

HGNC Symbol (Entrez Gene ID)	Full Name (Common Aliases)	Mechanism Related to Sepsis
*LAMP1* (3916)	Lysosomal-associated membrane protein 1 (*CD107a*, *LAMPA*, *LGP120*)	Encodes a membrane glycoprotein known to be involved in autophagy, a catabolic host–response mechanism for intracellular bacterial clearance with a hypothesized role in early sepsis and bacterial clearance [47–49]. Provides selectins with carbohydrate ligands. Implicated in tumor cell metastasis. The protein shuttles between lysosomes, endosomes, and the plasma membrane. KEGG pathways: hsa05152 (tuberculosis), hsa04142 (lysosome), hsa04145 (phagosome).
*PLA2G7* (7941)	Phospholipase A2, group VII (platelet-activating factor acetylhydrolase, *PAF-AH*, *LDL-PLA2*)	Encodes the protein PAF-AH (EC 3.1.1.47), a secreted enzyme that catalyzes the degradation of PAF. Alterations in the activity of PAF-AH are hypothesized to contribute to the pathophysiology of sepsis. High plasma levels have been found to correlate with sepsis survival [50], and decreased levels have been found in sepsis [51]. Found extracellularly. KEGG pathways: hsa00565 (ether lipid metabolism), hsa01100 (metabolic pathways). Has been trialled clinically for treatment of sepsis [52–55].
*PLAC8* (51316)	Placenta-specific gene 8 (*C15*, *Onzin*)	Encodes a protein expressed in a variety of immune cells (spleen, lymph nodes), including high expression on plasmacytoid dendritic cells. Is reportedly an interferon-inducible gene [56] with putative roles in the optimal function of neutrophils following the uptake of bacteria [57], the clearance of chlamydia [58,59], and the host response to viral infections [60]. KEGG pathways: no hits.
*CEACAM4* (1089)	Carcinoembryonic antigen-related cell adhesion molecule 4 (*CGM7*, *NCA*)	Encodes a protein expressed by granulocytes [61] with an intracellular immunoreceptor tyrosine-based activation motif (ITAM). Evidence from chimeric protein studies indicates that CEACAM4 is involved in triggering phagocytosis of bacteria [62]. Belongs to the immunoglobulin superfamily. Binds opacity (Opa) protein of Neisseria [63]. Integral to the plasma membrane. KEGG pathways: no hits.

HGNC, Human Genome Organisation Gene Nomenclature Committee; KEGG, Kyoto Encyclopedia of Genes and Genomes; PAF, platelet-activating factor acetylhydrolase.

**Fig 2:**
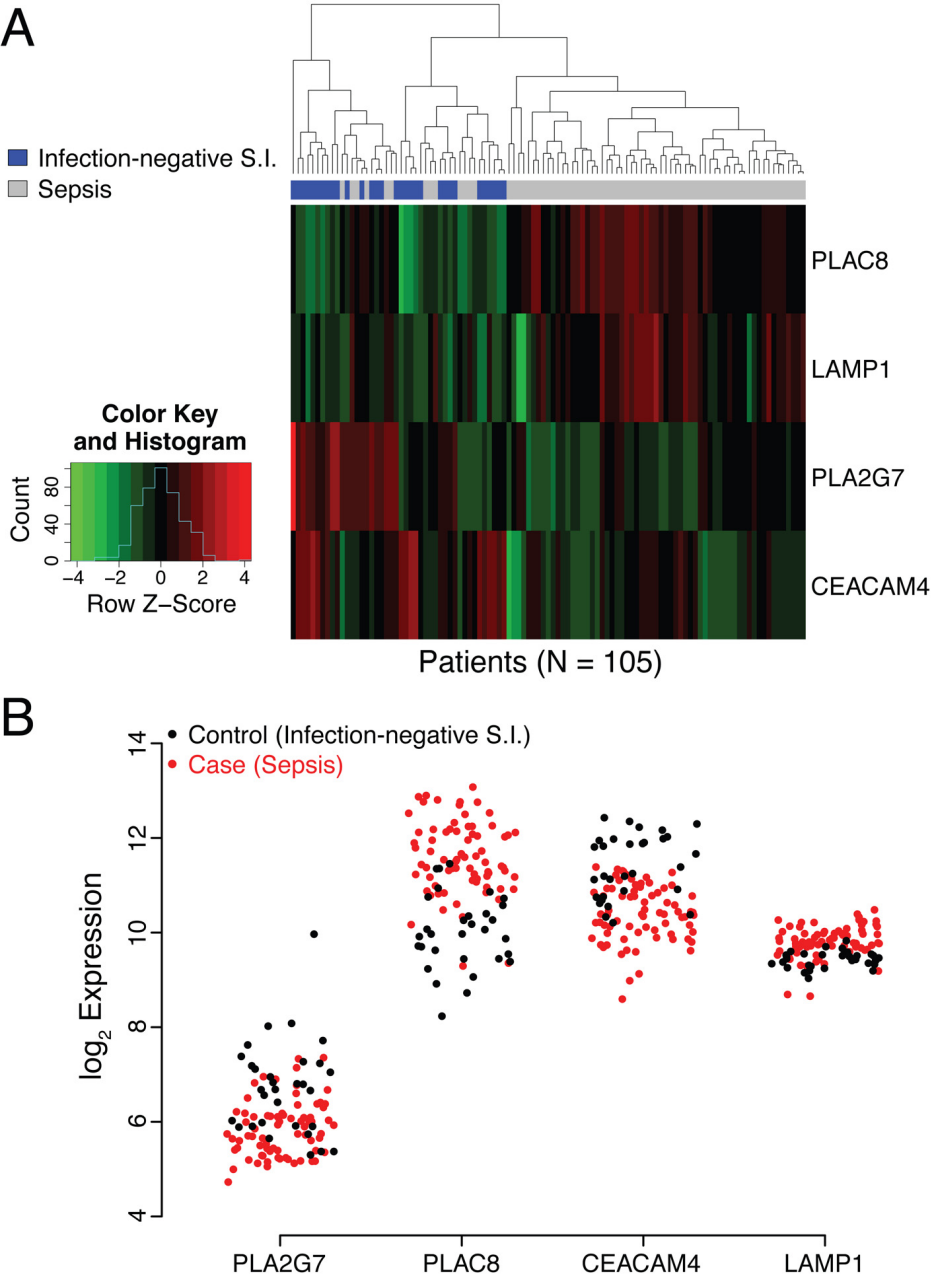
Analysis of behavior of *PLAC8*, *LAMP1*, *PLA2G7*, and *CEACAM4* in the discovery cohort. (A) Heat map representation of the discovery cohort (74 cases, 31 controls). Normalized expression levels of the individual genes comprising the SeptiCyte Lab classifier (color) are plotted versus disease status (dendrogram position) using unsupervised clustering with equally weighted Euclidean distance. The normalization scale (expression level *Z*-score) for up-regulation (red) or down-regulation (green) is shown in the insert at the left of the heat map. (The *Z-*score is the number of standard deviations a value lies away from the mean. Higher absolute *Z-*scores correspond to lower *p-*values. A *Z-*score of ±1.96 equates to a *p-*value of 0.05 in a two-tailed test.) In the cases (sepsis), two genes are predominately up-regulated (*PLAC8* and *LAMP1*), whilst two are predominantly down regulated (*PLA2G7* and *CEACAM4*). (B) Scatterplot representation of microarray expression levels for individual genes in the SeptiCyte Lab classifier, for the discovery cohort. The expression level on log_2_ scale (*y-*axis) is presented for *PLAC8*, *CEACAM4*, *LAMP1*, and *PLA2G7* in individual patients (red for cases, black for controls). Each gene contributes to the ability of the SeptiCyte Lab classifier to separate the cases and controls. S.I., systemic inflammation.

The utility of these four genes in identifying patients with sepsis has not to our knowledge been previously reported. Raw microarray data (log_2_ expression level) for the individual genes are presented as a heat map and dendrogram in Fig 2A. A clear separation of the cases (patients with sepsis) and the controls of the discovery cohort is evident in the dendrogram. This was expected, given the selection process employed, and should not be interpreted as providing evidence for the performance of the SeptiCyte Lab classifier on independent datasets.

Fig 2B presents the raw microarray intensity data for the individual genes. As evident from visual inspection, each gene on its own provides some separation of cases and controls in the discovery cohort. *PLAC8* and *LAMP1* are up-regulated in cases relative to controls, and the opposite behavior is observed for *PLA2G7* and *CEACAM4*. Given the nature of the selection process, it was expected that both up- and down-regulated genes for separating the two groups would be discoverable.

#### Patient stratification

After stratifying by gender, the difference in SeptiCyte Lab AUC between men and women was small and non-significant (*p* = 0.51). Similarly, the difference in AUC according to age (<64 y versus ≥64 y) was found to be small and non-significant (*p* = 0.45).

#### Test with independent microarray dataset

Performance estimates of a classifier generated within, and applied to, a discovery dataset will produce overly optimistic performance expectations. Therefore, performance of the SeptiCyte Lab classifier was assessed on an independent, publicly available microarray dataset (EMBL-EBI dataset E-MTAB-1548). This dataset consisted of PAXgene Blood RNA expression data for 39 cases (post-surgical patients with septic shock) versus 34 controls (patients with systemic inflammatory response syndrome). The SeptiCyte Lab classifier separated the two patient groups in the E-MTAB-1548 dataset with an AUC of 0.89 (95% CI 0.81–0.98) in ROC curve analysis. Additional description of the E-MTAB-1548 dataset and our analysis can be found in S3 Data.

### Validation of the SeptiCyte Lab Classifier

**Fig 3:**
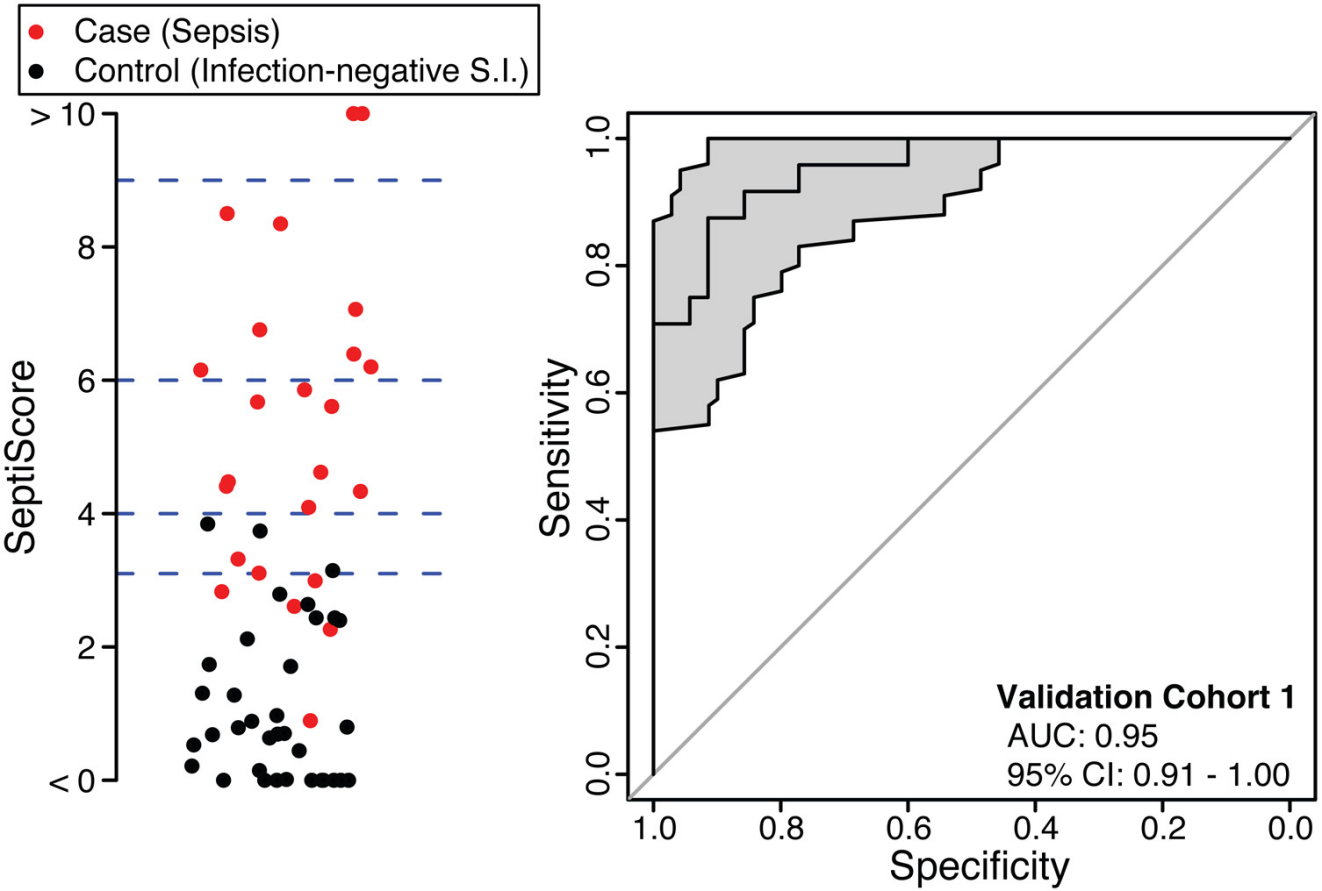
Performance of SeptiCyte Lab in validation cohort 1. Left: Scatterplot of SeptiScores for 24 cases (sepsis; red) versus 35 controls (infection-negative systemic inflammation [S.I.]; black). The blue dashed lines denote SeptiScore values of 3.1, 4.0, 6.0, and 9.0, which are used for subsequent calculations. Right: ROC curve. The grey shading denotes the 95% confidence area.

The SeptiCyte Lab classifier developed in the discovery phase of this study was converted to a RT-qPCR format and used to analyze five additional independent cohorts from a different geographic region (the Netherlands). Patients within validation cohort 1 were selected on the basis of clear-cut, highly confident diagnosis as case or control. ROC curve analysis (Fig 3) gave an AUC of 0.95 (95% CI 0.91–1.00), consistent with the earlier performance of SeptiCyte Lab on microarray data (discovery phase, above).

Following this initial confirmation of SeptiCyte Lab performance, validation cohort 2 was examined. This relatively small, randomly selected cohort was composed of patients sampled across the entire range of available ICU admission dates, and was used mainly to check for bias of SeptiScores with respect to ICU admission date. When the SeptiScores were compared between validation cohorts 1 and 2, no significant differences in frequency distributions (*p* = 0.11 by Welch two-sample *t-*test) or cumulative distributions (*p* = 0.32 by KS test) were found. The results were similar after the six patients with an infection likelihood of possible were removed from cohort 2 (*p* = 0.67 by KS test). These comparisons provided assurance that (1) the initial selection of validation cohort 1 (cases and controls with clear-cut, highly confident diagnoses) was not highly biased toward especially low or high SeptiCyte Lab scores and (2) the date of ICU admission was not a confounding variable. Details of this analysis are provided in S4 Data.

**Fig 4:**
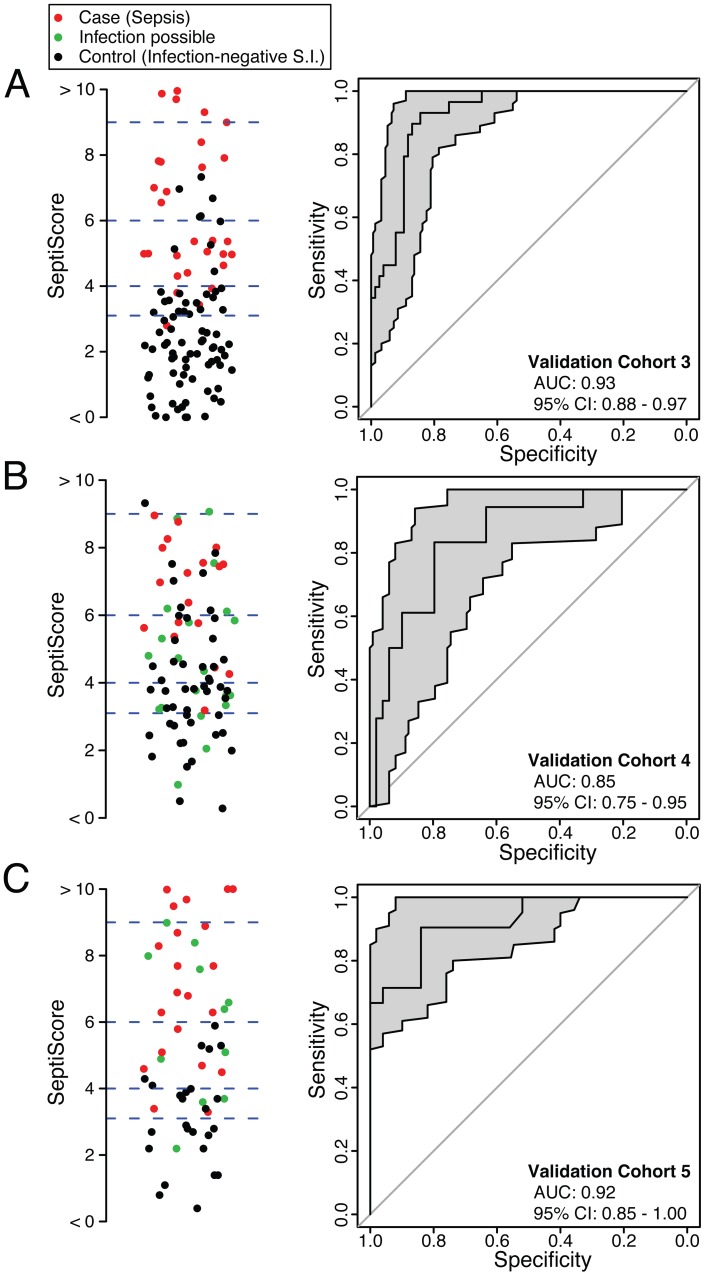
Performance of SeptiCyte Lab in validation cohorts 3, 4, and 5. In each panel, the left side presents a scatterplot of SeptiCyte Lab scores for cases (sepsis; red), controls (infection-negative systemic inflammation [S.I.]; black), and individuals with an infection likelihood of possible (green), and the right side presents the corresponding ROC curve. In the scatterplots, the blue dashed lines denote SeptiScore values of 3.1, 4.0, 6.0, and 9.0, which are used for subsequent calculations. On the right, the grey shading denotes the 95% confidence area for the ROC curve. Patients with an infection likelihood of possible have been excluded from the calculation of ROC curves. (A) Validation cohort 3 (29 cases, 77 controls). (B) Validation cohort 4 (20 cases, 47 controls), consisting of patients sequentially admitted to the ICU. (C) Validation cohort 5 (21 cases, 25 controls), consisting of black and Asian patients sequentially admitted to the ICU.

SeptiCyte Lab was then tested in validation cohorts 3, 4, and 5. These cohorts were chosen to represent the range of patients encountered in the Amsterdam and Utrecht ICUs and approximated sequential admissions. Patients with an infection likelihood of possible (green circles in Fig 4B and 4C) were excluded from the performance calculations. When SeptiCyte Lab was evaluated on validation cohort 3, a ROC curve with AUC = 0.93 (95% CI 0.88–0.97) was obtained (Fig 4A). Analysis of an independent cohort of sequential admissions from the Amsterdam ICU (validation cohort 4, excluding 20 patients with an infection likelihood of possible) produced a ROC curve with AUC = 0.85 (95% CI 0.75–0.95) (Fig 4B). And, finally, in an independent cohort of sequentially enrolled black and Asian patients (validation cohort 5, excluding 11 patients with an infection likelihood of possible), an AUC of 0.92 (95% CI 0.85–1.00) was obtained (Fig 4C). The results of these analyses are summarized in Table 4. By DeLong’s test [36], the AUCs for validation cohorts 3, 4, and 5 did not differ significantly from each other (cohort 3 versus cohort 4: *p* = 0.17; cohort 3 versus cohort 5: *p* = 0.95; cohort 4 versus cohort 5: *p* = 0.24). DeLong’s test also showed no significant difference in AUCs between validation cohorts 3 + 4 (nearly all white) and validation cohort 5 (black + Asian) (*p* = 0.46).

**Table 4:** Diagnostic performance of SeptiCyte Lab in the validation cohorts.

Validation Cohort	*N* Analyzed	*N* Excluded^1^	*N* Controls	*N* Cases	AUC (95% CI)	Accuracy^2^ (95% CI)	Sensitivity^2^ (95% CI)	Specificity^2^ (95% CI)	PPV^2^ (95% CI)	NPV^2^ (95% CI)	LR+^2^ (95% CI)	LR−^2^ (95% CI)
1	59	None	35	24	0.95 (0.91–1.00)	0.86 (0.75–0.94)	0.79 (0.58–0.93)	0.91 (0.77–0.98)	0.86 (0.68–0.95)	0.86 (0.74–0.93)	9.24 (3.07–27.78)	0.23 (0.11–0.50)
2	30	6	27	3	0.77 (0.59–0.94)	0.70 (0.51–0.85)	1.00 (0.29–1.00)	0.67 (0.46–0.84)	0.17 (0.09–0.26)	0.97 (0.88–0.99)	3.00 (1.76–5.11)	0 (0–2.73)
3	106	None	77	29	0.93 (0.88–0.97)	0.76 (0.66–0.83)	0.97 (0.82–1.00)	0.675 (0.56–0.78)	0.53 (0.45–0.61)	0.98 (0.88–1.00)	2.97 (2.14–4.13)	0.051 (0.007–0.35)
4	67	20	49	18	0.85 (0.75–0.95)	0.51 (0.38–0.63)	1.00 (0.82–1.00)	0.33 (0.20–0.48)	0.27 (0.22–0.31)	0.95 (0.78–0.98)	1.48 (1.22–1.80)	0 (0–1.31)
5 (black + Asian)	46	11	25	21	0.92 (0.85–1.00)	0.74 (0.59–0.86)	1.00 (0.84–1.00)	0.52 (0.31–0.72)	0.53 (0.43–0.62)	0.94 (0.74–0.98)	2.08 (1.38–3.13)	0 (0–0.71)
1 + 2 + 3 + 4	262	26	188	74	0.87 (0.82–0.91)	0.71 (0.65–0.76)	0.92 (0.83–0.97)	0.63 (0.55–0.70)	0.46 (0.41–0.51)	0.96 (0.91–0.98)	2.47 (2.03–3.01)	0.13 (0.060–0.28)
2 + 3 + 4 + 5	249	37	178	71	0.89 (0.85–0.93)	0.68 (0.62–0.74)	0.99 (0.92–1.00)	0.56 (0.48–0.63)	0.42 (0.38–0.46)	0.99 (0.94–1.00)	2.22 (1.88–2.62)	0.025 (0.004–0.18)
1 + 2 + 3 + 4 + 5	308	37	213	95	0.88 (0.84–0.92)	0.71 (0.66–0.76)	0.94 (0.87–0.98)	0.62 (0.55–0.68)	0.48 (0.44–0.52)	0.96 (0.92–0.98)	2.43 (2.04–2.91)	0.10 (0.047–0.24)
1 + 2 + 3 + 4 + 5, male only^3^	167	21	116	51	0.86 (0.81–0.92)	0.70 (0.62–0.77)	0.94 (0.84–0.99)	0.60 (0.50–0.68)	0.46 (0.41–0.52)	0.96 (0.90–0.99)	2.32 (1.84–2.93)	0.099 (0.032–0.30)
1 + 2 + 3 + 4 + 5, female only^3^	141	15	97	44	0.90 (0.84–0.95)	0.73 (0.65–0.80)	0.93 (0.81–0.99)	0.64 (0.54–0.73)	0.51 (0.44–0.57)	0.96 (0.89–0.99)	2.58 (1.96–3.41)	0.11 (0.035–0.32)
1 + 2 + 3 + 4 + 5, <64 y	182	20	125	57	0.89 (0.84–0.94)	0.72 (0.65–0.78)	0.95 (0.85–0.99)	0.62 (0.54–0.70)	0.49 (0.44–0.55)	0.97 (0.91–0.99)	2.47 (1.96–3.11)	0.085 (0.028–0.26)
1 + 2 + 3 + 4 + 5, ≥64 y	126	17	88	38	0.87 (0.80–0.93)	0.71 (0.62–0.78)	0.92 (0.79–0.93)	0.61 (0.50–0.72)	0.46 (0.40–0.53)	0.96 (0.88–0.98)	2.38 (1.80–3.15)	0.13 (0.043–0.39)

^1^Patients with an infection likelihood of possible were excluded from performance analysis.

^2^For calculation of accuracy, sensitivity, specificity, PPV, NPV, LR+, and LR−, a binary cutoff of 3.100 for the SeptiScore was assumed.

^3^Sex was not recorded for one patient.

Besides testing SeptiCyte Lab performance on validation cohort 5, which consisted of black and Asian patients, additional tests for the robustness of SeptiCyte Lab were performed by stratifying the entire dataset (*n* = 308, excluding 37 patients with an infection likelihood of possible) on gender [64,65] or age (<64 versus ≥64 y) [66–69]. By DeLong’s test [36], the AUCs for these strata did not show significant differences (female versus male: *p* = 0.52; age < 64 y versus age ≥ 64 y: *p* = 0.70). Thus, SeptiCyte Lab was able to differentiate cases from controls across both genders and a range of ages with high accuracy (AUC = 0.9).

In comparing SeptiCyte Lab performance between different subsets of samples from the validation cohorts, we observed no significant differences in performance between validation cohorts 3, 4, and 5 or between genders or age groups (age < 64 y versus age ≥ 64 y). DeLong’s test [36] was used to estimate the significance (*p*-value) of each pairwise comparison, and this test takes into account sample size. Each of the comparisons involved a reasonably large number of samples (106 for cohort 3, 67 for cohort 4, 46 for cohort 5, 167 for male, 141 for female, 182 for age < 64 y, 126 for age ≥ 64 y), and no significant differences in performance were observed below the *p* = 0.10 level. However, we note that our validation studies were a priori designed and powered to evaluate population-level performance of SeptiCyte Lab, and not for the purpose of comparing different strata. Thus, in stratification, the sample numbers in the comparison groups may have been decreased to levels for which small performance differences might no longer be detectable. We have not performed a formal sample size determination to establish the smallest differences that might reasonably be detected in our pairwise comparisons.

A ROC curve describes the performance of SeptiCyte Lab independently of assigning a binary cutoff for the SeptiScore. Alternatively, a binary cutoff may be assigned to discriminate between cases and controls, and then performance parameters such as accuracy, sensitivity, specificity, PPV, NPV, LR+, LR−, NRI+, and NRI− can be calculated. We assigned a binary cutoff of 3.100 (which favors sensitivity at the expense of specificity) and calculated these measures of performance for the validation cohorts, and for various combinations and stratifications thereof. The results are summarized in Table 4.

### Examination of Disease Severity as a Potential Confounding Variable

**Fig 5:**
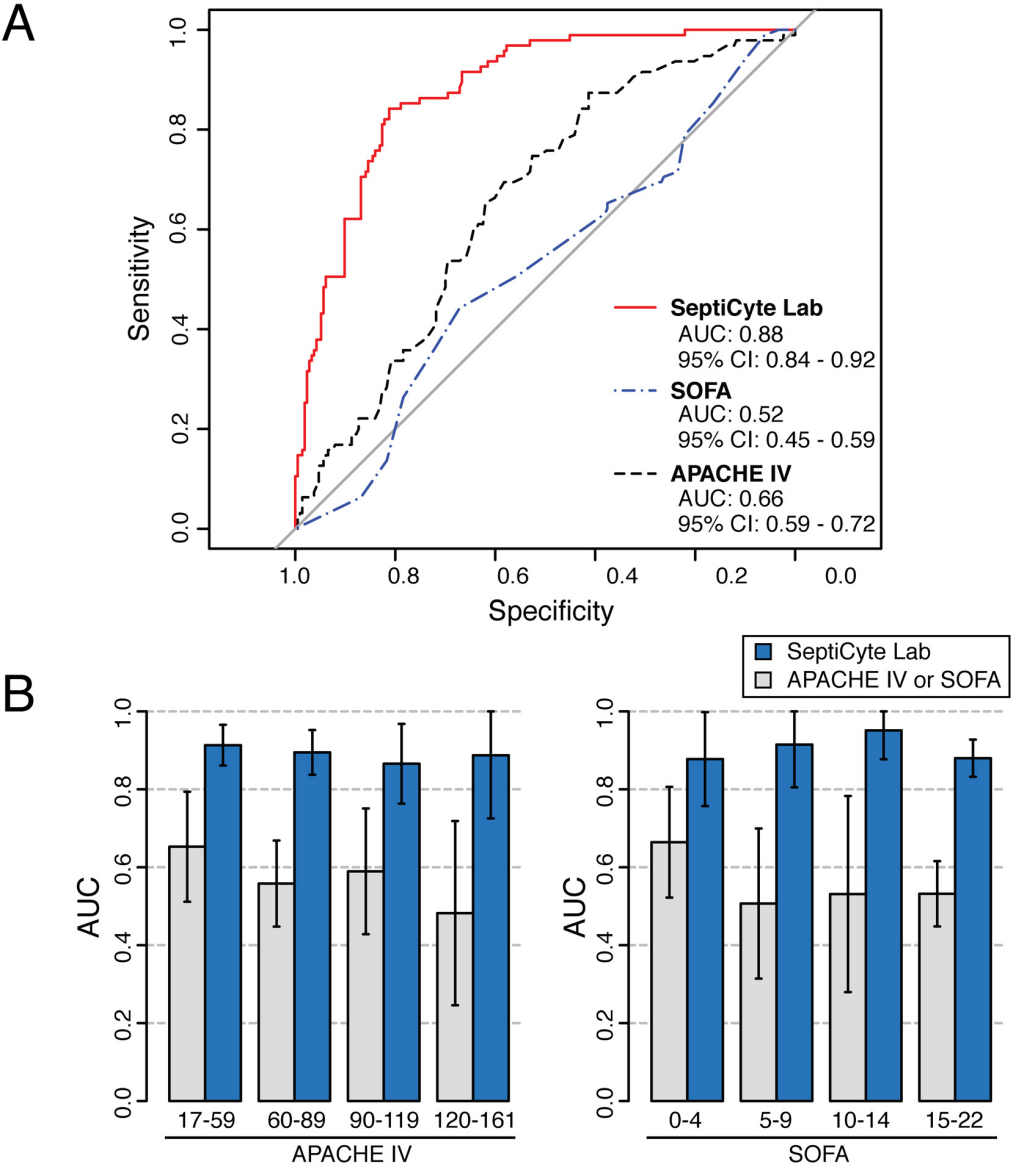
Test for disease severity as a potential confounding variable. Validation cohorts 1–5 (excluding patients with an infection likelihood of possible) were combined, and then stratified on APACHE IV score or SOFA score. (A) ROC curve analysis was performed on the combined dataset. (B) Separate ROC curve analyses were performed over sub-ranges of the APACHE IV score (left) or SOFA score (right). The error bars indicate the 95% CI for the AUC as estimated by resampling. The results for the various strata of APACHE IV and SOFA score indicate that disease severity has a relatively weak influence (AUC = 0.52–0.65) on the discrimination of cases from controls, while SeptiCyte Lab has a much stronger influence (AUC = 0.87–0.91). Additional details of the calculations are given in S5 Data.

Severity of disease could be a confounding variable in using SeptiCyte Lab to discriminate cases from controls. To address this concern, a ROC curve analysis on the entire patient pool (*n* = 308, excluding 37 patients with an infection likelihood of possible) was conducted using either the Sequential Organ Failure Assessment (SOFA) score or the Acute Physiology and Chronic Health Evaluation (APACHE) IV score as a classifier. This analysis, summarized in Fig 5A, revealed only a weak discrimination for these classifiers (AUC = 0.66 for APACHE IV; AUC = 0.52 for SOFA), in contrast to the strong discrimination (AUC = 0.88) achieved by SeptiCyte Lab.

Additionally, each stratum of patients, characterized by a range of APACHE IV or SOFA scores, was analyzed to determine whether the performance of SeptiCyte Lab was stratum-dependent. Comparison of the ROC curves from individual strata revealed no significant differences (*p* > 0.23 for each APACHE IV comparison; *p* > 0.30 for each SOFA comparison). The results of this secondary analysis are summarized in Fig 5B and presented in greater detail in S5 Data. Thus, with respect to discrimination of cases from controls by SeptiCyte Lab, any confounding effect of disease severity, as measured by APACHE IV or SOFA score, appeared small.

### The Challenge of Diagnosing Patients with an Infection Likelihood of Possible

To estimate the diagnostic performance of SeptiCyte Lab using ROC curve analysis, we first removed patients having an infection likelihood of possible. These were patients for whom an assignment of infection likelihood—and therefore classification as either case or control—could not be made with high confidence. However, by excluding these patients, spectrum bias may be introduced into estimates of performance [70–73]. To address this concern, a KS test was used to determine whether the statistical distribution of SeptiScores was different for patients with an infection likelihood of possible, as compared to patients with an unambiguous classification. The analysis was based on the entire available dataset (*n* = 345) and compared all 37 patients with an infection likelihood of possible to the remaining 308 patients with known disease status. No significant difference was found (*p* = 0.37) between the cumulative distributions of the SeptiScore for these two classes of patients (see S6 Data). Thus, if only the SeptiScore is considered, patients with an infection likelihood of possible are indistinguishable from patients of known disease status.

### Comparative Performance of SeptiCyte Lab

**Fig 6:**
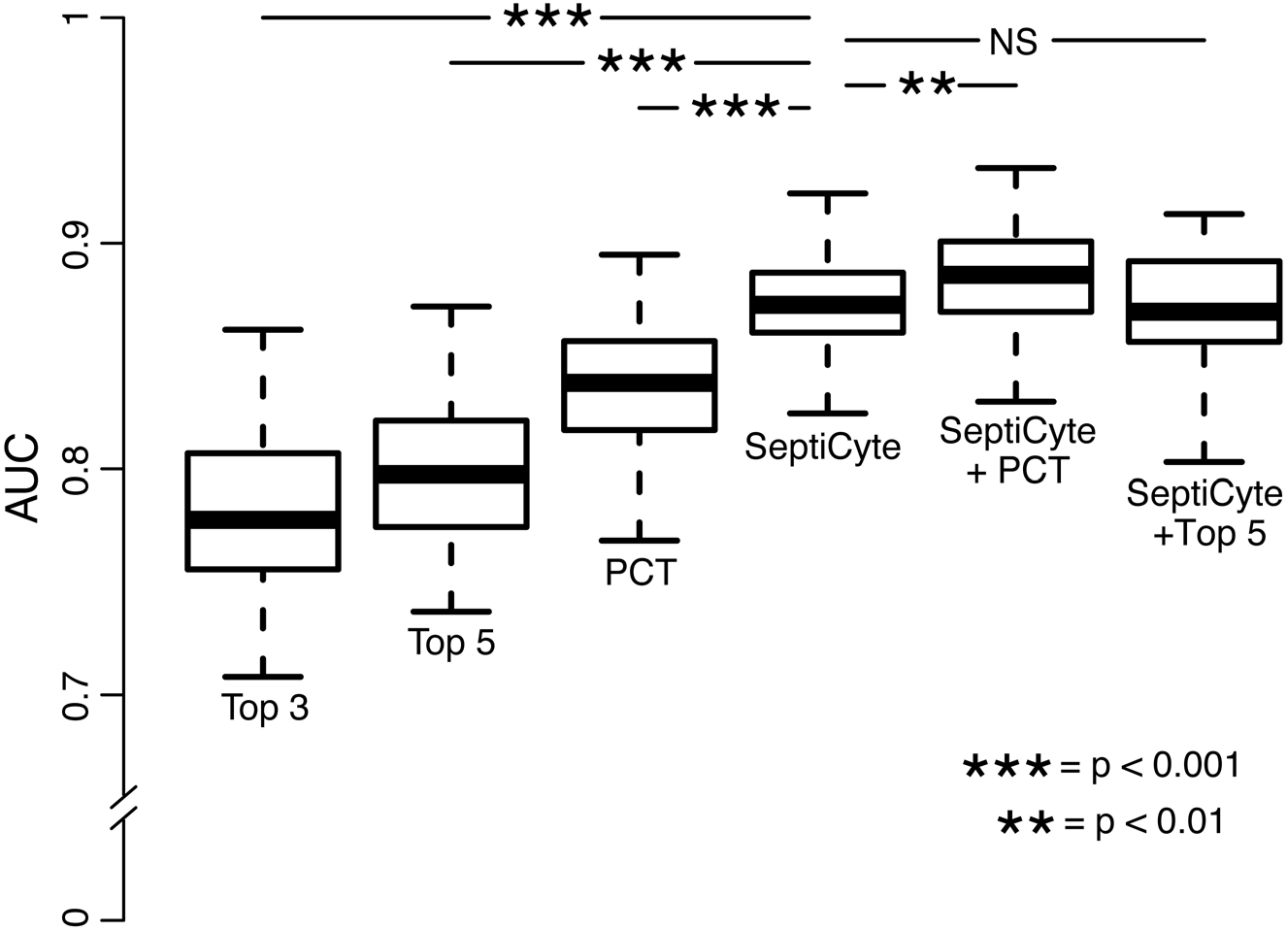
Performance of PCT, clinical parameters, and SeptiCyte Lab for the discrimination of sepsis versus infection-negative systemic inflammation. This comparative analysis used the largest set of patients (*n* = 157) for which values were available for all comparisons. Patients having an infection likelihood of possible were excluded. Performance is reported as AUC. The error bars indicate the 95% CI for the AUC, obtained from a 50 × 2 cross-validation with training and test samples selected in a 1:1 ratio. The significance levels (*p*-value by *t-*test) for pairwise comparisons to SeptiCyte Lab are indicated in the figure. Details of the analysis are given in S7 Data. NS, not significant; Top 3, top three clinical parameters; Top 5, top five clinical parameters.

SeptiCyte Lab, PCT, C-reactive protein (CRP), and various clinical parameters (individually and in combination) were compared with respect to their ability to discriminate cases from controls. The choice of clinical parameters was restricted to those that are readily available within 24 h of ICU admission and that might serve as the basis for a sepsis diagnosis [23]. Results are summarized in Table 5 and Fig 6, and presented in detail in S7 Data.

**Table 5:** Diagnostic performance of SeptiCyte Lab versus PCT.

Validation Cohort	Number of Patients That Could Be Analyzed^1^	Parameter	Value^2^		*p-*Value
			PCT (95% CI)	SeptiCyte Lab (95% CI)	
1 (*n* = 59)	50	AUC	0.80 (0.67, 0.93)	0.96 (0.91, 1.00)	0.013
		Accuracy	0.74 (0.60, 0.85)	0.78 (0.64, 0.88)	0.64
		Sensitivity	0.58 (0.34, 0.80)	0.95 (0.74, 1.00)	0.12
		Specificity	0.84 (0.66, 0.94)	0.68 (0.49, 0.83)	0.27
		PPV	0.69 (0.48, 0.84)	0.64 (0.52, 0.75)	0.71
		NPV	0.76 (0.65, 0.85)	0.96 (0.75, 0.99)	0.0071
		LR+	3.59 (1.48, 8.74)	2.94 (1.74, 4.94)	0.71
		LR−	0.50 (0.29, 0.87)	0.078 (0.011, 0.53)	0.049
		NRI+		0.37 (0.15, 0.58)	<0.001
		NRI−		−0.16 (−0.38, 0.06)	0.15
3 (*n* = 106)	97	AUC	0.83 (0.74, 0.92)	0.93 (0.88, 0.98)	0.028
		Accuracy	0.75 (0.66, 0.84)	0.42 (0.32, 0.53)	<0.001
		Sensitivity	0.67 (0.46, 0.84)	1.00 (0.87, 1.00)	0.039
		Specificity	0.79 (0.67, 0.88)	0.20 (0.11, 0.31)	<0.001
		PPV	0.54 (0.42, 0.67)	0.32 (0.28, 0.35)	0.0012
		NPV	0.86 (0.78, 0.91)	0.92 (0.68, 0.97)	0.0048
		LR+	3.11 (1.85, 5.24)	1.25 (1.11, 1.40)	NC^3^
		LR−	0.42 (0.24, 0.73)	0 (0.00, 1.47)	NC^3^
		NRI+		0.33 (0.16, 0.51)	<0.001
		NRI−		−0.59 (−0.71, −0.46)	<0.001
5 (*n* = 46)	42	AUC	0.75 (0.60, 0.90)	0.92 (0.83, 1.00)	0.031
		Accuracy	0.60 (0.43, 0.75)	0.52 (0.36, 0.68)	0.38
		Sensitivity	0.75 (0.51, 0.91)	1.00 (0.83, 1.00)	0.38
		Specificity	0.50 (0.28, 0.72)	0.091 (0.011, 0.29)	0.039
		PPV	0.58 (0.46, 0.69)	0.50 (0.45, 0.55)	0.19
		NPV	0.69 (0.48, 0.84)	0.72 (0.29, 0.91)	0.13
		LR+	1.50 (0.920, 2.445)	1.10 (0.96, 1.26)	NC^3^
		LR−	0.50 (0.21, 1.19)	0 (0.00, 5.60)	NC^3^
		NRI+		0.25 (0.06, 0.44)	0.0098
		NRI−		−0.41 (−0.61, −0.20)	<0.001
1 + 3 + 5 (*n* = 211)	189	AUC	0.81 (0.75, 0.87)	0.92 (0.88, 0.96)	<0.001
		Accuracy	0.72 (0.65, 0.78)	0.54 (0.47, 0.61)	<0.001
		Sensitivity	0.67 (0.54, 0.78)	0.98 (0.92, 1.00)	<0.001
		Specificity	0.75 (0.66, 0.82)	0.30 (0.22, 0.39)	<0.001
		PPV	0.59 (0.50, 0.67)	0.43 (0.40, 0.46)	<0.001
		NPV	0.81 (0.74, 0.86)	0.97 (0.84, 1.00)	<0.001
		LR+	2.64 (1.87, 3.75)	1.41 (1.25, 1.59)	<0.001
		LR−	0.45 (0.31, 0.64)	0.050 (0.007, 0.36)	0.027
		NRI+		0.32 (0.21, 0.43)	<0.001
		NRI−		−0.45 (−0.55, −0.34)	<0.001

^1^Patients could be analyzed if classified as cases or controls (i.e., if not assigned an infection likelihood of possible) and if PCT results were available.

^2^A SeptiCyte Score cutoff of 3.100 and a PCT cutoff of 2 ng/ml were used to calculate accuracy, sensitivity, specificity, PPV, NPV, LR+, LR−, NRI+, and NRI−.

^3^Not calculable, because the algorithm failed to converge in the logistic regression model as described by Gu and Pepe [41] and as implemented in DTComPair version 1.00 [44]. Therefore it is not possible to estimate *p*-value.

The performance of SeptiCyte Lab was benchmarked to that of PCT. A total of 189 patients with PCT data and an unambiguous diagnosis as either case or control were available for analysis in validation cohorts 1, 3, and 5. AUCs produced by SeptiCyte Lab were consistently 0.10–0.17 higher than AUCs for PCT in each cohort or combination of cohorts analyzed; all differences were statistically significant at *p ≤* 0.03 (Table 5). For the combined validation cohorts 1 + 3 + 5, using retrospective physician assessment as the standard and excluding patients with an infection likelihood of possible, the reported AUC for SeptiCyte Lab was 0.92 and the reported AUC for PCT was 0.81, indicating that PCT had approximately 2.4 times the error rate of SeptiCyte Lab (19% as opposed to 8%) in distinguishing cases from controls. The performance of SeptiCyte Lab and PCT was compared not only by AUC but also by the following measures: accuracy, sensitivity, specificity, PPV, NPV, LR+, LR−, NRI+, and NRI−, as summarized in Table 5. A SeptiCyte Lab cutoff of 3.100 and a PCT cutoff of 2 ng/ml were used in calculating these performance measures.

CRP performed conspicuously well in discriminating cases from controls in our cohorts (AUC = 0.84–0.86; 95% CI 0.78–0.95; see S7 Data). This was surprising in light of literature reports indicating that CRP is expected to perform with an AUC of 0.7 to 0.8 in distinguishing between these two conditions [74–76]. We note that only a limited number of patients in our validation cohorts (173/345 = 50.1%) had CRP measurements. We note also that the attending physicians took the CRP values into consideration when retrospectively assessing infection likelihood. Thus, a selection bias might underlie the anomalously high AUC observed for CRP.

Fig 6 presents the AUCs for discrimination of cases from controls, using the top three clinical parameters, the top five clinical parameters, PCT, SeptiCyte Lab, SeptiCyte Lab + PCT, and SeptiCyte Lab + the top five clinical parameters. The most effective combination of five clinical parameters was found to be the following: minimum PaO_2_/FIO_2_ ratio within 24 h of ICU admission, maximum bilirubin within 24 h of ICU admission, total urine output within 24 h of ICU admission, glucose concentration, and maximum heart rate within 24 h of ICU admission.

When the *n* = 157 patient dataset held in common by all classifiers was analyzed, SeptiCyte Lab (AUC = 0.88; 95% CI 0.81–0.93) was found to outperform both PCT (AUC = 0.84; 95% CI 0.76–0.92; *p <* 0.001) and the best combination of five clinical parameters (mean AUC = 0.81; 95% CI 0.71–0.89; *p <* 0.001) for discriminating cases from controls. When SeptiCyte Lab was added to PCT, an increase in AUC was observed (AUC = 0.89; 95% CI 0.82–0.95). Also, when SeptiCyte Lab was added to the best combination of five clinical parameters, an increase in AUC was observed (AUC = 0.87; 95% CI 0.79–0.93). When PCT was added to SeptiCyte Lab, a small but significant (*p <* 0.01) increase in mean AUC is observed (AUC = 0.89; 95% CI 0.82–0.95). However, no significant increase in AUC is observed when the top five clinical parameters were added to SeptiCyte Lab (AUC = 0.87; 95% CI 0.79–0.93). We note that attending physicians in the ICU considered all available clinical parameters in assessing infection likelihood. Because the clinical parameters used in the regression modeling were also used in the retrospective physician assessment of infection likelihood, the clinical parameter combinations examined here are expected to have inflated AUCs. Thus, the true (unbiased) performance of clinical parameter combinations is likely to be lower than reported here.

### Negative Predictive Value of SeptiCyte Lab

Our largest available dataset consisted of the combined validation cohorts 1 + 2 + 3 + 4 + 5 (*n* = 308, comprising 95 cases and 213 controls and excluding 37 patients with an infection likelihood of possible). From the cumulative distributions of the SeptiScore for the cases and controls, we calculated likelihood ratios for different ranges of the SeptiScore, as indicated in Table 6. The prevalence of retrospectively diagnosed sepsis in this combined cohort was 31%, which is consistent with the reported value of 30% sepsis in Dutch ICUs [77]. We equated this observed 30% sepsis prevalence to a pre-test sepsis probability of 30%. For patients with SeptiScore < 4, we calculated the post-test probability of sepsis to be 1.2%, which corresponds to a NPV of 98.8%. We note that 29% of all patients in this dataset had SeptiScore < 4, suggesting that these patients may have been treated unnecessarily with antibiotics.

**Table 6:** SeptiScores, likelihood ratios, and disease probabilities.

SeptiScore	*N* Controls	*N* Sepsis Cases	Percent of All Patients	Percent of Controls	Percent of Cases	Control:Case Ratio	Likelihood Ratio	Pre-Test Probability^1^	Post-Test Probability
<4	81	1	28.9	38.0	1.2	32:1	0.028	30%	1.2%
4 to 6	88	14	33.1	41.3	13.7	3:1	0.36	30%	16%
6 to 9	35	45	26.0	16.4	56.3	1:3.4	2.88	30%	55%
>9	9	35	14.3	4.2	79.5	1:19	8.72	30%	79%

Calculations were performed for the combined validation cohorts 1 + 2 + 3 + 4 + 5 (*n* = 308, 95 cases, 213 controls, with the exclusion of 37 patients having an infection likelihood of possible).

^1^Pre-test probability was assumed to equal the percent of patients with sepsis in the set of all patients studied.

## Discussion

The present study achieved four objectives: (1) identification of a classifier (SeptiCyte Lab) to accurately discriminate cases (patients with retrospectively diagnosed sepsis) from controls (patients with infection-negative systemic inflammation), (2) conversion of the classifier from a microarray format to RT-qPCR format, (3) validation of the classifier performance in independent patient cohorts, and (4) demonstration of diagnostic utility by showing that the performance of the classifier in the studied patient cohorts is superior to that of PCT and various combinations of clinical parameters. Although the genes underlying the SeptiCyte Lab classifier (*PLAC8*, *PLA2G7*, *LAMP1*, and *CEACAM4*) are known to be involved in innate immunity and the host response to infection (see Table 3), their utility in discriminating sepsis from infection-negative systemic inflammation has not to our knowledge been previously reported.

### Clinical Utility

Early and accurate detection of sepsis, followed by appropriate therapeutic intervention, is critical for reducing patient morbidity and mortality. By the time sepsis reaches the advanced stage of septic shock, the nature of the problem is clear, but therapeutic intervention may be dangerously late. In the cohorts tested, we found that SeptiCyte Lab could distinguish cases from controls with a diagnostic accuracy approaching AUC = 0.9 within several hours of the first suspicion of sepsis. When the test was run in binary mode with an appropriate cutoff, a high negative predictive value (95%) was obtained. In our cohorts, SeptiCyte Lab outperformed PCT (currently the only protein biomarker of sepsis cleared by the US Food and Drug Administration [FDA]; FDA 510k number: K040887). The test retained high performance in patients showing few signs of organ dysfunction (i.e., low APACHE IV or SOFA score), and thus appeared unlinked to sepsis severity and able to diagnose sepsis early in the absence of multiple clinical signs.

Ultimately, the general clinical utility of SeptiCyte Lab will be evaluated through multiple validation studies in a variety of clinical settings, which will include patients with less definitive sepsis diagnoses [78]. If appropriately validated in further clinical cohorts, the information delivered by SeptiCyte Lab, in conjunction with available clinical parameters, may provide the physician with the ability not only to recognize sepsis in its early stages, but also to implement a targeted early treatment regime. It is expected that early, goal-directed therapy will help prevent or minimize progression to multi-organ dysfunction. Low SeptiScores, which in our cohorts correlated with low sepsis probability, could also provide physicians with an objective basis for reducing or eliminating antibiotic treatment for patients who display “sterile” systemic inflammation.

The samples analyzed in this study were collected in PAXgene Blood RNA tubes (PreAnalytiX/Becton Dickinson). RNA extraction was performed using a protocol cleared by the FDA for diagnostic use of RNA in transcript expression profiling from blood (FDA 510k number: K082150). Using the PAXgene Blood RNA tube and extraction procedure, the turnaround time of the SeptiCyte Lab assay (sample to result) is approximately 4 to 6 h. Early development efforts to port the SeptiCyte Lab assay to a point-of-care platform are underway. The point-of-care version will use predispensed reagents, require less operator training, and have a targeted turnaround time of approximately 1.5 h.

### Strengths and Limitations of the Study

We consider the present study to have a number of strengths over previously published work on multiplexed biomarkers for sepsis diagnosis, including our earlier work [18–22]. First, we have demonstrated the robustness of the SeptiCyte Lab classifier across gender, race, age, and date of ICU admission. No statistically significant differences in SeptiScore distributions or performance (as measured by AUC) were observed in any of the pairwise cohort comparisons, indicating that SeptiCyte Lab performance is robust across the most common sources of diversity amongst patients presenting in clinical practice. Second, we have demonstrated specificity by showing that the diagnostic performance of SeptiCyte Lab is uninfluenced by disease severity as measured by APACHE IV or SOFA score [79–81], two widely used measures of organ failure, disease severity, and prognosis in ICU settings. Thus, disease severity, as measured by organ dysfunction scores, is not a major confounding variable with respect to SeptiCyte Lab performance. Third, we have shown the diagnostic utility of SeptiCyte Lab by comparing its performance to that of PCT and various combinations of clinical and laboratory parameters that would be available to an attending physician attempting to diagnose sepsis in a patient within 24 h of ICU admission [23]. SeptiCyte Lab’s performance was statistically superior to that of PCT for the individual validation cohorts 1, 3, and 5, and also for the combined validation cohorts 1 + 3 + 5, consistently having a 0.09–0.17 higher AUC than that of PCT. We also found that SeptiCyte Lab outperformed the most effective combination of five clinical parameters and that the highest AUC was obtained when combining these five parameters with SeptiCyte Lab. Thus, SeptiCyte Lab appears to be diagnostically superior to PCT and appears to provide diagnostic information beyond that provided by the best combination of sepsis-related clinical parameters.

We argued above that the accuracy, robustness, and fast turnaround time of SeptiCyte Lab will have clinical utility for physicians attempting to rapidly diagnose sepsis and make appropriate therapeutic choices. However, because sepsis has a high risk of morbidity and mortality, attending ICU physicians tend to prescribe antibiotics in cases for which there is simply a suspicion of sepsis [82]. A test to discriminate sepsis from infection-negative systemic inflammation would need to lower the post-test probability of sepsis to a very low value (~1%) to be consistent with an experienced physician’s decision to withhold antibiotics from a patient suspected of sepsis. Using the reported sepsis prevalence of ~30% in Dutch ICUs [60] and a SeptiScore less than 4, our calculations using the results from our cohorts show a NPV of 98.8%, which, if validated in a population-based cohort, may be sufficient for a clinician to withhold antibiotics, at least until follow-up diagnostic results are available.

In practice, the SeptiCyte Lab result would be evaluated in conjunction with other clinical signs and symptoms, and not as a standalone result. Because the probability of an infection-negative state is greater at low SeptiScores, the probability of sepsis is greater at high SeptiScores, and there is a continuous gradation between the two extremes, the test output is best reported as a likelihood ratio instead of a binary call. However, a clinician must make a binary decision of whether or not to treat a patient with antibiotics; an asymmetric risk profile applies to this decision. The consequence of a false negative call (in which a true sepsis case is missed and antibiotics withheld) is greater than that of a false positive call (in which an infection-negative state is called sepsis, and antibiotics given unnecessarily). Therefore, a binary cutoff should be set at a value that decreases the risk of false negatives to an acceptable level. If SeptiCyte Lab were used for other purposes, such as cohort selection in a clinical trial, different considerations would apply, and a binary cutoff might be set differently.

The present study has limitations related to the composition of the discovery cohort: the cases consisted exclusively of microbiologically and/or clinically confirmed sepsis, and the controls consisted exclusively of patients with infection-negative systemic inflammation due to elective invasive surgery for non-infection-related conditions. Thus, both cases and controls had a narrower clinical range of characteristics than would be expected in practice. However, such a limitation is ameliorated by the extensive validation testing of SeptiCyte Lab that was subsequently performed on multiple independent cohorts from the Amsterdam (Academic Medical Center) and Utrecht (University Medical Center Utrecht) ICU sites.

Another limitation relates to the composition of the validation cohorts. For example, validation cohort 2 (used mainly to check whether ICU admission date was a confounding variable) was smaller and less balanced than would be ideal (*n* = 36, with only three cases). Also, validation cohort 4 (sequential patients admitted to the Amsterdam ICU) consisted entirely of white patients (except possibly one patient for whom race was not recorded). In contrast, the racial makeup of the Netherlands includes approximately 10% non-white minorities. The lack of inclusion of non-white minorities in validation cohort 4 can be ascribed to random sampling variation due to limited cohort size. We addressed the issue of racial bias by examining validation cohort 5, which consisted of black and Asian patients sequentially admitted to the Amsterdam and Utrecht ICUs. SeptiCyte Lab performance was maintained in validation cohort 5. Overall, we believe the 345 patients of validation cohorts 1–5 compose a representative sampling of ICU admissions encountered in practice.

A third limitation is that a complete, unbiased performance comparison to CRP could not be made. Only 173/345 (50.1%) patients in the validation cohorts had CRP measurements. Furthermore, the attending physicians considered these measurements in assessing infection likelihood, thus introducing the possibility of selection bias.

Finally, all historical and current attempts to differentiate sepsis from infection-negative systemic inflammation are complicated by the fact that a true “gold standard” does not exist. Thus, patients with an infection likelihood of possible cannot be classified as either cases or controls with high confidence by any reference method currently in use. From the descriptions of validation cohorts 2, 3, 4, and 5 provided in the Results, we obtain the following point estimates for the frequency of patients having an infection likelihood of possible in the intended use population: 6/36 = 17% from cohort 2 data, 91/775 = 12% from cohort 3 data, 20/87 = 23% from cohort 4 data, 11/57 = 19% from cohort 5 data, and 128/955 = 13.4% from the data of cohorts 2 + 3 + 4 + 5 combined. The deliberate exclusion of patients with an infection likelihood of possible from our performance analyses introduces the possibility of spectrum bias [70–73]. The KS test was used to address this concern by determining whether the SeptiScore had a different statistical distribution for patients with an infection likelihood of possible, as compared to patients with an unambiguous classification by the reference method. No significant difference was observed between the cumulative distributions of the SeptiScore, which argues against the introduction of spectrum bias.

We argue that, during the development of a new diagnostic assay for sepsis, the deliberate exclusion of patients with an infection likelihood of possible is necessitated by five factors: (1) the definition of sepsis was originally coined to “provide a conceptual and practical framework” rather than to provide “a clinically useful set of criteria for diagnosing sepsis and related conditions” [23,83]; (2) there is currently no gold standard diagnostic test for sepsis [3,7]; (3) any tissue injury resulting in systemic inflammation often has some microbial involvement [84,85]; (4) clinical signs of sepsis (and suspected sepsis) are time-course-dependent, and therefore timing of diagnosis is important; and (5) currently, a diagnosis of sepsis ultimately rests with the attending physician. These factors will constrain the development and validation of any test that attempts to distinguish sepsis from infection-negative systemic inflammation.

### Conclusions

SeptiCyte Lab, a peripheral blood-based molecular assay, has been shown to be rapid, robust, and accurate for differentiating cases (ICU patients retrospectively diagnosed with sepsis) from controls (ICU patients retrospectively diagnosed with infection-negative systemic inflammation). In combination with clinical parameters and clinical judgment, SeptiCyte Lab may provide physicians with enhanced confidence in therapeutic decision-making for patients with systemic inflammation. Further clinical studies are required to confirm these findings.

## Supporting Information

S1 Data Translation between microarray and RT-qPCR formats. (PDF)

S2 Data Line data files. (XLSX)

S3 Data Test of SeptiCyte Lab classifier against an independent microarray dataset. (PDF)

S4 Data ICU admission date as a potential confounding variable. (PDF)

S5 Data Disease severity as a potential confounding variable. (PDF)

S6 Data Analysis of patients with infection likelihood of possible. (PDF)

S7 Data Multi-parameter analysis. (PDF)

S1 Text Summaries of the GCP-1 and RTT study protocols (discovery phase) and the MARS study (validation phase). (PDF)

S2 Text Definition and selection of validation cohorts. (PDF)

S3 Text STARD checklist. (PDF)

## Abbreviations

ABI: Applied Biosystems
APACHE: Acute Physiology and Chronic Health Evaluation
APT: Affymetrix Power Tools
AUC: area under curve
CI: confidence interval
CRP: C-reactive protein
FDA: US Food and Drug Administration
HREC: Human Research Ethics Committee
ICU: intensive care unit
KS: Kolmogorov-Smirnov
LR+: positive likelihood ratio
LR−: negative likelihood ratio
MARS: Molecular Diagnosis and Risk Stratification of Sepsis
NPV: negative predictive value
NRI+: net reclassification index for positive samples
NRI−: net reclassification index for negative samples
PCT: procalcitonin
PPV: positive predictive value
ROC: receiver operating characteristic
RT-qPCR: reverse transcription quantitative polymerase chain reaction
SOFA: Sequential Organ Failure Assessment
