# Supplementary material for: A Molecular Host Response Assay to Discriminate Between Sepsis and Infection-Negative Systemic Inflammation in Critically Ill Patients: Discovery and Validation in Independent Cohorts
Source: PLoS Med. 2015 Dec 8;12(12):e1001916. doi: 10.1371/journal.pmed.1001916 (PMC4672921; doi:10.1371/journal.pmed.1001916)
Supplement: S1 Data — (PDF) [file pmed.1001916.s001.pdf]

S1 Data for McHugh et al., "A Molecular Host Response Assay to Discriminate Between Sepsis and Infection-Negative Systemic Inflammation in Critically Ill Patients: Discovery and Validation in Independent Cohorts"

Translation Between Microarray and RT-qPCR Formats

1. Summary

Data for the present study were acquired on four different platforms:

Table 1: *Summary of Platforms Used for Data Collection*

Cohorts	Platform	Assigned Variable
Discovery Cohort	Affymetrix Microarray	w
Validation Cohort 1	RT-qPCR using TLDA cards on the Applied Biosystems 7900 platform	x
Validation Cohorts 2, 3, 5	RT-qPCR using TaqMan reagents on the Applied Biosystems 7500 FastDx platform	y
Validation Cohort 4	RT-qPCR using Asuragen reagents on the Applied Biosystems 7500 FastDx platform	z

The primers, probes, dyes and quenchers for the four singleplex qPCR reactions used in SeptiCyte Lab are given in the following table:

Table 2: *Primers, Probes, Dyes and Quenchers for the Singleplex qPCR Reactions Used in SeptiCyte Lab*

**Transcript**	**Reaction ID**	**Primers & Probes (5'-to-3')[1]**	**Dyes & Quenchers for TLDA Card Reactions[2,3]**	**Dyes & Quenchers for Single Tube TaqMan Reactions[4,5]**	**Dyes & Quenchers for Single Tube Asuragen Reactions[6]**
CEACAM4	IXP-045	FP: CCTGGTGTGTTTTCTGCTT<br>RP: TGAGGTCACGCTGGATG<br>Probe: CTCCAGGACTGGAAGGGCCA	Reporter: FAM<br>Quencher: NFQ	Reporter: JOE<br>Quencher: BHQ-1	Reporter: JOE<br>Quencher: proprietary
LAMP1	IXP-054	FP: CCACCGTCCTGCTCTTC<br>RP: CCTTGTAGGAAAAACCGGC<br>Probe: GTTCGGGATGAATGCAAGTTCTAGC	Reporter: FAM<br>Quencher: NFQ	Reporter: TAMRA<br>Quencher: BHQ-2	Reporter: FAM<br>Quencher: proprietary
PLA2G7	IXP-059<br>(Original reaction)[2]	FP: CCTCTGAGGCCTGGTGAAAA<br>RP: CCTGAATGCCCCAAGACCAT<br>Probe: TATCCACTTGTTGTTTTTTC	Reporter: FAM<br>Quencher: NFQ	Not used	Not used
	IXP-085<br>(Modified reaction)[4,6]	FP: TCAGACTCTTAGTGAAGATCAGAG<br>RP: CATCACCCAGTGGAAACATCC<br>Probe: CAGATGTGGTATTGCCCTGGATG	Not used	Reporter: FAM<br>Quencher: BHQ-1	Reporter: FAM<br>Quencher: proprietary
PLAC8	IXP-060	FP: TGGCAGACAGGCATGTG<br>RP: CAAAATGTGCCACAGAGACA<br>Probe: CTGTTTCAGCGACTGCGGAGT	Reporter: FAM<br>Quencher: NFQ	Reporter: Q670<br>Quencher: BHQ-3	Reporter: Q670<br>Quencher: proprietary

[1] FP = forward primer; RP = reverse primer
[2] used in TLDA card reactions (Validation Cohort 1)
[3] NFQ = Non-Fluorescent Quencher (Life Technologies, Inc.)
[4] used in single-tube TaqMan reactions (Validation Cohorts 2, 3, 5)
[5] BHQ = Black Hole Quencher (BioSearch Technologies, Inc.)
[6] used in single-tube Asuragen reactions (Validation Cohort 4)

The reaction conditions were as follows.

Reverse transcription, prior to qPCR with TLDA cards (Validation Cohort 1) or with TaqMan reagents (Validation Cohorts 2, 3, 5): Total RNA was reverse transcribed in a reaction volume of 20 uL using the TaqMan High Capacity cDNA Synthesis Kit (Applied Biosystems) according to the manufacturer's instructions. The thermal cycling program for reverse transcription was: 25°C for 10 min, then 37°C for 60 min, then 85°C for 5 min, then 4°C hold.

Reverse transcription, prior to qPCR with Asuragen reagents (Validation Cohort 4): Total RNA was reverse transcribed in a reaction volume of 15 uL using an Asuragen master mix specific for SeptiCyte Lab (proprietary formulation). The thermal cycling program for reverse transcription was: 42°C for 15 min, then 93°C for 2 min, then 25°C for 5 min, then 4°C hold.

qPCR with TLDA cards (Validation Cohort 1): TaqMan 2x Universal Master Mix (Applied Biosystems) was combined individually with cDNA samples at a 1:1 volume ratio, then loaded into TLDA cards and sealed according to the manufacturer's instructions. The thermal cycling program for qPCR was: 50°C for 2 min, then 94.5°C for 10 min, followed by 40 cycles of (97°C for 30 sec, then 59.7°C for 1 min).

qPCR with TaqMan reagents (Validation Cohorts 2, 3, 5): 15 uL reactions were conducted using TaqMan 2x Universal Master Mix (Applied Biosystems). The thermal

cycling program for qPCR was: 95°C for 10 min, followed by 40 cycles of (95°C for 15 sec, then 60°C for 1 min).

qPCR with Asuragen reagents (Validation Cohort 4): 15 uL reactions were conducted using Asuragen qPCR master mixes specific for SeptiCyte Lab (proprietary formulations). The thermal cycling program for qPCR was: 95°C for 10 min, followed by 40 cycles of (95°C for 15 sec, then 60°C for 1 min).

Here we show that one may compare or combine the raw data and calculated SeptiCyte Lab Scores obtained on the various platforms, by means of the linear transformations given in the following table:

Table 3: *Formulae for Linear Transformation of Score Values Between Platforms*

**Platform 1**	**Platform 2**	**Conversion Formula**
Affymetrix Microarray (w)	TLDA Card (x)	w = 0.758x -0.542
TLDA card (x)	TaqMan strip tube (y)	y = 0.9589x + 0.1573
TaqMan strip tube (y)	Asuragen strip tube (z)	z = 0.999y - 2.003

2. Translation Between Microarray and TLDA Card Formats

Samples from 25 cases (sepsis) vs. 37 controls (infection negative post-surgical patients with systemic inflammation) from the Discovery Cohort were tested using both Affymetrix microarrays and the Life Technology TaqMan Low Density Array (TLDA) cards. The correlation between microarray intensities and Ct values from TLDA cards for each of the four genes was established. After combining the individual microarray

intensity values or C_t values to produce the *SeptiCyte Lab* score, the values of this score were plotted for TLDA card data (X-axis) versus microarray data (w-axis) (**Figure 1**). As expected there was a highly linear relationship. The fitting parameters (w = mx + b) were: m = 0.758 ±0.036, b = -0.542 ±0.215, adjusted R^2 = 0.8806.

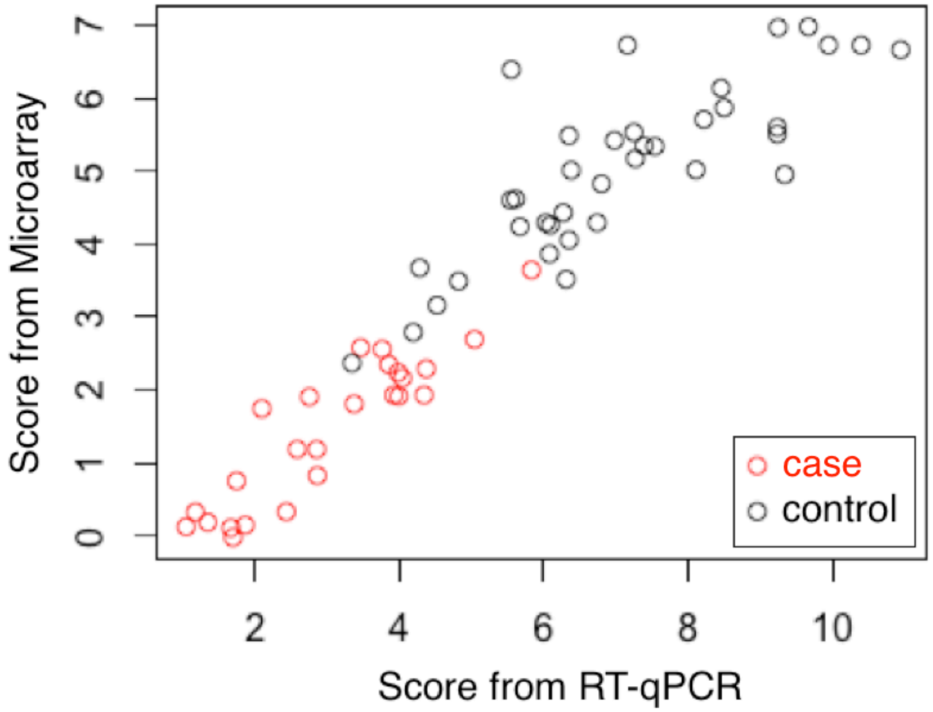

Figure 1: *Correlation of Microarray and TLDA Card Data.* A total of 62 RNA samples (25 cases, 37 controls) were examined on both Affymetrix microarrays and TLDA cards. *SeptiCyte Lab* scores were calculated and compared.

3. Translation Between TLDA Card and Single Tube TaqMan Formats

Forty test samples (purified RNAs) were chosen from Validation Cohort 1. These samples were assayed with *SeptiCyte Lab*, using TaqMan reagents on either the TLDA card / ABI 7900 platform, or the strip-tube / ABI 7500 FastDx platform.

Table 4 presents the raw data (Ct values) generated by the two methods. Note that, for the PLA2G7 assay, the sequences of the primer/probe set were changed in

migrating from the TLDA card (primer/probe set IXP-059) to the strip-tube format (primer/probe set IXP-085). For the other three RNA targets, the primer/probe sets were not changed between the two platforms.

Figures 2-5 present scatterplots of Ct values for PLA2G7, PLAC8, CEACAM4, LAMP1 respectively, in which x-axis = TLDA card data, and y-axis = strip-tube TaqMan data. **Figure 6** plots the correlation between *SeptiCyte Lab* Scores calculated from the TLDA card data and strip-tube TaqMan data.

Table 4*: Measured C_t Values Using TLDA Card vs. Strip-Tube TaqMan Format.* Samples (N=40) were from Validation Cohort 1.

				**TLDA Card (x)**				**Strip Tube TaqMan (y)**				
**Patient ID**	**ICU Admission ID**	**Sample Description**	**Asuragen RNA ID**	**CEACAM4 (IXP-045)**	**LAMP1 (IXP-054)**	**PLA2G7 (IXP-059)**	**PLAC8 (IXP-060)**	**IXP-045 (CEACAM4)**	**IXP-054 (LAMP1)**	**IXP-059 (PLA2G7)**	**IXP-085 (PLA2G7)**	**IXP-060 (PLAC8)**
12179	24669	3297869	S0085413	21.17	22.87	28.82	25.48	21.50	23.14	29.99	31.15	25.49
12188	24676	3302157	S0085425	24.2	23.34	28.75	24.22	24.57	23.72	28.83	29.85	24.45
12194	24680	3302412	S0085427	22.02	23.08	28.84	25.39	22.32	23.44	28.53	29.54	25.39
12211	24687	3303811	S0085433	23.48	23.34	29.19	25.07	23.81	23.65	29.42	30.34	25.35
12709	24992	3455833	S0085456	23.72	22.71	28.49	23.3	23.77	22.71	27.94	28.99	23.02
12720	25000	3458247	S0085461	21.94	23.01	27.79	24.78	22.17	23.22	27.67	28.70	24.70
12824	25066	3494913	S0085513	22.93	22.45	29.96	22.66	23.08	22.70	29.52	30.60	22.82
12868	25091	3509601	S0085537	24.09	23.73	31.05	23.01	24.18	23.64	30.26	31.59	22.74
12872	25094	3513752	S0085541	22.82	22.88	30.84	22.43	22.88	22.99	29.84	32.06	22.05
12885	25098	3518677	S0085548	24.19	23.57	26.2	24.34	24.51	23.82	26.51	27.50	24.40
12905	25110	3523664	S0085558	24.19	23.97	27.2	24.16	24.46	24.25	27.19	28.38	24.38
12916	25119	3528292	S0085566	21.71	23.26	27.64	25.54	21.72	22.89	27.60	28.42	25.69
12926	25123	3530376	S0085572	22.95	22.94	32.56	21.93	22.91	23.08	31.94	33.60	21.84
12260	24709	3316840	S0085815	22.88	25.48	30	26.95	22.18	24.27	29.73	31.02	26.74
12291	24721	3322856	S0085826	21.98	24.25	30.63	26.96	22.24	24.39	30.45	31.67	27.11
12314	24740	3332030	S0085834	23.72	25.14	30.97	26.9	23.81	25.08	30.87	33.02	26.92
12352	24769	3344784	S0085844	24.29	23.33	30.51	22.91	24.37	23.22	30.61	32.38	22.75
12354	24770	3345372	S0085845	23.88	24.53	28.18	25.45	23.59	23.69	28.25	29.18	25.29
12376	24786	3351972	S0085852	24.08	24.38	31.41	21.81	23.82	24.00	32.28	34.00	21.77
12384	24791	3354263	S0085857	23.88	24.24	31.86	21.82	23.78	24.17	31.90	33.27	21.92
12404	24804	3360593	S0085867	26.65	25.07	33.99	22.94	26.10	24.67	33.09	35.44	22.75
12399	24801	3360647	S0085869	24.05	24.97	27.22	24.57	23.80	24.25	26.97	27.79	24.18
12415	24811	3364135	S0085877	24.06	23.88	28	24.89	23.99	23.60	27.78	28.86	24.94
12604	24927	3421854	S0085896	25.86	25.6	27.4	25.07	25.46	24.67	27.31	28.03	24.98
12637	24950	3432950	S0085917	24.84	24.9	30.37	25.43	24.80	24.68	30.22	31.35	25.42
12679	24972	3444591	S0085940	24.38	25.33	29.99	24.57	24.56	25.17	29.72	31.76	24.67
12682	24974	3445120	S0085942	25.03	25.25	30.49	25.59	25.36	25.53	30.65	31.97	25.88

				TLDA Card (x)				Strip Tube TaqMan (y)				
**Patient ID**	**ICU Admission ID**	**Sample Description**	**Asuragen RNA ID**	**CEACAM4 (IXP-045)**	**LAMP1 (IXP-054)**	**PLA2G7 (IXP-059)**	**PLAC8 (IXP-060)**	**IXP-045 (CEACAM4)**	**IXP-054 (LAMP1)**	**IXP-059 (PLA2G7)**	**IXP-085 (PLA2G7)**	**IXP-060 (PLAC8)**
12684	24976	3446582	S0085943	26.1	26.11	33.02	22.21	26.04	25.79	32.35	35.91	22.33
12757	25027	3471688	S0085971	24.49	24.53	28.41	25.43	24.58	24.70	28.27	29.33	25.56
12759	25028	3471799	S0085972	24.85	24.1	27.53	24.57	25.01	24.27	27.48	28.62	24.62
12763	25030	3471927	S0085973	24.58	23.82	28.13	24.75	24.58	23.96	28.04	29.17	24.87
12778	25040	3478934	S0085984	22.18	23.86	32.68	25.83	22.31	23.98	32.06	33.90	25.97
12795	25048	3483639	S0085993	22.78	22.85	26.74	25.06	22.57	22.87	26.42	27.46	24.99
12504	24861	3393976	S0086123	25.41	25.14	31.07	23.3	25.60	25.31	31.07	32.31	23.43
12536	24869	3399431	S0086133	23.73	23.99	29.92	24.82	23.94	24.27	29.87	31.26	25.15
12553	24906	3404773	S0086144	23.79	24.14	31.23	21.9	24.10	24.49	31.14	32.39	22.01
12566	24912	3408224	S0086151	23.18	23.6	30.89	25.42	23.45	23.72	30.61	32.50	25.30
12693	24981	3450704	S0086174	23.4	22.91	31.94	21.1	23.66	23.10	31.61	33.27	21.14
12319	24745	3333502	S0086300	23.81	23.1	30.12	23.28	23.82	23.14	29.62	31.03	23.23
12337	24759	3339033	S0086306	25.37	25.79	30.78	25.89	25.53	25.61	30.66	31.79	25.63

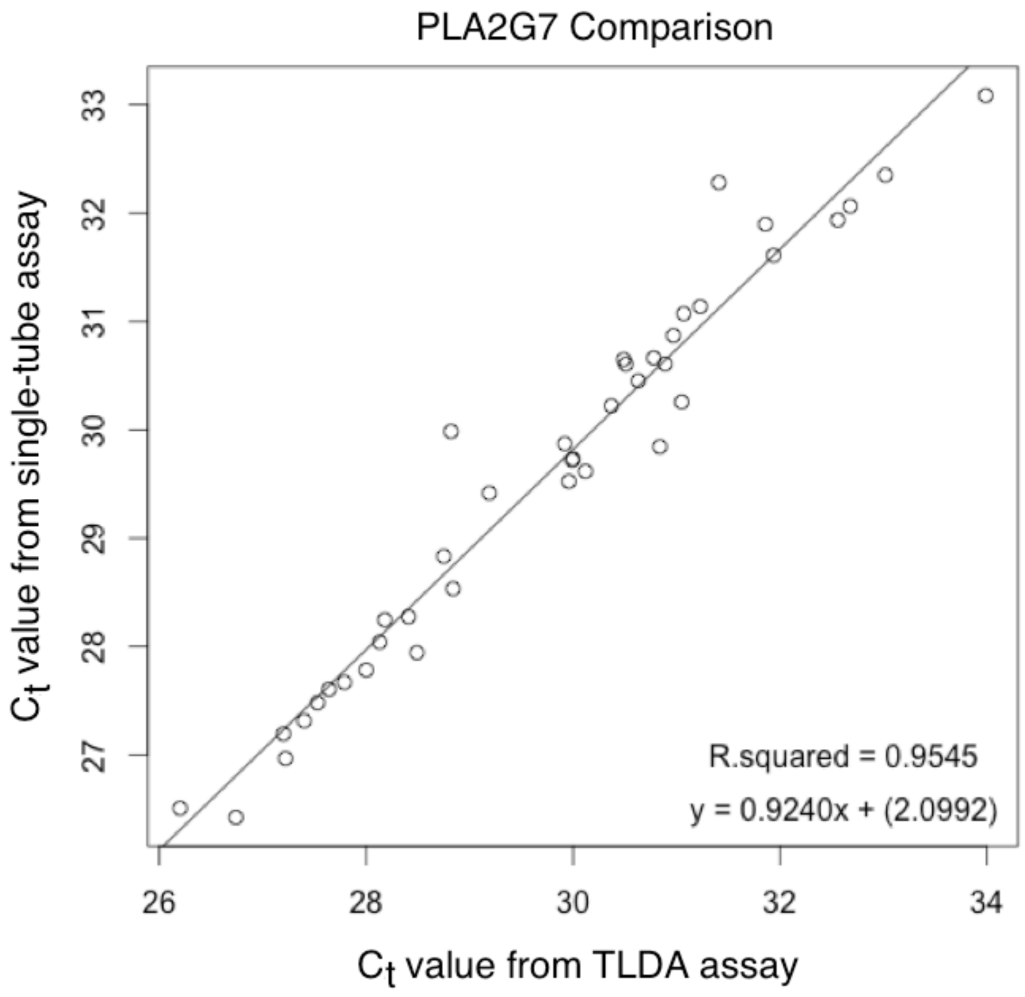

Figure 2: *Scatterplot of C_t Values for TLDA Card vs. Strip-Tube TaqMan format, for PLA2G7 RNA Transcript.* TaqMan reagents were used for both assays. Samples (n=40) were from Validation Cohort 1. x-axis = TLDA card assay, y-axis = strip-tube TaqMan assay.

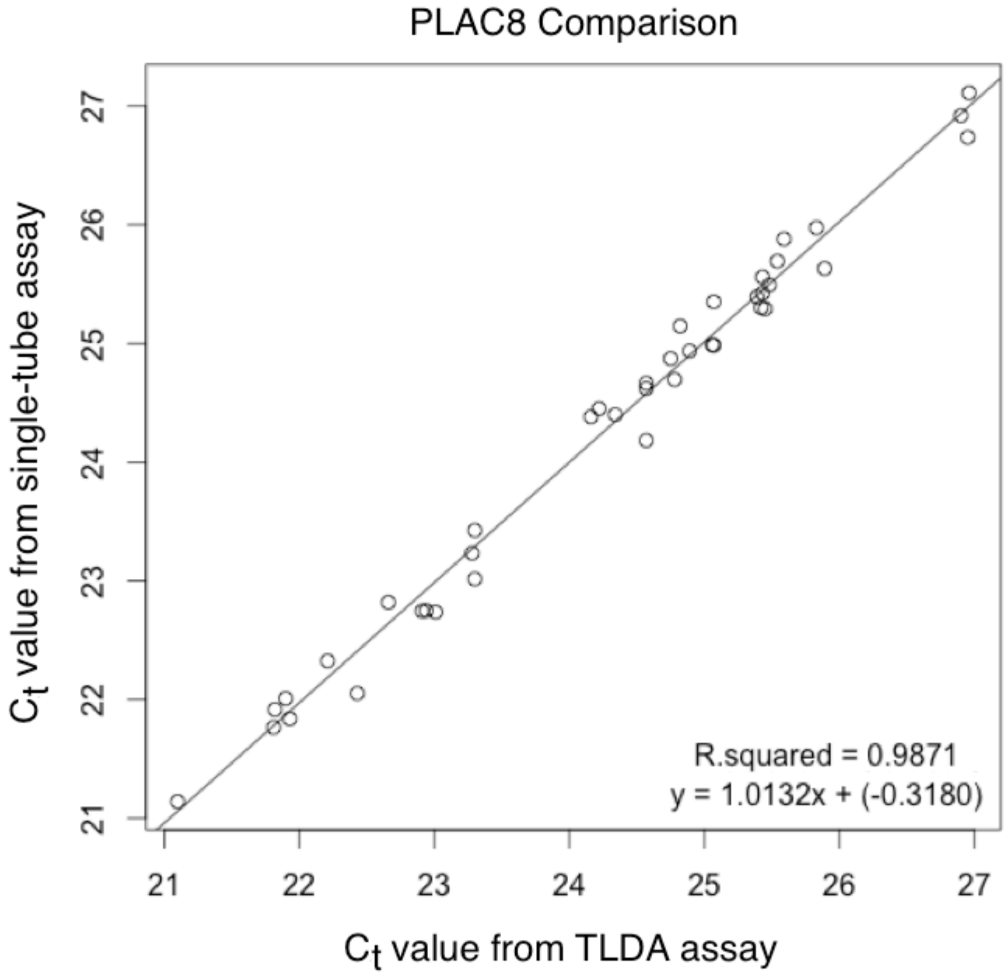

Figure 3: *Scatterplot of C_t Values for TLDA Card vs. Strip-Tube TaqMan Format, for PLAC8 RNA Transcript.* TaqMan reagents were used for both assays. Samples (n=40) were from Validation Cohort 1. x-axis = TLDA card assay, y-axis = strip-tube TaqMan assay.

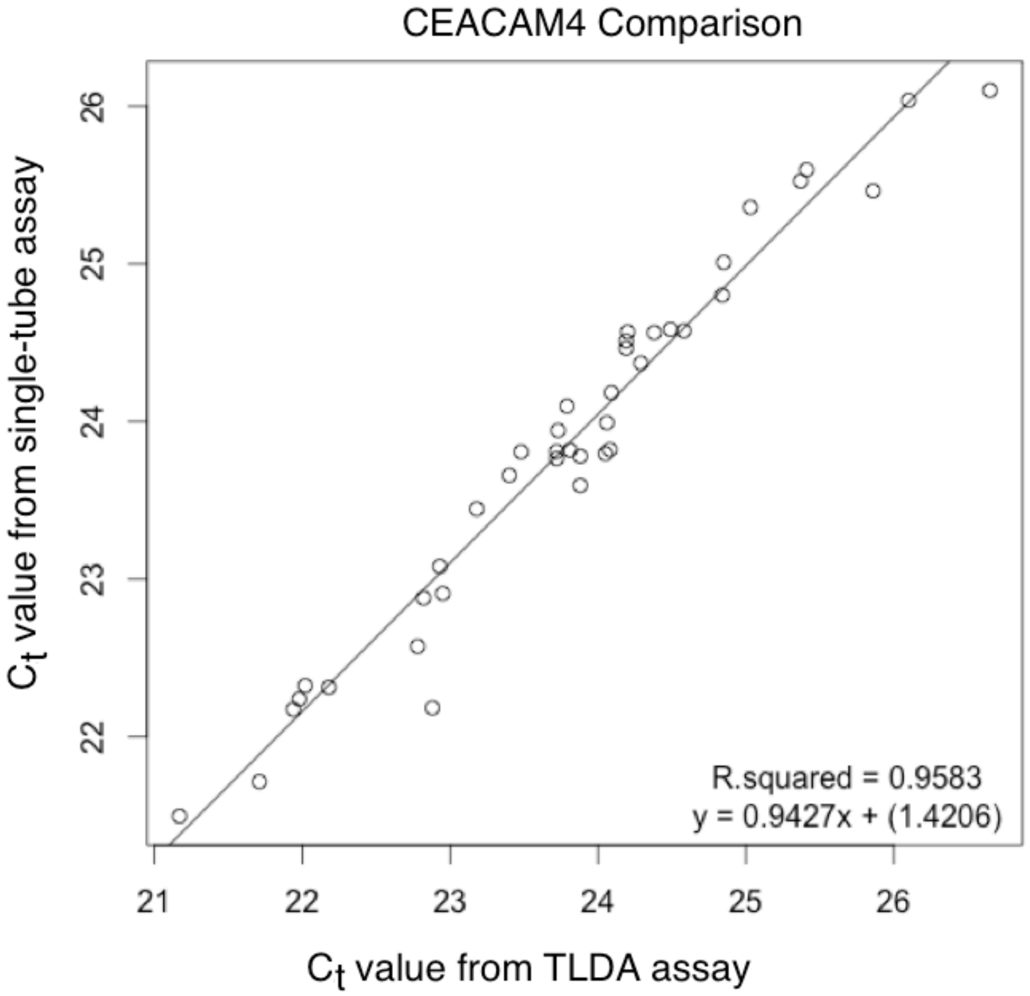

Figure 4: *Scatterplot of C_t Values for TLDA Card vs. Strip-Tube TaqMan Format, for CEACAM4 RNA Transcript.* TaqMan reagents were used for both assays. Samples (n=40) were from Validation Cohort 1. x-axis = TLDA card assay, y-axis = strip-tube assay.

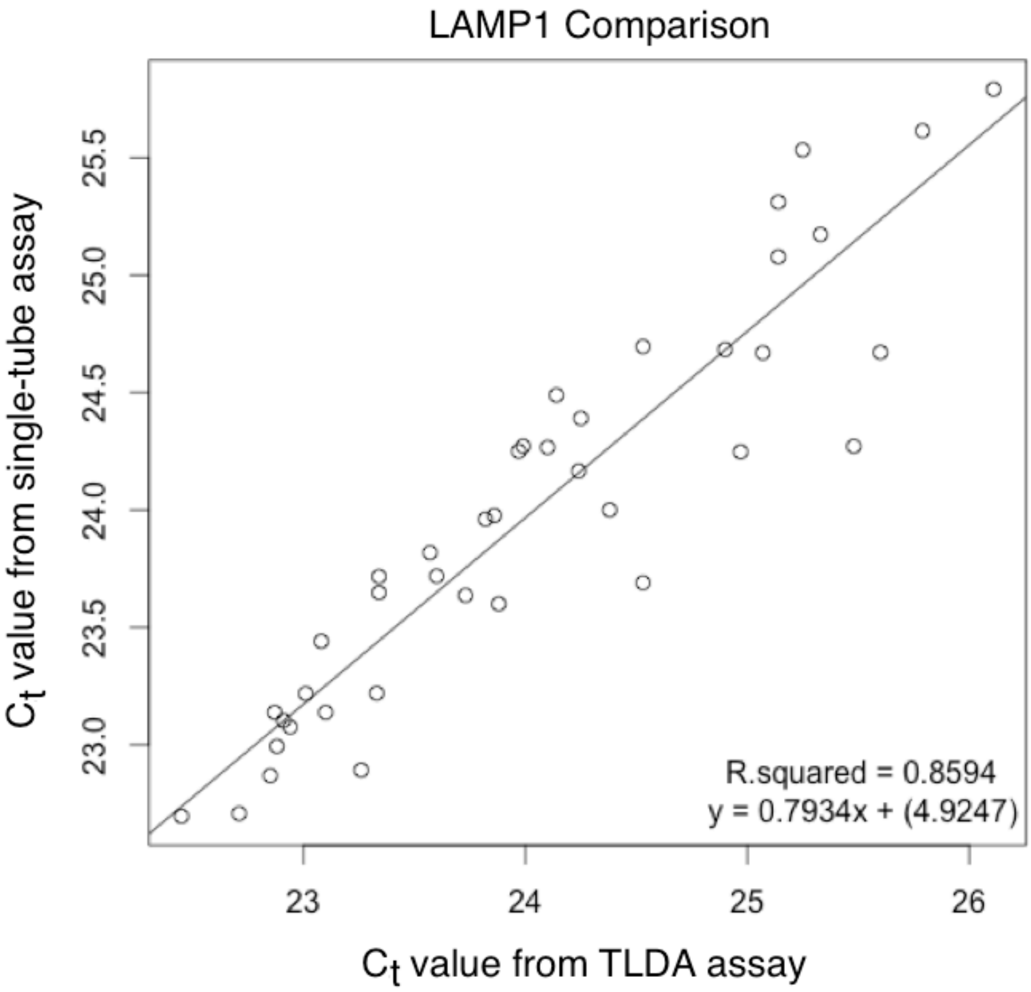

Figure 5: *Scatterplot of C_t Values for TLDA Card vs. Strip-Tube TaqMan Format, for LAMP1 RNA Transcript.* TaqMan reagents were used for both assays. Samples (n=40) were from Validation Cohort 1. x-axis = TLDA card assay, y-axis = strip-tube TaqMan assay.

The *SeptiCyte Lab* Score is calculated from the individual C_t values, as follows:

Score = $(C_{t,1}-C_{t,2}) + (C_{t,3}-C_{t,4})$, where

$C_{t,1}$ = threshold C_t value for PLA2G7

$C_{t,2}$ = threshold C_t value for PLAC8

$C_{t,3}$ = threshold C_t value for CEACAM4

$\mathbf{C_{t,4}}$ = threshold C_t value for LAMP1 (Eq. 1)

In **Figure 6**, the *SeptiCyte Lab* Score is plotted for TLDA card assay data (x-axis) vs. strip tube assay data (y-axis).

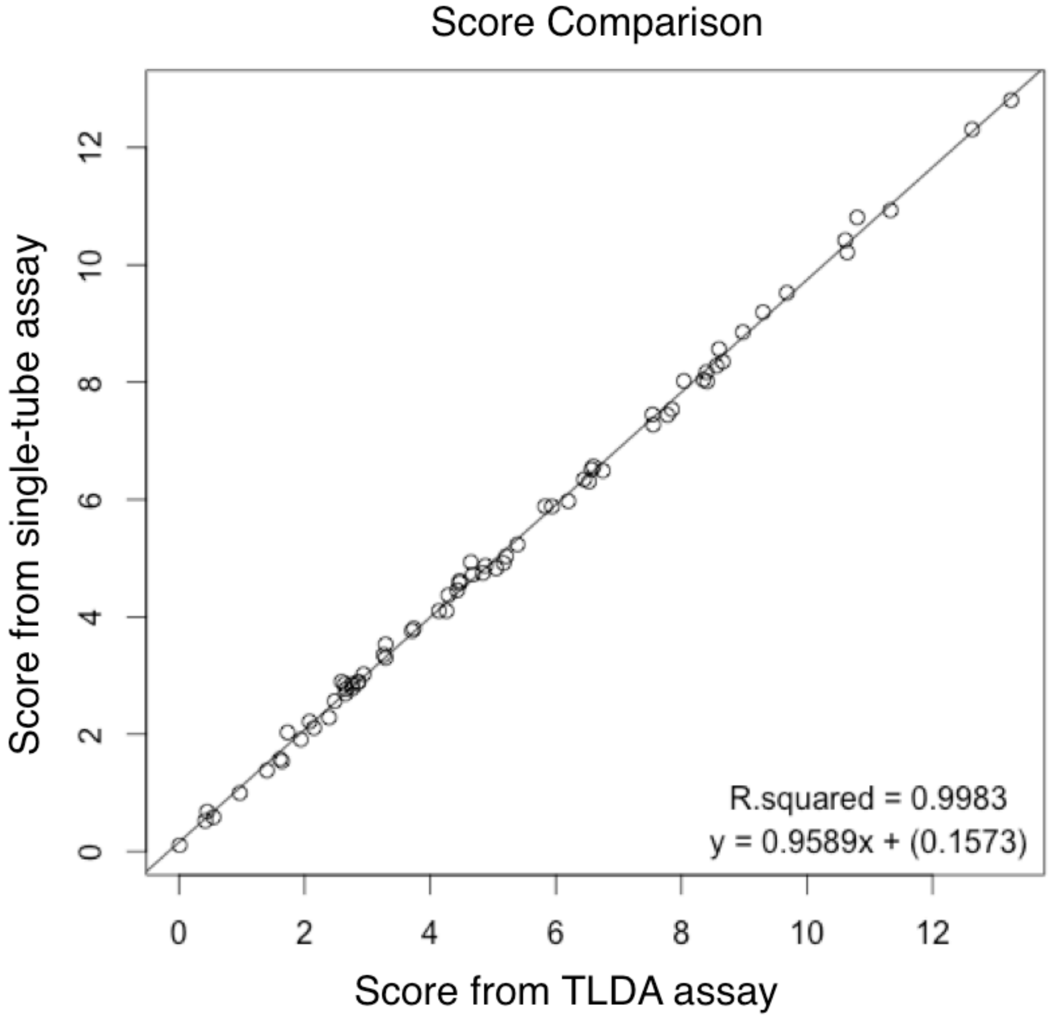

Figure 6: *Plot of SeptiCyte Lab Score for TLDA card vs. Strip Tube TaqMan Format.* TaqMan reagents used for both assays. Samples (n=40) were from Validation Cohort 1. x-axis = TLDA card assay, y-axis = strip-tube TaqMan assay.

Table 5 summarizes the linear fitting parameters for the graph of TLDA card assay data vs. strip-tube assay data, from Figures 2-6. Although the LAMP1 fit is weakest, when all the C_t values are combined the resultant *SeptiCyte Lab* score has a very

strong and nearly unbiased linear fit.

Table 5: *Linear Fitting Parameters*[1]

	y = mx + b		
**Gene or Score**	**m**	**b**	R^2
PLA2G7	0.9240	2.0992	0.9545
PLAC8	1.0132	-0.3180	0.9871
CEACAM4	0.9427	1.4206	0.9583
LAMP1	0.7934	4.9247	0.8594
SeptiCyte® *Lab* Score	0.9589	0.1573	0.9983

[1]x = TLDA card data, and y = strip-tube TaqMan data.

Bias is defined as the difference in results between the strip-tube assay (new method) and the TLDA card assay (old method). Bias values are stated explicitly in **Table 6** (C_t values) and **Table 7** (Score). Over the usable C_t range of the assay (22 cycles to 36 cycles, for each RNA transcript) the absolute value of the C_t bias is no larger than 0.64 cycles per transcript, except for LAMP1 which has a bias that increases to > 2.5 cycles at high cycle numbers.

Because of how the *SeptiCyte Lab* score is defined, the bias in LAMP1 is offset by the bias in the other three RNA transcripts. The end result is that the absolute value of the bias in *SeptiCyte Lab* score is less than 0.42 cycles, over the entire reportable score range (0-14 units). Given that the estimated standard deviation of the *SeptiCyte Lab* score (for replicate determinations) is about ± 0.3 cycles, the score bias appears tolerably small.

Table 6: *Bias Table for Individual C_t Values.* TaqMan reagents were used in both cases.

TLDA card C_t value	Strip-tube C_t value							
	PLA2G7		PLAC8		CEACAM4		LAMP1	
	Value	Bias[1]	Value	Bias[1]	Value	Bias[1]	Value	Bias[1]
22	22.4272	0.4272	21.9724	-0.0276	22.16	0.16	22.3795	0.3795
24	24.2752	0.2752	23.9988	-0.0012	24.0454	0.0454	23.9663	-0.0337
26	26.1232	0.1232	26.0252	0.0252	25.9308	-0.0692	25.5531	-0.4469
28	27.9712	-0.0288	28.0516	0.0516	27.8162	-0.1838	27.1399	-0.8601
30	29.8192	-0.1808	30.078	0.078	29.7016	-0.2984	28.7267	-1.2733
32	31.6672	-0.3328	32.1044	0.1044	31.587	-0.413	30.3135	-1.6865
34	33.5152	-0.4848	34.1308	0.1308	33.4724	-0.5276	31.9003	-2.0997
36	35.3632	-0.6368	36.1572	0.1572	35.3578	-0.6422	33.4871	-2.5129

[1]Bias = C_t (strip-tube TaqMan assay - C_t (TLDA card assay).

Table 7: *Bias Table for SeptiCyte Lab Score.* TaqMan reagents were used in both cases.

TLDA Card Score	Strip Tube Score	Bias
0	0.1573	0.1573
2	2.0751	0.0751
4	3.9929	-0.0071
6	5.9107	-0.0893
8	7.8285	-0.1715
10	9.7463	-0.2537
12	11.6641	-0.3359
14	13.5819	-0.4181

[1]Bias = Score (strip-tube TaqMan assay) - Score (TLDA card assay).

4. Translation Between Strip-Tube TaqMan and Strip-Tube Asuragen Data Formats

Sixty test samples (purified RNAs), described in **Table 8** below, were assayed with *SeptiCyte Lab* using either TaqMan or Asuragen reagents in a strip-tube format. All assays were run on the ABI 7500 FastDx platform. **Table 8** presents the C_t values generated by the two methods.

Table 8: *C_t values and SeptiCyte Lab Scores Generated with TaqMan vs. Asuragen Reagents, in Strip-Tube Format*

Sample	RNA Input (ng)	MARS ICU.ID	Cohort	Asuragen Reagents (z)					TaqMan Reagents (y)				
				Ct values				Score	Ct values				Score
				CEACAM4	LAMP1	PLA2G7	PLAC8		CEACAM4	LAMP1	PLAC8	PLA2G7	
S0091749	35.7	13342	3	24.6	27.1	30.4	25.7	2.23	23.0	23.9	26.0	30.6	3.70
S0091599	55.2	13083	3	27.1	26.0	29.0	23.1	6.99	27.2	24.4	24.1	30.3	8.98
S0085861	69.1	12360	Not from study	23.2	25.6	33.6	23.7	7.48	23.4	24.3	25.4	33.5	7.22
S0085817	81.4	12276	3	22.1	24.6	26.5	21.1	2.97	23.0	23.8	22.9	29.2	5.50
S0085668	90.2	4706	Not from study	23.3	24.7	30.3	21.1	7.81	24.8	24.3	23.2	32.7	10.07
S0086223	118.4	5043	Not from study	22.3	24.4	27.7	22.2	3.44	23.5	24.0	24.6	30.6	5.41
S0091757	137.9	13394	3	22.6	24.2	27.1	22.2	3.31	23.4	23.5	24.4	29.6	5.07
S0085651	184.7	4599	3	23.4	25.0	25.7	22.1	2.01	25.1	25.0	24.9	29.4	4.59
S0091844	185.7	13536	3	20.6	25.1	29.6	24.0	1.11	22.3	24.9	27.5	33.0	2.88
S0085806	258.8	12254	Not from study	21.2	23.7	28.8	18.4	7.84	23.8	24.3	22.0	32.4	9.86
S0086248	270.1	5111	Not from study	21.2	22.5	24.1	19.5	3.28	23.3	23.1	23.0	28.0	5.24
S0085980	276	12773	3	22.4	23.4	25.5	21.1	3.34	25.1	24.3	25.0	29.9	5.66
S0085700	283.1	4625	3	21.9	24.1	26.5	20.7	3.62	25.0	25.2	24.8	31.3	6.32
S0092282	300	13150	3	21.1	24.1	24.0	21.7	-0.73	22.8	24.4	25.5	28.3	1.18
S0085505	300	12812	Not from study	20.0	22.5	23.8	21.3	0.06	22.5	23.7	25.5	28.5	1.78
S0085526	300	12848	3	20.4	22.2	24.0	21.6	0.53	23.0	23.4	26.0	28.8	2.31
S0091925	300	13318	3	21.0	22.2	23.1	21.3	0.66	23.1	22.9	25.1	27.3	2.42
S0085573	300	12927	3	21.6	23.1	24.4	21.7	1.20	24.6	24.6	26.2	29.3	3.17
S0085517	300	12834	3	20.2	22.7	24.8	21.0	1.25	22.8	24.0	25.2	29.7	3.30
S0085901	300	12613	3	22.1	23.1	23.8	21.4	1.36	24.5	24.0	25.3	28.4	3.61

Sample	RNA Input (ng)	MARS ICU.ID	Cohort	Asuragen Reagents (z)					TaqMan Reagents (y)				
				Ct values				Score	Ct values				Score
				CEACAM4	LAMP1	PLA2G7	PLAC8		CEACAM4	LAMP1	PLAC8	PLA2G7	
S0085406	300	12150	3	22.0	23.4	23.1	20.2	1.52	24.8	24.8	24.5	28.2	3.76
S0086259	300	5168	3	21.4	24.3	26.8	22.0	1.95	23.2	24.7	25.6	30.9	3.80
S0085889	300	12444	Not from study	23.7	24.5	25.1	22.2	2.21	26.1	25.3	25.8	29.9	4.83
S0091732	300	13469	3	22.0	23.8	26.2	22.1	2.38	24.3	24.8	25.8	30.4	4.07
S0086137	300	12540	3	21.1	23.1	26.0	21.5	2.57	23.8	24.2	25.7	30.3	4.21
S0085747	300	4770	3	23.0	23.9	25.7	22.0	2.80	25.5	24.9	25.9	30.6	5.30
S0091538	300	13030	Not from study	22.0	24.0	26.1	21.2	2.88	24.2	24.6	24.9	30.2	4.78
S0085743	300	4738	3	19.1	22.7	28.4	21.7	2.96	21.1	23.4	25.9	32.5	4.28
S0085717	300	4668	3	24.1	24.5	25.7	22.1	3.13	26.4	25.4	25.7	30.2	5.54
S0085643	300	4589	Not from study	21.6	23.0	24.0	19.4	3.18	24.9	24.6	23.7	29.1	5.76
S0091842	300	13540	3	20.3	22.2	26.2	21.0	3.25	22.4	22.7	24.9	30.7	5.50
S0085629	300	4506	3	22.0	22.8	24.4	20.2	3.33	24.8	24.0	24.2	29.2	5.83
S0085996	300	4324	Not from study	20.8	23.3	27.3	21.3	3.52	23.3	24.7	25.7	32.1	5.01
S0091919	300	13191	3	22.3	23.4	26.0	21.2	3.70	24.3	24.1	24.5	29.9	5.58
S0085714	300	4661	3	22.5	24.3	28.6	23.0	3.86	25.0	25.4	27.0	32.8	5.44
S0086053	300	4490	3	21.2	22.3	26.1	20.8	4.29	24.0	23.8	24.9	30.7	5.94
S0086220	300	5039	Not from study	21.4	22.5	28.0	22.4	4.53	23.5	23.4	26.5	32.2	5.88
S0085590	300	12956	3	22.2	22.1	24.0	19.4	4.64	25.0	23.4	23.4	28.8	6.99
S0085686	300	4822	3	21.7	23.3	28.3	21.9	4.93	24.1	23.9	25.9	32.8	7.14
S0085680	300	4810	3	22.5	25.3	30.0	22.3	5.05	24.8	26.0	26.2	34.3	7.00
S0091908	300	13316	Not from study	22.6	23.6	27.6	21.3	5.32	24.6	24.1	24.6	31.6	7.51
S0085987	300	12788	3	22.2	22.9	26.1	20.1	5.34	25.0	24.3	24.1	30.8	7.37
S0085701	300	4626	Not from study	22.1	22.4	25.1	19.3	5.40	24.6	23.3	23.1	29.8	7.95

Sample	RNA Input (ng)	MARS ICU.ID	Cohort	Asuragen Reagents (z)					TaqMan Reagents (y)				
				Ct values				Score	Ct values				Score
				CEACAM4	LAMP1	PLA2G7	PLAC8		CEACAM4	LAMP1	PLAC8	PLA2G7	
S0085646	300	4593	Not from study	23.4	23.8	27.4	21.5	5.47	26.1	25.0	25.5	31.9	7.55
S0085836	300	12328	Not from study	23.2	23.3	27.6	21.2	6.28	25.4	24.2	24.8	32.0	8.43
S0085684	300	4818	3	20.9	21.8	26.3	19.1	6.32	23.3	22.8	22.7	31.0	8.69
S0086230	300	5055	3	23.1	23.5	29.0	22.0	6.61	26.1	24.9	26.1	33.0	8.12
S0085588	300	12953	Not from study	23.2	23.0	28.0	21.5	6.78	26.0	24.6	25.7	32.5	8.21
S0091589	300	13065	3	22.3	24.1	28.3	19.4	7.10	24.9	24.7	23.0	32.6	9.80
S0091819	300	13581	3	22.4	24.0	28.4	19.6	7.11	25.0	24.6	23.7	32.9	9.64
S0085794	300	4917	3	22.9	23.3	26.9	18.8	7.68	26.1	25.1	23.1	31.9	9.83
S0086224	300	5044	3	20.1	22.1	29.3	19.6	7.75	22.6	23.2	23.7	34.2	9.92
S0086206	300	4998	3	20.5	22.3	28.0	18.2	7.95	23.2	23.4	22.1	32.6	10.40
S0086188	300	4931	3	22.4	23.0	28.2	19.1	8.50	25.2	24.1	23.0	32.9	11.01
S0086168	300	12593	Not from study	21.4	23.4	29.3	18.1	9.14	24.6	24.6	22.2	34.3	12.21
S0086197	300	4946	3	22.5	23.2	28.4	18.5	9.30	25.0	23.9	22.2	33.0	11.89
S0085855	308.3	12381	3	21.2	23.4	26.3	22.1	2.05	23.5	24.2	25.9	30.7	4.08
S0091930	310.1	13235	3	22.3	23.2	25.8	21.9	3.02	24.4	23.7	25.2	29.6	5.15
S0085638	323.7	4554	Not from study	22.2	23.5	32.0	22.1	8.64	25.3	24.9	26.2	34.3	8.52
S0086195	330.9	4941	Not from study	21.8	23.2	28.4	21.3	5.75	24.2	24.2	25.2	32.6	7.46

Figure 7 presents C_t correlation plots for PLA2G7, PLAC8, CEACAM4 and LAMP1, in which values from Asuragen reagents (z-axis, horizontal) are plotted against values from TaqMan reagents (y-axis, vertical). All reactions were run in strip-tube format. **Figures 8, 9** present two alternate representations of the correlation between *SeptiCyte Lab* scores calculated from the TaqMan C_t data vs. the Asuragen C_t data.

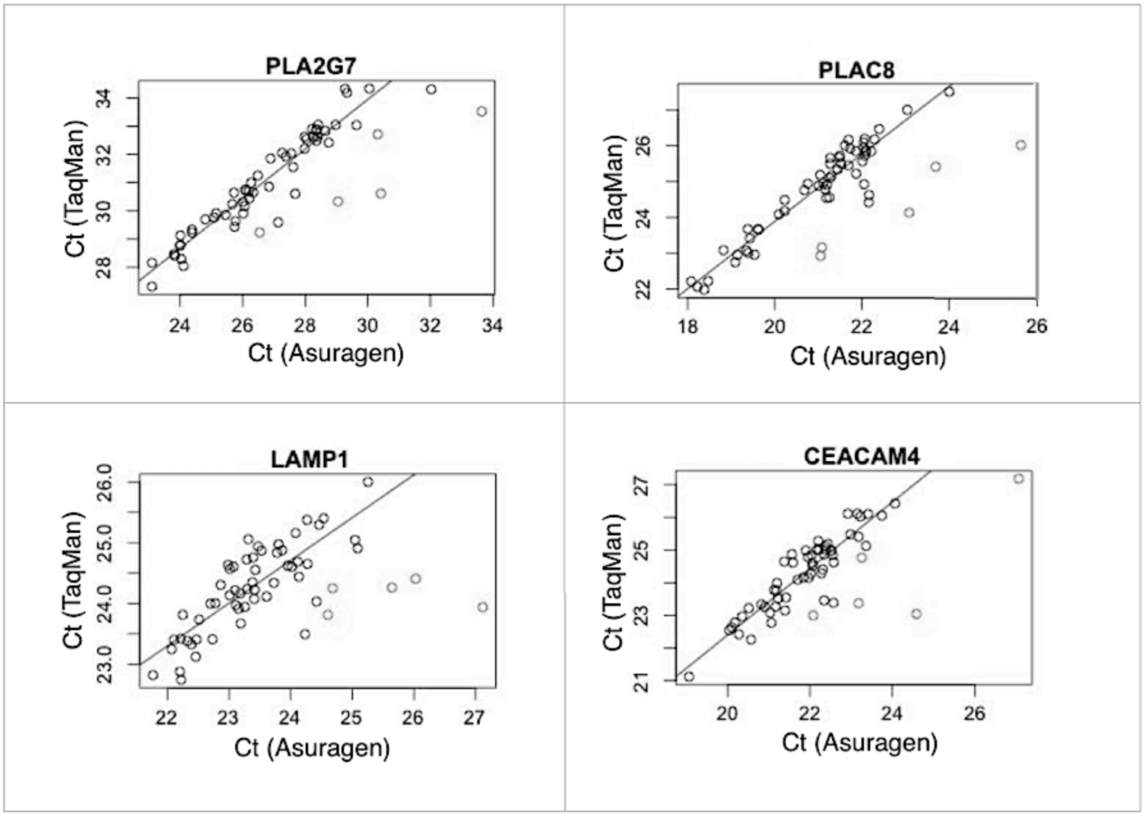

***Figure** 7: Correlation Plots of C_t Values, for Asuragen Chemistry vs. TaqMan Chemistry in strip-tube format.* The samples listed in Table 8 (n=60) were used to generate this plot. z-axis (horizontal) = strip-tube assay with Asuragen reagents, y-axis (vertical) = strip-tube assay with TaqMan reagents.

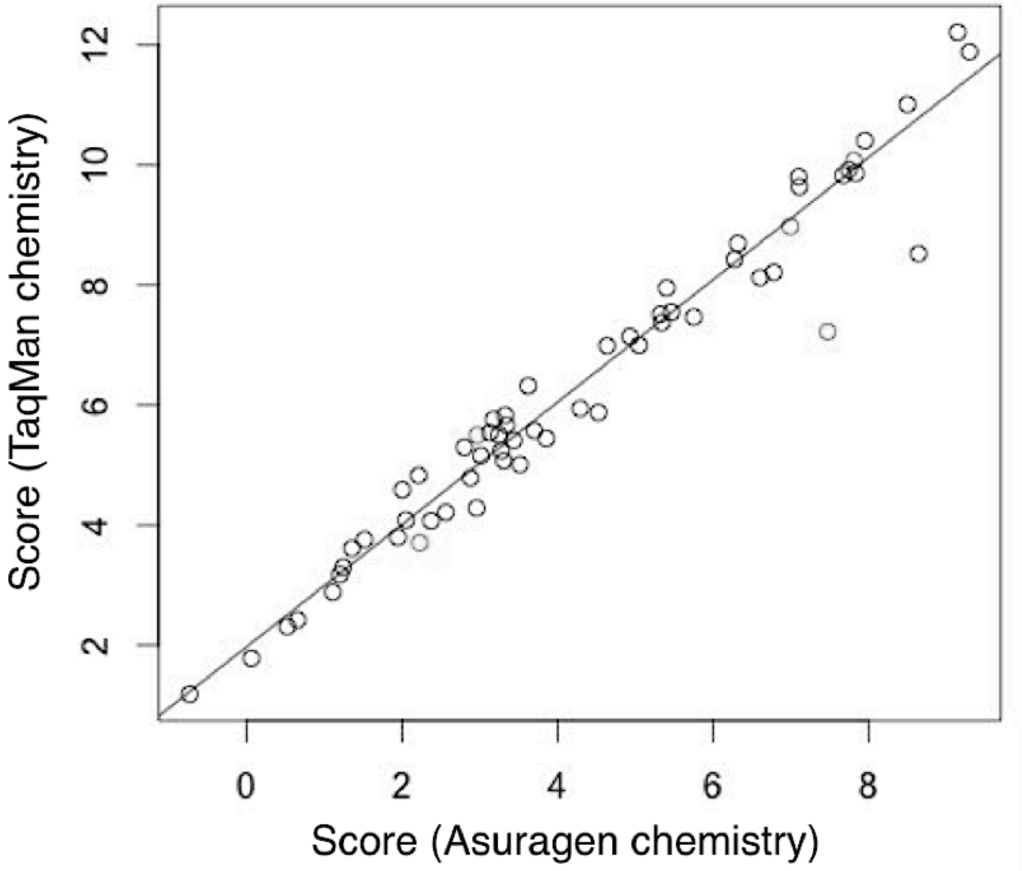

Figure 8: *Correlation Between SeptiCyte Lab Scores, for Asuragen Chemistry (z-axis, horizontal) vs. TaqMan Chemistry (y-axis, vertical).* Runs were in single-tube format, and were selected to span the entire range of *SeptiCyte Lab* scores. The samples listed in Table 8 (n=60) were used to generate this plot. The plot obeys the linear equation y = 1.0013 z + 2.006, with correlation coefficient R^2 = 0.9522.

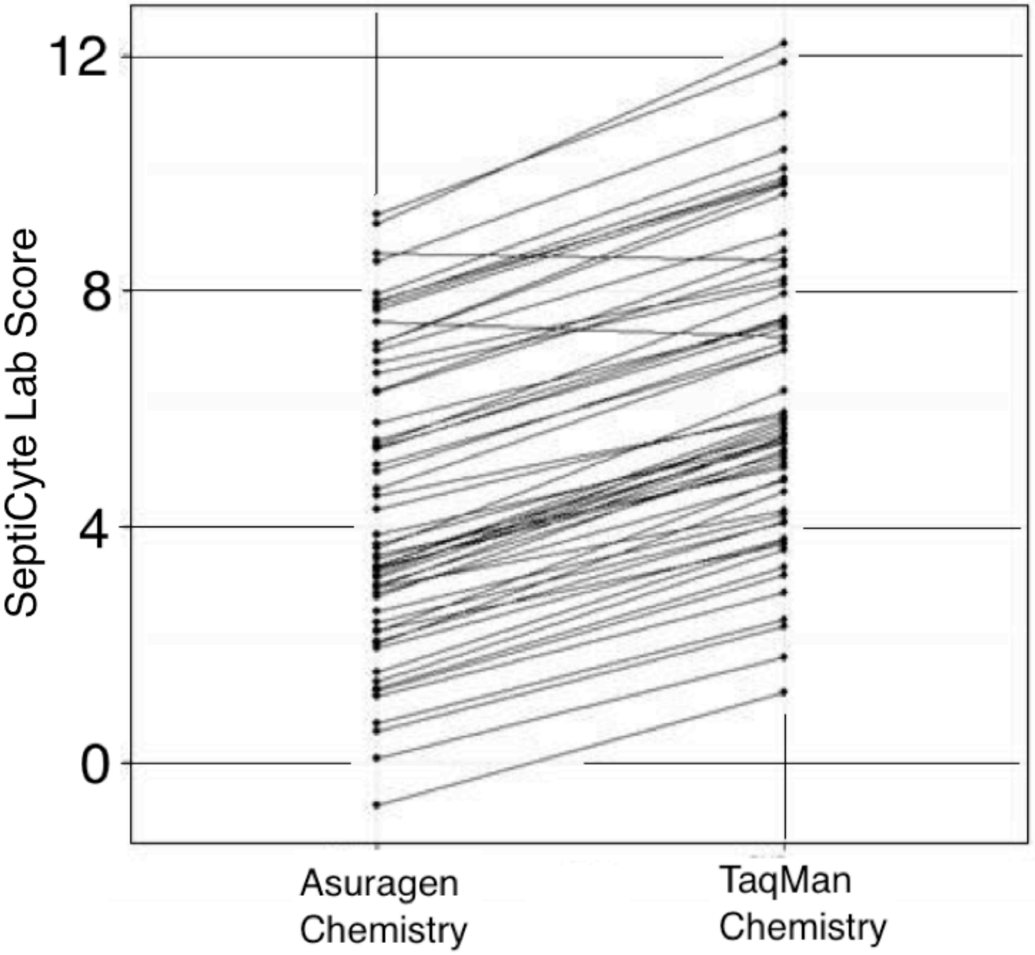

Figure 9: *Rank Order Plot for Asuragen Chemistry vs. TaqMan Chemistry.* Each line in this plot represents the shift in *SeptiCyte*® *Lab* score that occurs upon migrating from the TaqMan chemistry (right-hand points in plot) to the Asuragen chemistry (left-hand points in plot). With few exceptions, the rank order of *SeptiCyte Lab* scores for the tested samples is preserved.
