# Supplementary material for: A Molecular Host Response Assay to Discriminate Between Sepsis and Infection-Negative Systemic Inflammation in Critically Ill Patients: Discovery and Validation in Independent Cohorts
Source: PLoS Med. 2015 Dec 8;12(12):e1001916. doi: 10.1371/journal.pmed.1001916 (PMC4672921; doi:10.1371/journal.pmed.1001916)
Supplement: S3 Data — (PDF) [file pmed.1001916.s003.pdf]

S3 Data for McHugh et al., "A Molecular Host Response Assay to Discriminate Between Sepsis and Infection-Negative Systemic Inflammation in Critically Ill Patients: Discovery and Validation in Independent Cohorts"

Test of the *SeptiCyte Lab* Classifier Against an Independent Microarray Dataset

The *SeptiCyte Lab* classifier received a preliminary validation by ROC curve analysis of an independent, publicly available dataset (#E-MTAB-1548 from the EMBL-EBI ArrayExpress database). This external dataset presents mRNA profiles from PAXgene blood RNA samples collected in Spain from 39 cases (post-surgical patients with septic shock) vs. 34 controls (non-sepsis post-surgical patients). Some characteristics of the E-MTAB-1548 dataset are presented in **Table 1**. A ROC curve for the *SeptiCyte Lab* classifier is given in **Figure 1**.

Table 1: *Description of the E-MTAB-1548 Dataset from EMBL-EBI*[1]

**Sample Type**	**Microarray Chip**	**Comparison Groups**	**AUC (95% CI)**	**Comments**
PAXgene Blood RNA	Agilent A-MEXP-2183 (4x44k v2)	Cases: Septic shock, post-surgical (N= 39)<br><br>Controls: Non-sepsis, post-surgical (N = 34)	0.89 (0.81-0.98)	Clinical information limited. Septic shock is an extreme state of sepsis. Different microarray platform from that used in the present study. Geographic origin of cohort: Spain.

[1]Dataset obtained from www.ebi.ac.uk/arrayexpress/

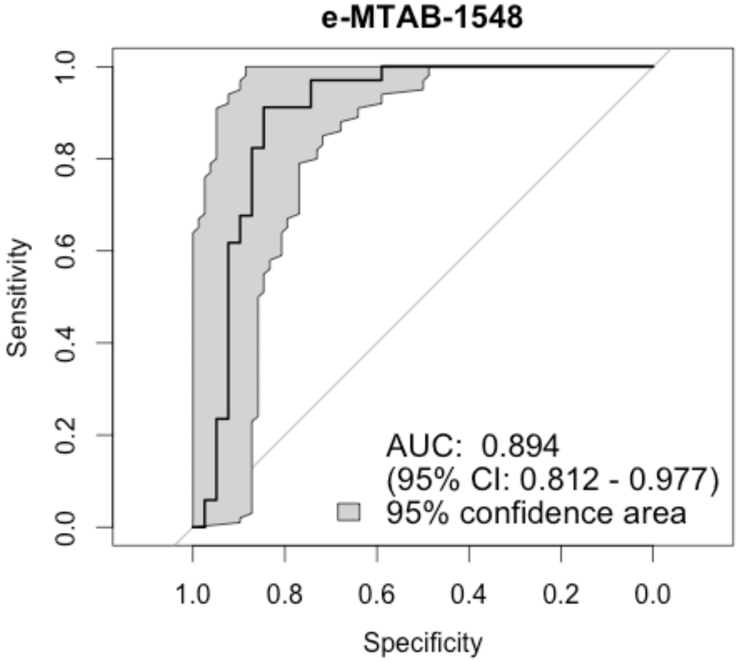

Figure 1: *ROC curve for analysis of independent microarray dataset # E-MTAB-1548, using the SeptiCyte Lab classifier.* This dataset was obtained from the EMBL-EBI ArrayExpress database. The ROC analysis compares samples from 39 cases (post-surgical patients with septic shock) vs. 34 controls (non-septic post-surgical patients). AUC = 0.89 (95% CI: 0.81-0.98).
