# Supplementary material for: A Molecular Host Response Assay to Discriminate Between Sepsis and Infection-Negative Systemic Inflammation in Critically Ill Patients: Discovery and Validation in Independent Cohorts
Source: PLoS Med. 2015 Dec 8;12(12):e1001916. doi: 10.1371/journal.pmed.1001916 (PMC4672921; doi:10.1371/journal.pmed.1001916)
Supplement: S4 Data — (PDF) [file pmed.1001916.s004.pdf]

S4 Data for McHugh et al., "A Molecular Host Response Assay to Discriminate Between Sepsis and Infection-Negative Systemic Inflammation in Critically Ill Patients: Discovery and Validation in Independent Cohorts"

ICU Admission Date as a Potential Confounding Variable

1. Objective

We investigated whether the date of admission to ICU was a confounding variable in using *SeptiCyte Lab* to discriminate between sepsis and infection negative systemic inflammation.

2. Methods

2.1 - We compared the probability density distributions of the *SeptiCyte Lab* score, for Validation Cohort 1 (cases and controls diagnosed with high confidence, n=59) vs. Validation Cohort 2 (cases randomly sampled across ICU admission dates, n=36). For this comparison the Welch two-sample t-test was used.

2.2 - We also compared the cumulative distributions of the *SeptiCyte Lab* score for Validation Cohorts 1 and 2. For this comparison the Kolmogorov-Smirnov (KS) test was used.

2.3 - Finally, we compared the cumulative distributions of the *SeptiCyte Lab* score for Validation Cohorts 1 and 2, after removing from Validation Cohort 2 six patients who had infection likelihoods of possible.

3. Results

3.1 - We compared the probability density distributions of the *SeptiCyte Lab* score for Validation Cohorts 1 and 2. The null hypothesis was that the means of the two score distributions were equal, as ascertained by the Welch two-sample t-test. Results of the comparison are shown in Figure 1. We found p-value = 0.111 implying that the null hypothesis cannot be rejected.

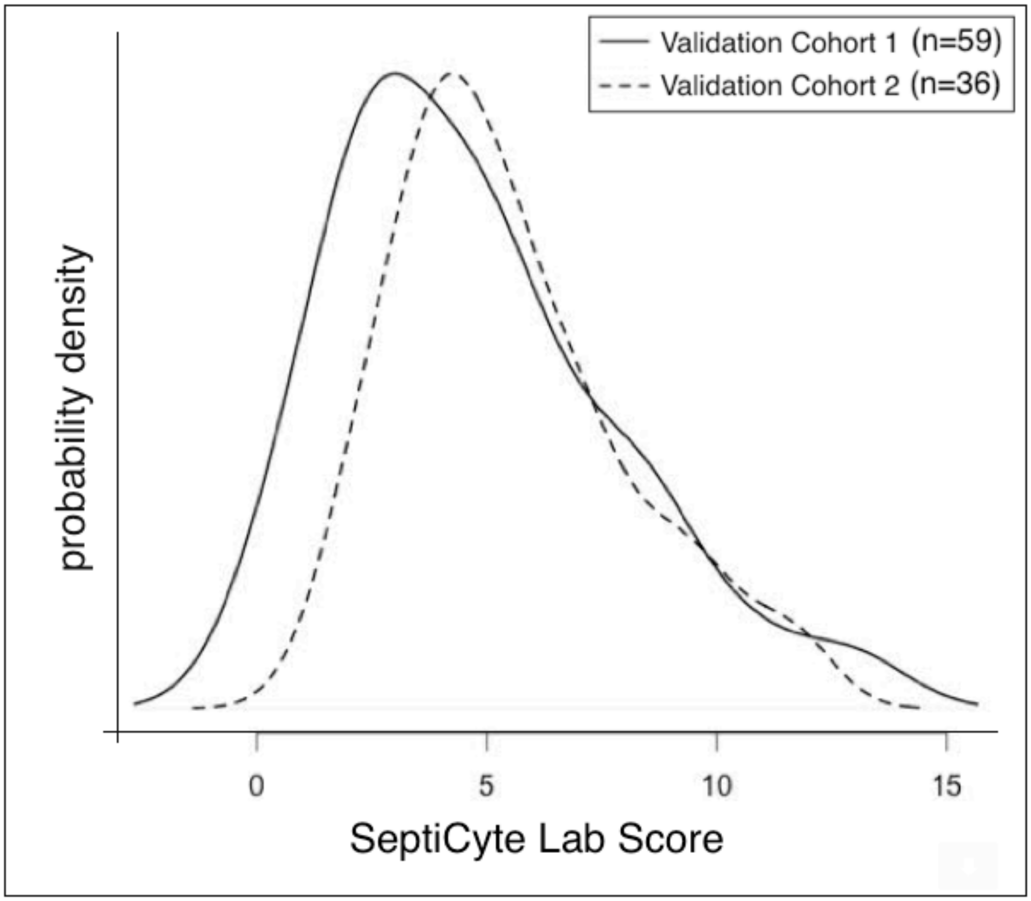

Figure 1: *Comparison of probability density distributions of SeptiCyte Lab score, for Validation Cohorts 1 and 2.*

3.2 - The KS test was then used to compare the cumulative *SeptiCyte Lab* score distributions between Validation Cohorts 1 and 2. Results are shown in **Figure 2**. The KS test gives the result D = 0.1954 for the maximal difference between the two cumulative distributions, with a corresponding p-value of 0.325. This indicates the two cumulative distributions are not significantly different.

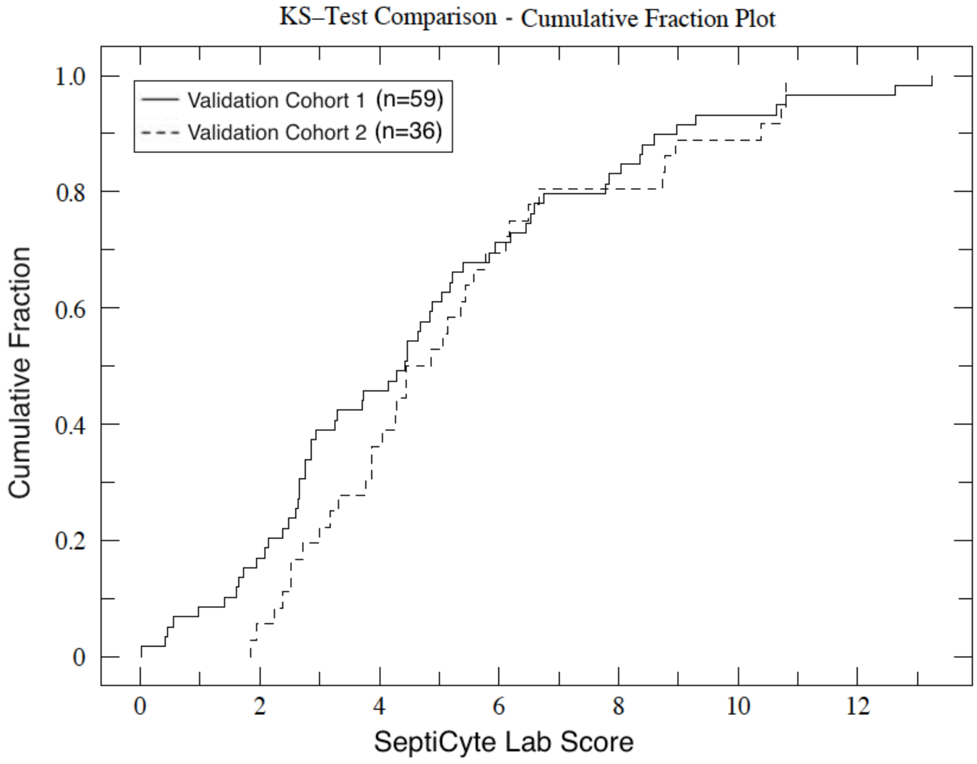

Figure 2: *Comparison of cumulative distributions of the SeptiCyte Lab score, for Validation Cohorts 1 and 2.*

3.3 - Finally, we removed from Validation Cohort 2 six patients having infection likelihoods of possible, and repeated the cumulative distribution analysis. Application of the KS test yields the result D = 0.1576 for the maximal difference between the two cumulative distributions with a corresponding p-value of 0.668. Thus removing the

patients who had infection likelihoods of possible caused the two distributions to move even closer together.

4. Summary

The present analysis has shown that Validation Cohorts 1 and 2 do not differ significantly, with respect to either the cumulative distribution of *SeptiCyte Lab* scores, or the probability distribution of *SeptiCyte Lab* scores. Thus there is no evidence to suggest that ICU admission date is a confounding variable.
