# Supplementary material for: A Molecular Host Response Assay to Discriminate Between Sepsis and Infection-Negative Systemic Inflammation in Critically Ill Patients: Discovery and Validation in Independent Cohorts
Source: PLoS Med. 2015 Dec 8;12(12):e1001916. doi: 10.1371/journal.pmed.1001916 (PMC4672921; doi:10.1371/journal.pmed.1001916)
Supplement: S5 Data — (PDF) [file pmed.1001916.s005.pdf]

S5 Data for McHugh et al., "A Molecular Host Response Assay to Discriminate Between Sepsis and Infection-Negative Systemic Inflammation in Critically Ill Patients: Discovery and Validation in Independent Cohorts"

Disease Severity as a Potential Confounding Variable

1. Objective

The objective was to investigate whether severity of disease, as measured by APACHE IV score or SOFA score, might be a confounding variable in using *SeptiCyte Lab* to discriminate between cases (sepsis) and controls (infection-negative systemic inflammation).

2. Method

Analysis #1: The entire patient pool (Validation Cohorts 1+2+3+4+5, excluding patients classified with an infection likelihood of possible; n=306) was subjected to ROC curve analysis, using either *SeptiCyte Lab* score, SOFA score, or APACHE IV score as a classifier.

Analysis #2: The entire patient pool (Validation Cohorts 1+2+3+4+5, excluding patients classified with an infection likelihood of possible; n=306) was stratified into "bins" on the basis of SOFA score or APACHE IV score. *SeptiCyte Lab* was then applied to the separate "bins" and the resultant ROC curves were pairwise compared.

3. Results

Analysis #1: Comparative ROC curve analyses on the pooled data (Validation Cohorts 1+2+3+4+5, excluding patients classified with an infection likelihood of possible; n=306) have been presented in **Figure 5, Panel A** in the main text.

Analysis #2: Results of ROC curve analysis on subsets of the combined dataset, stratified on either APACHE IV Score or SOFA Score, are presented below in **Figure 1** (APACHE IV) and **Figure 2** (SOFA).

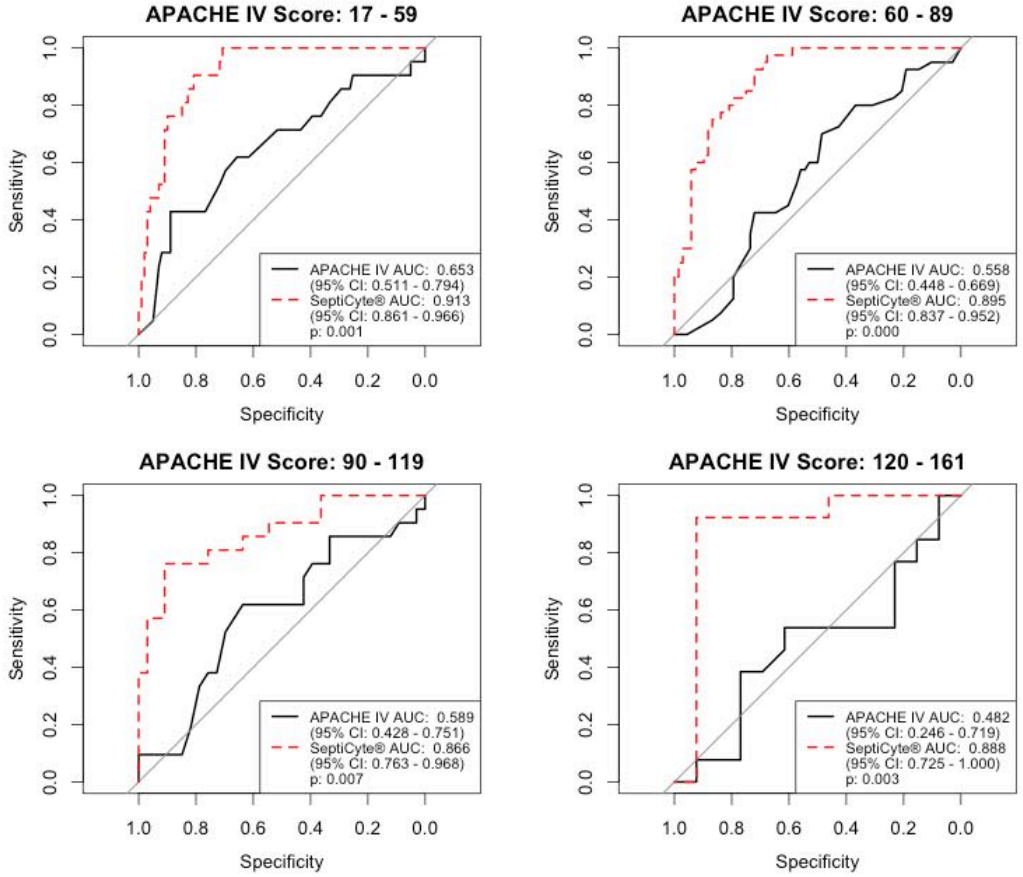

Figure 1: *ROC plots for SeptiCyte Lab, after stratifying patients by APACHE IV score*

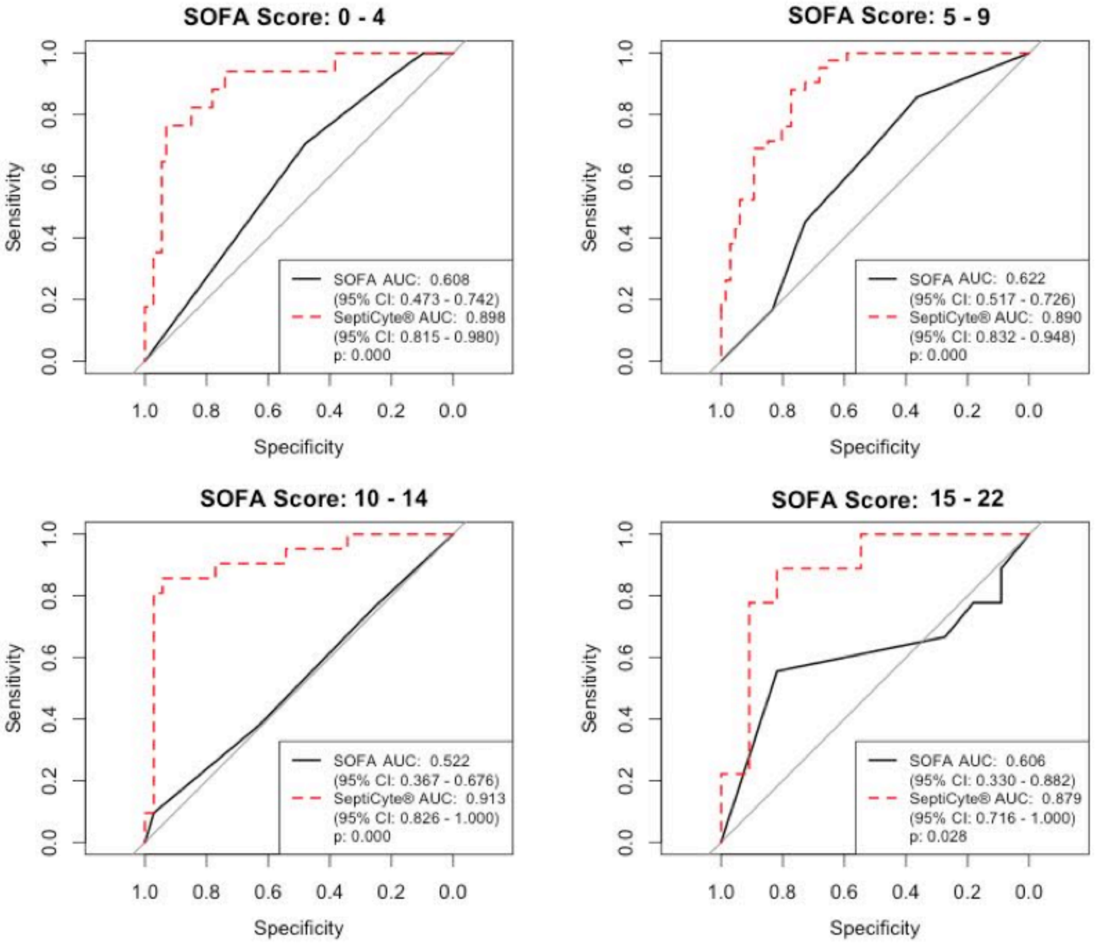

Figure 2: *ROC plots for SeptiCyte Lab, after stratifying patients by SOFA score*

The AUCs for the various bins are summarized in **Table 1** (APACHE IV Score) and **Table 2** (SOFA Score).

Table 1: *Stratification of patients into APACHE IV Bins, followed by ROC curve analysis using SeptiCyte Lab.*

Apache IV bin	Composition	AUC
17-59	99 controls, 21 cases	0.913 (95% CI: 0.861-0.966)
60-89	68 controls, 40 cases	0.895 (95% CI: 0.837-0.952)
90-119	33 controls, 21 cases	0.866 (95% CI: 0.763-0.968)
120-161	13 controls, 13 cases	0.888 (95% CI: 0.725-1.000)

Table 2*: Stratification of patients into SOFA Bins, followed by ROC curve analysis using SeptiCyte Lab.*

**SOFA bin**	**Composition**	**Septicyte AUC**
0-4	73 controls, 17 cases	0.898 (95% CI: 0.815-0.980)
5-9	66 controls, 42 cases	0.890 (95% CI: 0.832-0.948)
10-14	35 controls, 21 cases	0.913 (95% CI: 0.826-1.000)
15-22	11 controls, 9 cases	0.879 (95% CI: 0.716-1.000)

The p-values for comparison across bins are given in **Table 3** (for APACHE IV Score) and **Table 4** (for SOFA Score). We found no significant difference between *SeptiCyte Lab* ROC curves across the different "bins".

Table 3: *p values for pairwise comparison of SeptiCyte Lab ROC curves, after stratifying patients by APACHE IV score*

**APACHE IV Score**	**17-59**	**60-89**	**90-119**	**120-161**
**17-59**	1.00	0.30	0.56	0.23
**60-89**	0.30	1.00	0.76	0.57
**90-119**	0.56	0.76	1.00	0.47
**120-161**	0.23	0.57	0.47	1.00

Table 4: *p values for pairwise comparison of SeptiCyte Lab ROC curves, after stratifying patients by SOFA score*

**SOFA Score**	**0-4**	**5-9**	**10-14**	**15-22**
**0-4**	1.00	0.87	0.41	0.99
**5-9**	0.87	1.00	0.30	0.92
**10-14**	0.41	0.30	1.00	0.60
**15-22**	0.99	0.92	0.60	1.00
