# Supplementary material for: A Molecular Host Response Assay to Discriminate Between Sepsis and Infection-Negative Systemic Inflammation in Critically Ill Patients: Discovery and Validation in Independent Cohorts
Source: PLoS Med. 2015 Dec 8;12(12):e1001916. doi: 10.1371/journal.pmed.1001916 (PMC4672921; doi:10.1371/journal.pmed.1001916)
Supplement: S6 Data — (PDF) [file pmed.1001916.s006.pdf]

S6 Data for McHugh et al., "A Molecular Host Response Assay to Discriminate Between Sepsis and Infection-Negative Systemic Inflammation in Critically Ill Patients: Discovery and Validation in Independent Cohorts"

Analysis of Patients Classified as Infection Likelihood Possible

1. Introduction

A limitation of the present study – and indeed, of any analysis attempting to differentiate sepsis from infection-negative systemic inflammation – is the existence of patients classified by the reference method with an infection likelihood of possible. The true status of such patients - septic or not septic - cannot be determined with high certainty using any currently available methods.

Unambiguously diagnosed patients were selected for Validation Cohorts 1 and 3. Patients assessed with an infection likelihood of possible were deliberately excluded from these cohorts. It could be argued that this exclusion might introduce spectrum bias [1-4] into the estimates of *SeptiCyte Lab* performance based on the analysis of these cohorts. An alternative view is that patients assigned an infection likelihood of possible constitute a type of noise (classification noise) and therefore should be filtered out before conducting the analysis. We have investigated this possibility further.

2. Score Distribution Analysis

We investigated whether there was a significant difference between the distributions of *SeptiCyte Lab* scores for patients classified with an infection likelihood of possible, compared to all other patients in our study. Lack of a significant difference

between the distributions (i.e. random scatter of *SeptiCyte Lab* scores, for the patients assigned an infection likelihood of possible) would support the following hypothesis: that the assignment of an infection likelihood of possible constitutes a type of noise (classification noise).

We applied the Kolmogorov-Smirnov (KS) [1] test to the following two *SeptiCyte Lab* score distributions from Validation Cohorts 1+2+3+4+5: all samples having an infection likelihood of possible (N=37), versus all remaining samples (N=308). The results of this test are shown in **Figure 1**. The maximum difference between the two cumulative distributions, *D*, was found to be 0.1563 with a corresponding p-value of 0.366, which is insignificant at the $p<0.05$ level.

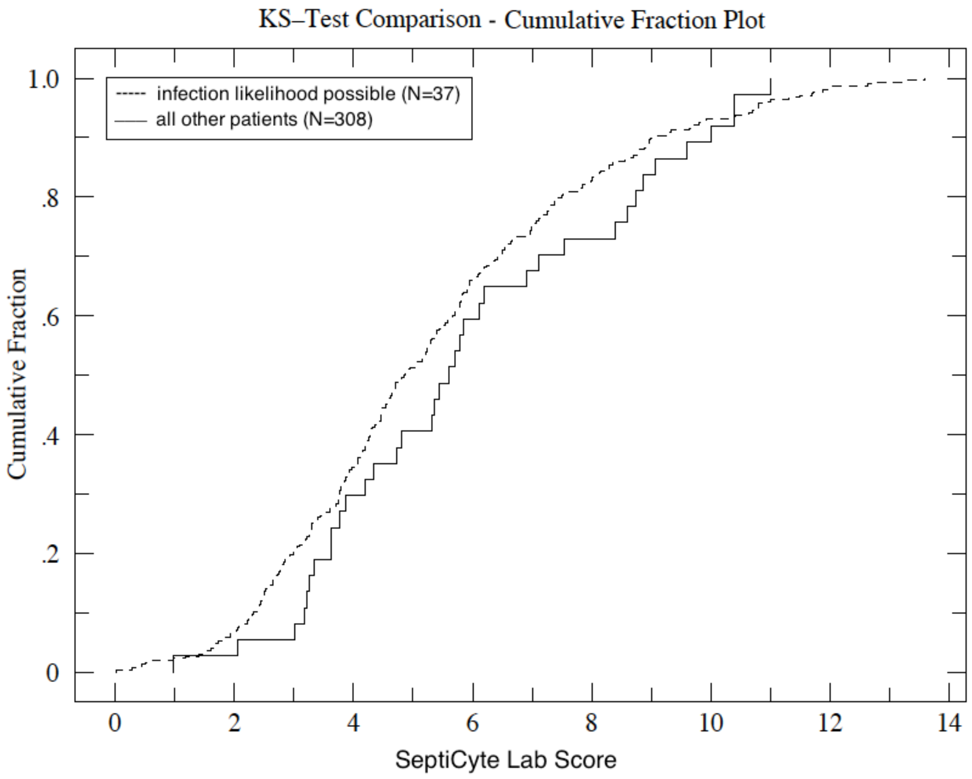

Figure 1: *Kolmogorov-Smirnov Test for Difference between SeptiCyte Lab Score Distributions*

[1] http://www.physics.csbsju.edu/cgi-bin/stats/KS-test

Additional analysis indicates that the distribution of *SeptiCyte Lab* scores for patients classified with an infection likelihood of possible is not different from a Gaussian (normal) distribution ($p=0.54$) with mean = 6.06 and SD = 2.67. This further supports the claim that these samples are distributed approximately at random across cases (sepsis) and controls (infection negative systemic inflammation).

3. Discussion

Noise is usually encountered as randomness in the same physical dimension as the signal of interest, which causes the signal to become degraded. However, in the present analysis, the signal (*SeptiCyte Lab* score, based on underlying Ct values) can be measured with a known precision. The uncertainty lies in the reference categorization.

A comparable situation is encountered in other disease areas where RNA expression profiles are used in a diagnostic capacity. For example, much effort has been devoted towards discovering RNA expression signatures that can correctly classify the primary origins of histologically ambiguous metastatic tumors. In attempting to assess the accuracy of such tests, the problem arises that certain reference samples may have a precisely quantifiable RNA expression signature, but may be of obscure bodily origin [5].

4. References

1. Ransohoff DF, Feinstein AR: Problems of Spectrum and Bias in Evaluating the Efficacy of Diagnostic Tests. N Engl J Med 1978; 299: 926-930

2. Lijmer JG, Mol BW, Heisterkamp S, Bonsel GJ, Prins MH, van der Meulen JH, Bossuyt PM: Empirical evidence of design-related bias in studies of diagnostic tests. J Am Med Assoc 1999; 282: 1061-1066. Erratum in: J Am Med Assoc 2000; 283:1963

3. Willis BH: Spectrum bias - why clinicians need to be cautious when applying diagnostic test studies. Fam Pract 2008; 25: 390-396

4. Mulherin SA, Miller WC: Spectrum bias or spectrum effect? Subgroup variation in diagnostic test evaluation. Ann Intern Med. 2002; 137: 598-602

5. Dumur CI, Fuller CE, Blevins TL, Schaum JC, Wilkinson DS, Garrett CT, Powers CN: Clinical verification of the performance of the pathwork tissue of origin test: utility and limitations. Am J Clin Pathol 2011; 136:924-933
