# Supplementary material for: A Molecular Host Response Assay to Discriminate Between Sepsis and Infection-Negative Systemic Inflammation in Critically Ill Patients: Discovery and Validation in Independent Cohorts
Source: PLoS Med. 2015 Dec 8;12(12):e1001916. doi: 10.1371/journal.pmed.1001916 (PMC4672921; doi:10.1371/journal.pmed.1001916)
Supplement: S7 Data — (PDF) [file pmed.1001916.s007.pdf]

S7 Data for McHugh et al., "A Molecular Host Response Assay to Discriminate Between Sepsis and Infection-Negative Systemic Inflammation in Critically Ill Patients: Discovery and Validation in Independent Cohorts"

Multi-Parameter Analysis

1. Background and Objective

Table 1 of Levy et al. (2003) ("Diagnostic Criteria for Sepsis") [1] presents a list of clinical parameters that an attending physician is likely to consider, in addition to documented or suspected infection, for diagnosing sepsis in a patient. This table includes procalcitonin (PCT), the most commonly used single protein biomarker for diagnosing sepsis. We hypothesized that some of these parameters might also be useful in discriminating sepsis from infection-negative systemic inflammation.

We compared the performance of *SeptiCyte Lab*, procalcitonin (PCT) and clinical parameters from Levy et al. (2003) for discriminating sepsis from infection-negative systemic inflammation in ICU patients. The clinical parameters were considered individually, in various logistic combinations, and in combination with PCT or *SeptiCyte Lab*.

2. Methods

<u>*Testing of Individual Parameters:*</u> Clinical parameters from the MARS dataset that were also represented in Table 1 of Levy et al. (2003) [1] were tested individually for significance in differentiating cases (sepsis) from controls (infection negative systemic inflammation). Each individual parameter was analyzed in two ways: (1) using all patients in Validation Cohorts 1+2+3+4+5 for which the parameter value had been recorded, excluding patients with an infection likelihood of possible (N ranged from 165 to 308); (2) using the largest set of patients (usually N=157) for which values were available for the best-performing combinations of clinical parameters listed in Table 2. Clinical parameters with individual values $p>0.05$ were excluded from further analysis.

<u>*Testing of Parameter Combinations:*</u> Logistic regression models were constructed by an iterative process, in which the best-performing clinical parameters were added into the models sequentially. Some models were constrained to exclude or include *SeptiCyte Lab* or PCT. All multi-parameter analyses used the largest set of patients (N=157) for which values were available for the best-performing clinical parameter combinations of Table 2. Classifier performance during the selection process was evaluated by receiver operating characteristic (ROC) curve analysis and ranked by area-under-curve (AUC). Once the final parameter combinations were obtained, the mean, median and 95% confidence intervals (CI) for AUC were computed by 50 resampling events. At each event, one half of the samples were randomly selected for training of a linear model and the remaining samples used for testing performance of the model.

3. Results

Performance of Individual Parameters: Nineteen of the clinical parameters listed in Levy et al. (2003) [1] were represented in the MARS study data, and had individual p values < 0.05 for separation of cases and controls. **Table 1** summarizes these clinical parameters, their individual AUC values (with the 95% CI bounds), and the number of patients used in each analysis.

Table 1: *Clinical Parameters Having p < 0.05 for Separation of Cases and Controls*

**Category of Variable**	**Levy et al. (2003) [1]**	**This study**	**All Available Patients for the Individual Parameter in Question**		**All Patients with Values Available for the Highest-AUC Parameter Combinations**	
			**Number of Patients Analyzed**	**Single-parameter AUC (95% CI)**	**Number of Patients Analyzed**	**Single-parameter AUC (95% CI)**
General	Fever (core temperature >38.3°C)	Maximum core temperature within 24 hours of ICU admission	289	0.62 (0.55 - 0.70)	157	0.62 (0.52 - 0.73)
General	Hypothermia (core temperature <36°C)	Minimum core temperature within 24 hours of ICU admission	288	0.57 (0.50 - 0.64)	156	0.56 (0.46 - 0.66)
General	Heart rate >90/min or >2 SD above the normal value for age	Maximum heart rate within 24 hours of ICU admission	308	0.67 (0.61 - 0.74)	157	0.71 (0.62 - 0.80)
General	Tachypnea	Maximum respiratory rate within 24 hours of ICU admission	308	0.60 (0.53 - 0.67)	157	0.58 (0.48 - 0.68)
General	Hyperglycemia (plasma glucose >120 mg/dL or 7.7 mmol/L) in the absence of diabetes	Maximum glucose concentration within 24 hours of ICU admission	307	0.55 (0.48 - 0.62)	157	0.59 (0.50 - 0.69)

Category of Variable	Levy et al. (2003) [1]	This study	All Available Patients for the Individual Parameter in Question		All Patients with Values Available for the Highest-AUC Parameter Combinations	
			Number of Patients Analyzed	Single-parameter AUC (95% CI)	Number of Patients Analyzed	Single-parameter AUC (95% CI)
		Diabetes (y/n)	308	0.56 (0.51 - 0.61)	157	0.57 (0.50 - 0.64)
Inflammatory	Leukocytosis (WBC count >12,000/uL)	Max WBC	299	0.52 (0.45 - 0.60)	156	0.51 (0.41 - 0.62)
Inflammatory	Leukopenia (WBC count <4000/uL)	Min WBC	296	0.55 (0.47 - 0.63)	156	0.56 (0.46 - 0.67)
Inflammatory	Plasma C-reactive protein (CRP) >2 SD above the normal value	CRP within 24 hours of ICU admission	165	0.85 (0.78 - 0.91)	131	0.86 (0.78 - 0.93)
Inflammatory	Plasma procalcitonin >2 SD above the normal value	log(PCT) within 24 hours of ICU admission	265	0.80 (0.74 - 0.86)	157	0.84 (0.77 - 0.91)
Inflammatory	Not used	SeptiCyte Lab Score	308	0.88 (0.84 - 0.92)	157	0.87 (0.82 - 0.93)
Hemodynamic	Arterial hypotension (SBP <90 mm Hg, MAP <70, or an SBP decrease >40 mm Hg in adults or <2SD below normal for age	Arterial blood pressure within 24 hours of ICU admission	304	0.60 (0.53 - 0.68)	155	0.66 (0.57 - 0.76)
Organ Dysfunction	Arterial hypoxemia (PaO_2/FIO_2 <300)	Minimum PaO_2/FIO_2 ratio within 24 hours of ICU admission	270	0.70 (0.62 - 0.77)	157	0.79 (0.70 - 0.87)

Category of Variable	Levy et al. (2003) [1]	This study	All Available Patients for the Individual Parameter in Question		All Patients with Values Available for the Highest-AUC Parameter Combinations	
			**Number of Patients Analyzed**	**Single-parameter AUC (95% CI)**	**Number of Patients Analyzed**	**Single-parameter AUC (95% CI)**
Tissue Perfusion	Hyperlactatemia (>1 mmol/L)	Lactate	259	0.51 (0.43 - 0.58)	123	0.54 (0.42 - 0.65)
Organ Dysfunction	Acute oliguria (urine output <0.5 mL/kg/hr or 45 mmol/L for at least 2 hrs)	Total urine output within 24 hours of ICU admission	308	0.60 (0.53 - 0.67)	157	0.63 (0.54 - 0.73)
Organ Dysfunction	Creatinine increase >0.5 mg/dL	Maximum creatinine within 24 hours of ICU admission	299	0.66 (0.60 - 0.73)	157	0.71 (0.62 - 0.80)
Organ Dysfunction	Coagulation abnormalities (INR >1.5 or a PTT >60 sec)	Minimum platelet count, within 24 hours of ICU admission	300	0.50 (0.43 - 0.58)	156	0.50 (0.39 - 0.60)
Organ Dysfunction	Hyperbilirubinemia (plasma total bilirubin >4 mg/dL or 70 mmol/L)	Maximum bilirubin within 24 hours of ICU admission	184	0.52 (0.42 - 0.62)	157	0.51 (0.41 - 0.62)

We developed and tested nine models each of which incorporated the SeptiScore, PCT, clinical parameters, or combinations thereof. Results are presented in **Table 2**. We intentionally avoided models with more than six parameters to minimize the possibility of over-fitting the data.

Table 2: *Summary of Tested Models*

**Model**	**Parameters**	**Number of Patients Analyzed**	**AUC**
1. PCT	log(PCT)	157	Mean: 0.84<br>Median: 0.84<br>95% CI: 0.76 - 0.92
2. SeptiCyte lab	SeptiScore	157	Mean: 0.88<br>Median: 0.88<br>95% CI: 0.81 - 0.93
3. PCT + SeptiCyte Lab	PCT<br>SeptiScore	157	Mean: 0.89<br>Median: 0.89<br>95% CI: 0.82-0.95
4. Top 3 clinical parameters (excluding PCT and SeptiCyte Lab)	PaO_2/FIO_2.ratio.Min.24h<br>Bilirubin_Max_24h<br>Urine.output.Sum.24h	157	Mean: 0.78<br>Median: 0.78<br>95% CI: 0.71-0.88
5. Top 5 clinical parameters (excluding PCT and SeptiCyte Lab)	PaO_2/FIO_2.ratio.Min.24h<br>Bilirubin_Max_24h<br>Urine.output.Sum.24h<br>Glucose.concentration<br>HR.Max.24h	157	Mean: 0.81<br>Median: 0.81<br>95% CI: 0.71-0.89
6. Top 5 parameters + PCT	PaO_2/FIO_2.ratio.Min.24h<br>Bilirubin_Max_24h<br>Urine.output.Sum.24h<br>Glucose.concentration<br>HR.Max.24h<br>PCT	157	Mean: 0.84<br>Median: 0.84<br>95% CI: 0.75-0.91
7. Top 5 parameters + SeptiCyte Lab	PaO_2/FIO_2.ratio.Min.24h<br>Bilirubin_Max_24h<br>Urine.output.Sum.24h<br>Glucose.concentration<br>HR.Max.24h<br>SeptiScore	157	Mean: 0.87<br>Median: 0.87<br>95% CI: 0.79-0.93
8. PCT + next best 4 parameters (excluding SeptiCyte Lab)	PCT<br>PaO_2/FIO_2.ratio.Min.24h<br>Bilirubin_Max_24h<br>HR.Max.24h	157	Mean: 0.85<br>Median: 0.85<br>95% CI: 0.76-0.91

Model	Parameters	Number of Patients Analyzed	AUC
	Urine.output.Sum.24h		
9. SeptiCyte Lab + next best 4 parameters (with PCT allowed as possibility)	SeptiScore<br>PCT<br>PaO_2/FIO_2.ratio.Min.24h<br>Bilirubin_Max_24h<br>Urine.output.Sum.24h	157	Mean: 0.89<br>Median: 0.90<br>95% CI: 0.83-0.95

Note that we have not included CRP in any of the logistic models, for the following reasons:

1. We were able to obtain CRP data for only 173/345 (50.1%) of the patients in the Validation Cohorts. It appears that, in connection with the diagnosis of sepsis, critical care physicians either order CRP infrequently or fail to record the CRP data in electronic case report forms.
2. The retrospective patient evaluation process used by the MARS ICU physicians for assigning infection likelihood was based, in part, on the measured CRP values available at that time. Any attempt to estimate the diagnostic value of CRP in our Validation Cohorts would necessarily use retrospective assignment of infection likelihoods as the reference method. Thus, a logical circularity would be introduced, and performance evaluation of CRP would be fundamentally biased for this reason.
3. CRP has a different clinical response profile and utility than SeptiCyte Lab. C-reactive protein is an acute-phase protein, the concentration of which is known to increase as a consequence of rising IL-6 levels in response to a wide range of conditions: not only bacterial, viral, or fungal infections, but also acute and chronic inflammatory conditions such as rheumatoid arthritis [2-4], psoriasis [5],

inflammatory bowel disease [6], asthma [7,8], malignancies [9,10], tissue injuries [11,12], and burns [13]. In addition, CRP levels are also affected by diet [14,15], exercise [16], sleep disturbances [17,18], depression [19], and aging [20].

4. Discussion

When the N=157 patient dataset held in common by all classifiers was analyzed, *SeptiCyte Lab* (AUC 0.88; 95% CI: 0.81-0.93) was found to outperform both PCT (AUC 0.84; 95% CI: 0.76-0.92; $p < 8.3 \times 10^{-16}$) and the best combination of five clinical parameters (mean AUC 0.81; 95% CI: 0.71-0.89; $p < 2.2 \times 10^{-16}$), for discriminating cases from controls. When *SeptiCyte Lab* was added to PCT, an increase in AUC was observed (AUC = 0.89; 95% CI 0.82-0.95). Also, when *SeptiCyte Lab* was added to the best combination of five clinical parameters, an increase in AUC was observed (AUC = 0.87; 95% CI: 0.79-0.93). Conversely, when PCT and the best three clinical parameters were added to *SeptiCyte Lab*, a slight increase in AUC was observed (AUC = 0.89-0.90; 95% CI: 0.83-0.95). Thus, *SeptiCyte Lab* adds diagnostic information to what is available either from PCT, or from the most discriminating combination of clinical parameters readily available within 24 hours of ICU admission.

We note that the clinical parameters in Table 1 of Levy et al. (2003) were not intended for the differential diagnosis of patients classified in our study as cases (sepsis) or controls (infection-negative systemic inflammation). Rather, the Levy parameters were intended for use in cases of probable or proven infection to aid in diagnosing whether the patients in question, in response to the infection, are septic or not.

We note also that the attending ICU physicians would have considered all available clinical parameters in their retrospective assessment of infection likelihood. Thus, because the clinical parameters of Table 1 were used both in the initial assessment of infection likelihood, and also in the logistic regression modeling, there is some degree of logical circularity embedded in the present analysis. This is expected to lead to inflated values for the AUC observed for the clinical parameter combinations.

5. Conclusion

SeptiCyte Lab outperformed both PCT and the best available five- clinical parameter combination in discriminating cases from controls. Thus *SeptiCyte Lab* adds diagnostic information to what is available either from PCT or from the clinical parameters that are readily available within 24 hours of ICU admission.

6. References

1. Levy MM, Fink MP, Marshall JC, Abraham E, Angus D, Cook D, Cohen J, Opal SM, Vincent JL, Ramsay G: 2001 SCCM/ESICM/ACCP/ATS/SIS International Sepsis Definitions Conference. Crit Care Med 2003; 31: 1250-1256

2. Dessein PH, Joffe BI, Stanwix AE: High sensitivity C-reactive protein as a disease activity marker in rheumatoid arthritis. J Rheumatol. 2004; 31: 1095-1097. PMID: 15170920

3. Graf J, Scherzer R, Grunfeld C, Imboden J: Levels of C-reactive protein associated with high and very high cardiovascular risk are prevalent in patients with rheumatoid arthritis. PLoS One. 2009; 4:e6242. PMID: 19606218

4. Singh HV, Shrivastava AK, Raizada A, Singh SK, Pandey A, Singh N, Yadav D, Sharma H: Atherogenic lipid profile and high sensitive C-reactive protein in patients with rheumatoid arthritis. Clin Biochem. 2013; 46:1007-1012. PMID: 23578742

5. Dowlatshahi EA, van der Voort EA, Arends LR, Nijsten T: Markers of systemic inflammation in psoriasis: a systematic review and meta-analysis. Br J Dermatol. 2013; 169: 266-282. PMID: 23550658

6. Sands BE: Biomarkers of Inflammation in Inflammatory Bowel Disease. Gastroenterology. 2015; Jul 9. pii: S0016-5085(15)00938-5. doi: 10.1053/j.gastro.2015.07.003. [epub ahead of print] PMID: 26166315

7. Razi E, Ehteram H, Akbari H, Chavoshi V, Razi A: Evaluation of high-sensitivity C-reactive protein in acute asthma. Tanaffos. 2012; 11: 32-37. PMID: 25191398

8. Shimoda T, Obase Y, Kishikawa R, Iwanaga T: Serum high-sensitivity C-reactive protein can be an airway inflammation predictor in bronchial asthma. Allergy Asthma Proc. 2015; 36:e23-28. PMID: 25715235

9. Allin KH, Nordestgaard BG: Elevated C-reactive protein in the diagnosis, prognosis, and cause of cancer. Crit Revs Clin Lab Sciences 2011; 48: 155–170. doi:10.3109/10408363.2011.599831. PMID 22035340

10. Wang J, Ma R, Sharma A, He M, Xue J, Wu J, Dun B, Li G, Wang X, Ji M, She JX, Tang J: Inflammatory serum proteins are severely altered in metastatic gastric adenocarcinoma patients from the Chinese population. PLoS One 2015; 10:e0123985. doi: 10.1371/journal.pone.0123985. PMID: 25884401

11. Pritchett JW: C-reactive protein levels determine the severity of soft-tissue injuries. Am J Orthop 1996; 25:759-761. PMID: 8959256

12. Haslauer CM, Proffen BL, Johnson VM, Hill A, Murray MM: Gene expression of catabolic inflammatory cytokines peak before anabolic inflammatory cytokines after ACL injury in a preclinical model. J Inflamm (Lond). 2014; 11: 34. PMID: 25400511

13. van de Goot F, Krijnen PA, Begieneman MP, Ulrich MM, Middelkoop E, Niessen HW: Acute inflammation is persistent locally in burn wounds: a pivotal role for complement and C-reactive protein. J Burn Care Res. 2009; 30:274-280. PMID: 19165117

14. Lopez-Garcia E, Schulze MB, Meigs JB, Manson JE, Rifai N, Stampfer MJ et al.: Consumption of trans fatty acids is related to plasma biomarkers of inflammation and endothelial dysfunction". J Nutrition 2005; 135: 562–566. PMID 15735094

15. St-Onge MP, Zhang S, Darnell B, Allison DB: Baseline serum C-reactive protein is associated with lipid responses to low-fat and high-polyunsaturated fat diets". J Nutrition 2009; 139: 680–683. PMID 19297430

16. Swardfager W, Herrmann N, Cornish S, Mazereeuw G, Marzolini S, Sham L et al.: Exercise intervention and inflammatory markers in coronary artery disease: a meta-analysis. American Heart Journal 2012; 163: 666–676. PMID 22520533

17. Nadeem R, Molnar J, Madbouly EM, Nida M, Aggarwal S, Sajid H, Naseem J, Loomba R: Serum inflammatory markers in obstructive sleep apnea: a meta-analysis. J Clin Sleep Med. 2013; 9:1003-1012. PMID: 24127144

18. Irwin MR, Olmstead R, Carroll JE: Sleep Disturbance, Sleep Duration, and Inflammation: A Systematic Review and Meta-Analysis of Cohort Studies and Experimental Sleep Deprivation. Biol Psychiatry. 2015; Jun 1. pii: S0006-3223(15)00437-0. doi: 10.1016/j.biopsych.2015.05.014.PMID: 26140821

19. Haapakoski R, Mathieu J, Ebmeier KP, Alenius H, Kivimäki M: Cumulative meta-analysis of interleukins 6 and 1β, tumour necrosis factor α and C-reactive protein in patients with major depressive disorder. Brain Behav Immun. 2015; Jun 9. pii: S0889-1591(15)00152-X. doi: 10.1016/j.bbi.2015.06.001. PMID: 26065825

20. Frasca D, Blomberg BB. Inflammaging decreases adaptive and innate immune responses in mice and humans. Biogerontology. 2015 Apr 29. [epub ahead of print] PMID: 25921609
