# Supplementary material for: A Molecular Host Response Assay to Discriminate Between Sepsis and Infection-Negative Systemic Inflammation in Critically Ill Patients: Discovery and Validation in Independent Cohorts
Source: PLoS Med. 2015 Dec 8;12(12):e1001916. doi: 10.1371/journal.pmed.1001916 (PMC4672921; doi:10.1371/journal.pmed.1001916)
Supplement: S1 Text — (PDF) [file pmed.1001916.s008.pdf]

S1 Text for McHugh et al., "A Molecular Host Response Assay to Discriminate Between Sepsis and Infection-Negative Systemic Inflammation in Critically Ill Patients: Discovery and Validation in Independent Cohorts"

Summaries of Protocols for the GCP-1 and RTT Studies (Discovery Phase) and the MARS Study (Validation Phase)

Supporting Information S1 consists of three parts.

Part 1: GCP-1 Summary

Part 2: RTT Summary

Part 3: MARS Summary

Within each part, we indicate: (1) the specific hypotheses that were tested; (2) the analytical methods that were used in testing the hypotheses; and (3) the analyses that were performed. When reported analyses differ from those that were planned, we provide transparent explanations for any differences that might affect the reliability of the study's results.

Part 1: GCP-1 trial

Protocol Identification: ATH-100-01, "Development and Validation of a Novel Molecular Assay for Definitive Diagnosis of Septicaemia in Patients Presenting with a Non-specific Immuno-inflammatory Response"

Web link: none available.

Protocol Authors:
Dr Allison Sutherland BSc (Hons) PhD
Prof Deon Venter MB ChB PhD FRCPA MBA
Dr Mervyn Thomas BSc MSc Phil D

Version Dates:
Version 1: 12-Feb-2008
Version 2: 14-Jul-2008
Version 3: 28-Jul-2009

Background: Prior to the GCP-1 trial, a pre-clinical study investigating gene expression profiles in an equine model of sepsis (non-surgical induced endotoxemia) identified 161 genes that were significantly elevated in comparison to non-treated healthy controls, including the same animals that underwent sham induction. A subsequent pilot study in humans yielded a list of genes that appeared to discriminate between sepsis cases and controls; 120 genes on this list overlapped with the 161 genes identified previously in the equine study.

Hypothesis (as stated in Study Protocol): that the set of previously identified molecular biomarkers for sepsis can be effectively used in the clinical setting to expedite diagnosis and improve clinical treatment plans.[1]

Proposed Analytical Methods: collection of demographic information, vital signs (blood pressure, heart rate, respiratory rate, oxygen saturation, temperature), haematology, clinical chemistry, and microbial status to be recorded in case report forms. Blood specimens to be collected for molecular profiling, flow cytometry analysis, and PCT measurements. Urine specimens to be collected for proteomics analysis.

Planned Cohort Sizes: 85 subjects total, consisting of 35 control surgical patients, 25 septicaemia patients, and 25 patients with SIRS.

Inclusion Criteria, Surgical Controls: age > 18 years, body mass index < 40, legally capable of giving informed consent.

---

[1] Note: the present work has progressed well beyond this early hypothesis, to encompass the search for additional biomarkers and biomarker combinations having enhanced diagnostic power.

Inclusion Criteria, SIRS and Sepsis Patients: age > 18 years, body mass index < 40, two or more SIRS criteria; patient or next of kin legally capable of providing informed consent

Exclusion Criteria: chronic systemic immune-inflammatory disorders including SLE [systemic lupus erythematosus], Crohn's disease, IDDM [insulin-dependent diabetes mellitus]; are transplant recipients; are currently receiving chemotherapy treatment for cancer; known HCV(+) or HIV(+); Aboriginal or Torres Strait Islander who are likely to have rheumatic heart disease.

Study Sites: Wesley Hospital; Mater Adult Public and Private Hospitals (both in metropolitan region of Brisbane, Australia).

Analyses Actually Performed: Microarray data from the GCP-1 trial and RTT trial (described below) were reanalyzed using an improved bioinformatics analysis approach as described in the Discovery section of the present manuscript. Other analyses that were initially proposed in the GCP-1 study protocol (flow cytometry analysis; proteomics analysis) were not included in the present study.

Rationale for Deviation to Study Plan: (1) A decision was made to restrict the present study only to the discovery and validation of molecular classifiers based on blood RNA profiling. (2) Greater statistical power could be obtained by increasing the number of samples through combining this study (GCP-1) with a second study (RTT) described below. (3) Bioinformatics methods of greater sophistication were used, leading to discovery of the 4-gene *SeptiCyte Lab* signature, which has greater diagnostic power with fewer genes, compared to previously identified signatures.

GCP-1 Protocol Synopsis: attached (13 pages).

STUDY TITLE: Development and Validation of a Novel Molecular Assay for Definitive Diagnosis of Septicaemia in Patients Presenting with a Non-specific Immuno-inflammatory Response

PROTOCOL NUMBER: ATH-100-01

INVESTIGATIONAL PRODUCT: 100 (novel diagnostic qRT-PCR assay)

INDICATION: Diagnosis of septicaemia

DEVELOPMENT PHASE: Phase I

PROTOCOL AUTHORS: Dr Allison Sutherland BSc (Hons) PhD

Prof Deon Venter MB ChB PhD FRCPA

Dr Mervyn Thomas BSc MSc Phil D

SPONSOR: Athlomics Pty Limited

PO Box 1448

Toowong Q 4066

Australia

MEDICAL DIRECTOR: Prof Deon Venter MB ChB PhD FRCPA

Co-Director of Pathology

Mater Health Services

Raymond Terrace,

South Brisbane Qld 4101

CONFIDENTIAL

Property of Athlomics Pty Ltd

Information contained in this protocol should not be disclosed, other than to those directly involved in the execution or ethical review of the study, without written authorization from Athlomics Pty Ltd. It is however, permissible to provide information to a patient in order to obtain consent.

This study will be conducted in accordance to the principles of Good Clinical Practice (GCP) as described by the International Conference on Harmonization guidelines, including the archiving of essential documents. Guidelines in the National Statement on Ethical Conduct in Human Research ratified by the National Health & Medical Council (NHMRC) and Australian Vice Chancellors' Committee (AVCC) will be observed.

All information concerning the study assay developed by Athlomics Pty Ltd in connection with this clinical trial and not previously published is considered confidential and proprietary information. This information includes the clinical protocol, informed consent forms and case report forms (CRF).

Site Investigators are obliged to provide Athlomics Pty Ltd with complete study results and all data derived from this study. Only Athlomics Pty Ltd may enable information obtained during this study available to be provided to the participating physicians and to the regulatory agencies, as required by law.

Protocol Approval

Authorised by:

Prof Deon Venter<br>Medical Director,<br>Athlomics Pty Ltd	...................................<br>Date 25/7/09
Dr Roz Brandon<br>CEO,<br>Athlomics Pty Ltd	...................................<br>Date ....
Dr Richard Brandon<br>Research Director,<br>Athlomics Pty Ltd	...................................<br>Date ....
Dr Mervyn Thomas<br>Biostatistician,<br>Athlomics Pty Ltd	...................................<br>Date ....

Protocol Approval

Authorised by:

Prof Deon Venter<br>Medical Director,<br>Athlomics Pty Ltd	....<br>Date ....
Dr Roz Brandon<br>CEO,<br>Athlomics Pty Ltd	....<br>Date 28/7/09
Dr Richard Brandon<br>Research Director,<br>Athlomics Pty Ltd	....<br>Date ....
Dr Mervyn Thomas<br>Biostatistician,<br>Athlomics Pty Ltd	....<br>Date ....

Protocol Approval

Authorised by:

Prof Deon Venter<br>Medical Director,<br>Athlomics Pty Ltd	....<br>Date ....
Dr Roz Brandon<br>CEO,<br>Athlomics Pty Ltd	....<br>Date ....
Dr Richard Brandon<br>Research Director,<br>Athlomics Pty Ltd	....<br>Date 29th July 2009
Dr Mervyn Thomas<br>Biostatistician,<br>Athlomics Pty Ltd	....<br>Date ....

Protocol Approval

Authorised by:

Prof Deon Venter<br>Medical Director,<br>Athlomics Pty Ltd	....<br>Date ....
Dr Roz Brandon<br>CEO,<br>Athlomics Pty Ltd	....<br>Date ....
Dr Richard Brandon<br>Research Director,<br>Athlomics Pty Ltd	....<br>Date ....
Dr Mervyn Thomas<br>Biostatistician,<br>Athlomics Pty Ltd	....<br>Date 29/9/2009

Amendment History

Version #2 (dated 14 July 2008) includes the following amendments:

- Clarification as to when patients will be stratified into the SIRS and Sepsis cohort
- Increase the number of patients recruited into the surgical control cohort from 25 to 35.
- Clarification regarding the collection of specimens for microbiology culture for both the control and experimental groups
- Clarification that intra-operative medication (including blood and fluid products) will not be captured as concomitant medications in the CRF
- Clarification regarding the description of Serious Adverse Events and how they will be followed-up as well as the inclusion of causality definitions for adverse events
- Clarification on the process of informed consent
- Clarification that all study data captured in the CRF as well as source documentation will be archived for 15 years after the completion of the trial
- Clarification that both Mater Private and Adult Public Hospitals will be participating in the study. Additional study sites may participate in the study as required.
- Clarification regarding sample collection and preparation (Appendices B & C)
- Clarification of retrospective confirmation of assignment to the SIRS or Sepsis cohorts. As a result of attrition of some patients from the study who are retrospectively found to have neither SIRS nor septicaemia, it is expected that there may be up to a 30 % rate of attrition. Therefore, up to approximately 100 patients may initially have to be enrolled into this study, prior to the retrospective SIRS or Sepsis assignment.

Version #3 (dated 28 July 2009) includes the following amendment:

- Modification of the respiratory parameters and haematology results that are used to define presenting signs and symptoms of SIRS and sepsis in the Inclusion Criteria for the SIRS and Sepsis Cohort, and based on guidelines established by the American College of Chest Physicians/Society of Critical Care Medicine (1992). Specifically, on pages 8 and 22 arterial partial pressure ($PaCO_2$) should be less than 4.3 kilopascals (kPa) or less than 32 millimetres of mercury (mmHg). Additionally, reference to "leukocytopenia ($\leq 1.0 \times 10^9$/L) or leukocytosis ($>4.0 \times 10^9$/L)" on pages 8 and 22 has been amended to a white blood cell count of less than 4,000 cells/mm^3 (4×10^9 cells/L) or a white blood cell count greater than 12,000 cells/mm^3 (12×10^9 cells/L); or presence of greater than 10% immature neutrophils (bands forms).
- Clarification regarding sample handling and preparation for the SIRS and Sepsis Cohorts at the RBWH site (Appendix C; page 39)

1.0 PROTOCOL SYNOPSIS FOR ATH-100-01

TITLE

Development and Validation of a Novel Molecular Assay for the Definitive Diagnosis of Septicaemia in Patients Presenting with a Non-specific Immuno-inflammatory Response

PROTOCOL NUMBER: ATH-100-01

RATIONALE

Sepsis is a specific systemic inflammatory response to either gram positive or gram negative bacterial or fungal infection and is a common cause of morbidity and mortality in the intensive care unit; where sepsis can escalate to septic shock and multiple organ dysfunction syndrome. During the early pathogenesis of septicaemia, diagnosis is often delayed owing to the relative slowness of laboratory bacterial and fungal culture methods and impaired due to other confounding aetiologies. Thus, there is a clinical necessity for an assay that can expedite the early diagnosis of septicaemia. Recent developments in biomolecular technologies using quantitative reverse transcriptase polymerase chain reaction (qRT-PCR) enable gene expression patterns to be translated into diagnostic pathology profiles and have the capacity to improve acute clinical management.

A pre-clinical study investigating gene expression profiles in an equine model of sepsis has identified 161 genes that were significantly elevated in comparison with a non-treated control. A subsequent pilot study performed at the Mater Hospitals campus in Brisbane in 2007, using GeneChip® -based genomics technologies yielded a list of genes in sepsis cases versus controls that matched the equine genes to a very high degree. One hundred and twenty of the 161 genes identified in the horse septicaemia model were seen in the human pilot study - the probability of these 140 genes being identified by chance from the 40,000 sequences on the GeneChip® is approximately 0.5 x $^{10-11}$ These findings suggest robustness of these gene expression markers for clinical diagnosis of septicaemia.

OBJECTIVES

Primary Objectives: To validate a novel molecular assay for the definitive diagnosis of septicaemia using an *a priori* panel of biomarkers obtained from an equine model of sepsis, confirmed by a pilot study in humans. The robustness of the assay will be tested; sensitivity and specificity will be determined after comparing gene expression profiles between a cohort of patients with systemic inflammatory response syndrome (SIRS), a cohort of patients with sepsis and a cohort of peri-operative surgical patients who are electively admitted to the ICU – these latter patients exhibit the manifestations of general somatic stress, a wounding response, but are infection-free.

Secondary Objectives: To assess safety parameters and to establish whether gene expression profiles for septicaemia are concordant with the corresponding cell surface and secreted protein markers, using flow cytometric methods.

STUDY PERIOD: 6 months

DEVELOPMENT STATUS: Phase I

METHODOLOGY

This is a multi-centre diagnostic trial to evaluate whether a set of previously identified molecular biomarkers for sepsis can be effectively used in the clinical setting to expedite diagnosis and improve clinical treatment plans. This Phase I study will assess results from 85 subjects and includes a cohort of 35 control surgical patients and 50 disease patients, of which 25 have septicaemia and 25 have SIRS.

A definitive diagnosis of SIRS and septicaemia may not be known at the time patients participate in this clinical study. A retrospective confirmation of diagnosis and assignment to the SIRS or Sepsis cohorts will be made. It is expected that there may be up to a 30 % rate of attrition of patients that end up being excluded from the analysis. Owing to this attrition rate, it is anticipated that we may have to recruit approximately 100 patients into this study initially, in order to achieve cohorts of 25 SIRS and 25 septicaemia patients. Data collected from participants who are not diagnosed with SIRS or septicaemia will only be assessed for frequency of adverse events. Participants (or next of kin) will be informed at the time of consent that their study research samples may not be analysed if they are not confirmed in the SIRS or Sepsis cohorts. These research samples will be destroyed.

Following a review of the inclusion and exclusion criteria, patients (or next of kin) who sign an informed consent form (ICF) and meet all of entry criteria will be offered the opportunity to participate in this study. The study will include three cohorts of patients. Patients who are enrolled into the control surgical cohort will be identified from the surgical lists and consented prior to their procedure for which there is a planned ICU admission. However, clinical data collection and blood specimens will not be collected until after surgery during their post-operative stay in ICU. Patients who have clinical signs and/or symptoms of sepsis or SIRS will be consented and enrolled into the study as soon as possible after admission, and ideally within 24 hours. The final assessment of SIRS or sepsis will be made by each site physician Investigator as information becomes available. Hence, assignment to the SIRS or sepsis cohorts may be retrospective or may be re-assigned following final review of the clinical data.

Demography, vital signs measurements (blood pressure, heart rate, respiratory rate, oxygen saturation, temperature), blood specimens collected and current results obtained from patient charts for haematology and clinical chemistry, as well as microbial status will be recorded in the case report form (CRF). In addition, 6-8 further blood specimens (variable between cohorts, however a maximum combined total for all 8 = 30ml) will be collected for molecular profiling, cytomic and other biomarker investigations. A urine specimen (50ml) will also be collected for proteomics development.

Blood specimens (included in the total of 30ml above) for gene expression analysis will be collected into vacutainer Paxgene tubes; samples for flow cytometry will be collected into EDTA tubes and samples for further biomarker investigations will be collected into plain tubes not containing anticoagulant. RNA isolation will be performed using a standard operating procedure (SOP_RNA Isolation). Once the total RNA has been extracted from the blood specimen, this material will be reverse-transcribed to create double-stranded complementary DNA (cDNA) and then amplified with specific primers using quantitative multiplex PCR (SOP_cDNA synthesis and qRT PCR). Primers will be developed to enable gene expression of specific molecular biomarkers identified from pre-clinical studies that correlate with septicaemia. Furthermore, cRNA will be synthesised for Genechip® hybridization (SOP_cRNA and Genechip® Hybridization) to better delineate differences in gene expression between sepsis and SIRS. Cell surface protein markers will be profiled using flow cytometry and compared against outcomes from the molecular assay. Flow cytometry methods will follow standard operating procedures used in daily practice at Mater Pathology. It should be noted that the maximum amount of blood to be collected from participants for this study will be 30 ml. Control surgical patients will be required to have blood collected (5 ml) for blood culture microbiology assessment, in order to exclude, as far as possible, the possibility of sepsis in these control subjects. As a result the surgical cohort patients will only have blood collected for 4 Paxgene tubes.

Adverse events will be collected for 12 hours after study blood specimens are withdrawn and concomitant medications will be collected from all patients at the time of study blood sampling and will also include medications administered for the 24 hours preceding this time.

NUMBERS OF SUBJECTS

This study will be comprised of three cohorts of patients and will include: A cohort of 25 patients with clinical signs and symptoms consistent with SIRS; a cohort of 25 patients with the clinical signs and symptoms consistent with septicaemia and a control cohort of 35 post-operative surgical patients. A total of 85 patients will ultimately be analysed at the molecular level. Since it is expected that there may be up to a 30 % rate of attrition of patients excluded from the analysis by not having a diagnosis of sepsis or SIRS confirmed, approximately 100 patients may be initially recruited and participate in the study in order to achieve the required 85 individuals for molecular analysis.

MAIN CRITERIA FOR INCLUSION

Control Surgical Cohort: patients admitted for planned major surgery who will have a brief elective peri-operative stay in the ICU

1. Aged over 18 years
2. Body Mass Index <40
3. The patient is fluent in English (both written and spoken) and capable of providing informed consent to participate in the study

SIRS and Sepsis Cohort: patients presenting with clinical signs and symptoms of SIRS or Sepsis:

1. Aged over 18 years
2. Body Mass Index < 40
3. A variable combination of clinical conditions including two or more of the following within the last 24 hours: temperature >38°C or <36°C; heart rate >90 beats/min; respiratory rate >20 breathes/min or a $PaCO_2$ of <4.3kPa (<32 mm Hg); and evidence of a white blood cell count <4,000 cells/mm^3 (<4 x 10^9 cells/L) or >12,000 cells/mm^3 (>12 x 10^9 cells/L) or >10% immature neutrophils (band forms).
4. The patient and senior next of kin or legally-accepted representative are fluent in English (both written and spoken) and the patient or senior next of kin or legally-accepted representative are capable of providing informed consent to participate in the study

A definitive diagnosis of SIRS and septicaemia may not be known at the time patients participate in this clinical study. A retrospective confirmation of diagnosis and assignment to the SIRS or Sepsis cohorts will be made.

EXPERIMENTAL ASSAYS:

Genechips will be used as a high through-put analysis system to focus the selection of key gene expression markers in septicaemia and SIRS. Previous studies have isolated an *a priori* of 140 genes from an initial 57,000 that were significantly elevated in an equine model of sepsis compared with control. These Genechip investigations will be continued in this Phase I study to establish a molecular profile consistent with SIRS.

Following isolation of approximately a dozen genes using the Genechip microarray system, primers will be developed to use for qRT-PCR. Initially analysis of de-identified samples will be tested in batches that correspond with the cohort type (surgical control, SIRS and sepsis). Assuming statistically significant differences between gene expression profiles from each group that are consistent with expected biological patterns in severe infection, systemic inflammatory or surgically-induced trauma, the samples will be randomly mixed into three batches that have no relationship to cohort. A statistician will develop a randomization sequence and an individual with no professional relationship or commercial interests in this project will redistribute the samples according the assigned sequence. Determination of SIRS, sepsis and surgical control patients will then be determined based on established gene expression differences. Additionally, this further qRT-PCR testing will also provide pertinent information on assay reproducibility.

A blood specimen will be collected to assess cell surface proteins tagged using immunofluorescent labels and run through a fluorescence-activated cell sorter (FACS), also referred to as flow cytometry, to ascertain protein expression profiles from white blood cells. These findings will be compared with gene expression profiles established from qRT-PCR to assess concordance.

A further blood specimen will be used for follow-up assessment of cytokines and immuno-inflammatory biomarkers used in current clinical practice for clarification on the immuno-inflammatory response.

As the definitive diagnosis of SIRS or septicaemia may not be known at the time patients are enrolled into this clinical study, a retrospective confirmation of diagnosis and assignment to the SIRS or Sepsis cohorts will be made. ***This process will be completed prior to any study-specific molecular analyses (with the exception of quality assurance processes that will be ongoing).***

ENDPOINTS

Primary:

1. Accurate diagnosis of septicaemia using a novel qRT-PCR molecular assay
2. Assay robustness (sensitivity and specificity) where gene expression profiles for septicaemia and SIRS can be accurately distinguished

Secondary:

1. Concordance between a gene expression profile of sepsis and the corresponding cell surface protein markers using flow cytometric methods
2. Vital signs (BP, HR, RR, O_2 saturation, temperature)
3. Clinical microbiology testing (e.g. blood culture, wound swab or sputum culture)

Tertiary:

1. Concomitant medications, blood and fluid products
2. Clinical laboratory testing (haematology and biochemistry)
3. Adverse events

STATISTICAL ANALYSES

A Statistical Analysis Plan (SAP) will be prepared by the study statistician prior to data un-blinding. This SAP will provide full details of preposed analysis including presentation of figures, table formats and statistical models.

Genechip® data will be processed to generate gene (rather than probe) level data using the RMA algorithm. A panel of genes chosen *a priori* from a previous proof of concept study of sepsis will be applied to generate a diagnostic profile (based on regularised discriminate analysis). The performance of this profile will be evaluated using 'leave one out' cross-validation. Permutation distributions of the cross-validated profile success outcomes will be generated. Study samples for Genechip® data will not be analysed from patients who do not meet the diagnosis of SIRS or sepsis or are not members of the control surgical cohort.

Diagnostic test characteristics will be measured including: sensitivity and specificity; positive and negative predictive values; positive and negative likelihood ratios. ROC analysis will be used to determine the optimal cut-off values for most efficient test

performance. All such evaluations will be based on cross-validated diagnostic significance and each performance measure will be tested against its permutation distribution (under random permutation of the group labels).

Part 2: RTT trial

Protocol Identification: Study Protocol # ATH-100-03-0609, "Continuous Monitoring of Patients with Severe Sepsis or Septic Shock using *SeptiCyte Lab* and Procalcitonin Comparator to Determine the Relationship Between Inflammatory Index and Clinical Progression and Outcome Measures".

Secondary Identifier: Clinical trial # ACTRN12610000465055 (Australian New Zealand Clinical Trials Registry).

Web link: https://www.anzctr.org.au/Trial/Registration/TrialReview.aspx?id=335567

Protocol Authors:
Dr Allison Sutherland BSc (Hons) PhD
Prof Deon Venter MB ChB PhD FRCPA MBA
Dr Mervyn Thomas BSc MSc Phil D
Dr Roslyn Brandon BVSc (Hons) PhD MBA MAICD
A/Prof Jeffrey Presneill MBBS (Hons) MBiostat PhD FRACP FCICM
Dr John Morgan MBBS FCICM
Prof Jeffrey Lipman MB ChB DA FFA FCICM MD
Prof Bala Venkatesh MBBS MD (Int. Med) FRCA FFARCSI MD (UK) FCICM

Version Dates:
Version 1: 29-Jun-2009
Version 2: 28-Jul-2009
Version 3: 29-Sep-2009
Version 4: 07-Jan-2010
Version 5: 19-Jul-2010
Version 6: 13-Aug-2010

Background: Early pilot studies led to the identification of a 120-gene signature for discriminating sepsis from other non-infectious inflammatory conditions. The GCP-1 trial was originally proposed as a means to validate this 120-gene signature. However, in the period between the GCP-1 and RTT trials, the signature was refined to include only 42 genes instead of the original 120 genes. The primary purpose of the RTT trial, as stated in the RTT study protocol, was to use the 42-gene signature to monitor patients with severe sepsis or septic shock, over a 10-day period and while in the ICU, to establish immune status.

Hypothesis (as stated in Study Protocol): It was hypothesized that the 42-gene molecular classifier could be use to distinguish sepsis from other non-infectious inflammatory conditions through comparing blood RNA profiles.[1]

---

[1] Note: the present work has progressed well beyond this early hypothesis, to encompass the search for additional biomarkers and biomarker combinations having enhanced diagnostic power.

Proposed Analytical Methods: microarray analysis; machine learning; comparison to procalcitonin (PCT).

Planned Cohort Sizes: 50 patients with severe sepsis or septic shock.

Inclusion Criteria: age > 18 years; body mass index < 40; signs and symptoms of severe sepsis or septic shock.

Exclusion Criteria: (1) Patients who have an autoimmune disease or other documented chronic immunological disorder e.g. SLE [systemic lupus erythematosus], Crohn's disease, rheumatoid arthritis, MS [multiple sclerosis], IDDM (type I diabetes) [insulin dependent diabetes mellitus]; (2) Oncology patients receiving chemotherapy within the last 3 months; (3) Solid-organ transplant recipients.

Study Sites: Mater Health Services (South Brisbane, Queensland); Royal Brisbane & Women's Hospital (Herston, Queensland); Princess Alexandra Hospital (Woollongabba, Queensland).

Analyses Actually Performed: For each patient enrolled in the RTT study, PAXgene Blood RNA samples were collected on multiple days as the patient passed through the ICU. Microarray data from the first collected sample were combined with microarray data from the previous GCP-1 study and reanalyzed, using more advanced bioinformatics methods. A new 4-gene signature with improved diagnostic performance was discovered.

Rationale for Deviation to Study Plan: (1) By combining data from the RTT study and the previous GCP-1 study, greater statistical power could be obtained because the number of patients in the Discovery Cohort was increased. (2) Bioinformatics methods of greater sophistication were used. This provided an enhanced ability to discover new biomarkers and biomarker combinations. The revised approach led to discovery of the 4-gene SeptiCyte® Lab signature, which proved to have greater diagnostic power with fewer genes compared to previously identified signatures. (3) Comparison to PCT was postponed until the validation phase so that *SeptiCyte Lab* and PCT could be directly compared using validation samples.

RTT Protocol Synopsis: attached (10 pages).

STUDY TITLE: Continuous Monitoring of Patients with Severe Sepsis or Septic Shock using SeptiCyte®Lab and Procalcitonin Comparator to Determine the Relationship Between Inflammatory Index and Clinical Progression and Outcome Measures

PROTOCOL NUMBER: ATH-100-03-0609

INVESTIGATIONAL PRODUCT: SeptiCyte®Lab

INDICATION: Diagnosis of, and monitoring of sepsis

PROTOCOL AUTHORS: Dr Allison Sutherland BSc (Hons) PhD
Prof Deon Venter MB ChB PhD FRCPA MBA
Dr Mervyn Thomas BSc MSc Phil D
Dr Roslyn Brandon BVSc (Hons) PhD MBA MAICD
A/Prof Jeffrey Presneill MBBS (Hons) MBiostat PhD FRACP FCICM
Dr John Morgan MBBS FCICM
Prof Jeffrey Lipman MB ChB DA FFA FCICM MD
Prof Bala Venkatesh MBBS MD (Int. Med) FRCA FFARCSI MD (UK) FCICM

SPONSOR:

Athlomics Pty Limited

PO Box 1448

Toowong Q 4066

Australia

CONFIDENTIAL

Property of Athlomics Pty Ltd

Information contained in this protocol should not be disclosed, other than to those directly involved in the execution or ethical review of the study, without written authorization from Athlomics Pty Ltd. It is however, permissible to provide information to a patient in order to obtain consent.

This study will be conducted in accordance to the guidelines in the National Statement on Ethical Conduct in Human Research ratified by the National Health & Medical Council (NHMRC) and Australian Vice Chancellors' Committee (AVCC) will be observed.

All information concerning the study assay developed by Athlomics Pty Ltd in connection with this clinical trial and not previously published is considered confidential and proprietary information. This information includes the clinical protocol, informed consent forms and case report forms (CRF).

Site Investigators are obliged to provide Athlomics Pty Ltd with complete study results and all data derived from this study. Only Athlomics Pty Ltd may enable information obtained during this study to be provided to the participating physicians or as required by law.

Protocol Approval

Authorised by:

Dr Roz Brandon
CEO, or Designee
Athlomics Pty Ltd

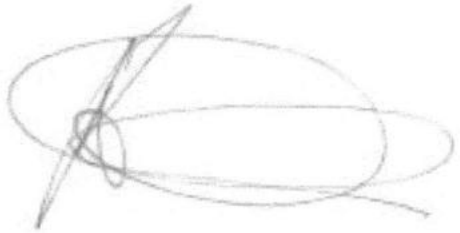

Date August 30, 2010

Amendment History

Version # 2 (dated 28 July 2009) includes the following amendment:

- Modification of the respiratory parameters and haematology results that are used to define presenting signs and symptoms of sepsis in the "Inclusion Criteria" and based on guidelines established by the American College of Chest Physicians/Society of Critical Care Medicine (1992). Specifically, on pages 7 and 18, arterial partial pressure ($PaCO_2$) should be less than 4.3 kilopascals (kPa) or less than 32 millimetres of mercury (mmHg). Additionally, reference to "leukocytopenia (≤1.0 x 10^9/L) or leukocytosis (>4.0 x 10^9/L)" on pages 7 and 18 has been amended to a white blood cell count of less than 4,000 cells/mm^3 (4 x 10^9 cells/L) or a white blood cell count greater than 12,000 cells/mm^3 (12 x 10^9 cells/L); or presence of greater than 10% immature neutrophils (bands forms).

Version #3 (dated 29 September 2009) includes the following amendment:

- Modification to the "Exclusion Criteria" on page 19, such that people of Aboriginal or Torres Strait Islander backgrounds may be included into this study even if they do have a higher probability of rheumatic fever.

Version #4 (dated 7 January 2010) includes the following amendments:

- RNA derived from PAXgene blood samples will additionally be used for analysis of bacterial load applying the same technology (quantitative real time PCR) as for the SeptiCyte®Lab testing.
- Change in the study plan to include only patients presenting with clinical signs and symptoms of severe sepsis or septic shock. These amendments are reflected by changes to the study title, study population, participant inclusion criteria and statistical methods. The rationale behind such amendments is to identify and monitor the immune response of a specific sepsis population that is at greatest risk of organ failure and death, as a result of systemic bacterial and/or fungal infection.
- Change in the study plan to include procalcitonin analysis as a comparator biomarker test

Version #5 (dated 19 July 2010) includes the following amendments:

- Change in the total number of patients to be recruited into this trial. As discussed in version #4 there was to be 30 patients with severe sepsis or septic shock recruited into this trial; however, based on changes to the secondary endpoint and statistical analysis plan, a total of 70 patients with severe sepsis and septic shock will be recruited into this trial
- Change to the objectives and endpoints to reflect that a formal superiority analysis will be conducted between SeptiCyte Lab and Procacitonin based on a composite secondary endpoint that includes Day 1 diagnosis and Day 2 to Day 5 clinical status.
- Change to the statistical analysis plan to include a comparison between SeptiCyte Lab and Procalcitonin using a modified per-protocol population
- Change to the statistical analysis plan to include details of two planned interim analyses

Version 6 (dated 13 August 2010) includes the following amendment:

- Changes to the exploratory objectives and endpoints to include analysis of circulating IL-6 (pro-inflammatory biomarker) and IL-10 (anti-inflammatory biomarker) levels.

1.0 PROTOCOL SYNOPSIS FOR ATH-100-03-0609

TITLE

Continuous Monitoring of Patients with Severe Sepsis or Septic Shock using SeptiCyte®Lab and Procalcitonin Comparator to Determine the Relationship Between Inflammatory Index and Clinical Progression and Outcome Measures

PROTOCOL NUMBER: ATH-100-03-0609

RATIONALE

Sepsis is a specific systemic inflammatory response to either a gram positive or gram negative bacterial or fungal infection. The cornerstone of sepsis diagnosis and prognosis for many decades has been identifying the causative circulating pathogen and quantitating single blood analytes to assess the patient's physiological response to the pathogen. However, it is an individual's immune system, which determines clinical escalation to septic shock and not the causative pathogen. Given that the immune response is complex and multifactorial there is a necessity for an assay that can expedite the early diagnosis of sepsis, as well as evaluate the individual's response to therapy/management. Recent developments in biomolecular technologies using quantitative reverse transcriptase polymerase chain reaction (qRT-PCR) enable gene expression patterns to be translated into diagnostic pathology profiles and have the capacity to improve acute clinical management.

"Athlomics" development has characterised a panel of 42 inflammatory gene expression biomarkers that significantly correlate with incidence of sepsis using both equine and human models. Athlomics preliminary outcomes, using data sets from local clinical trials, suggest that this investigational diagnostic has a better than 95% accuracy of detecting sepsis in patients admitted to a tertiary clinical setting with an acute non-specific immunoinflammatory response, based on area under the curve calculations using ROC[1] analyses. This is an important finding, and indicates that the specificity of the Athlomics sepsis signature is well within the performance band required for clinical use. To improve the strength of this signal Gene Expression Omnibus[1] (GEO) samples were compared with gene expression profiles from the sepsis cohort, demonstrating a specificity of greater than 99% based on ROC curves.[2] The Athlomics sepsis signature was applied to all currently available GEO Genechip data for human whole blood studies and a subset of 168 control samples, including both healthy controls and controls with known conditions not expected to produce inflammatory signals. These outcomes, suggest that this assay is robust and has the capacity to be used in future clinical practice for the definitive diagnosis of sepsis. ***Moreover, it has potential to be used in the practice of 'personalised' medicine, where an individual's gene expression signature can be used to structure a specific clinical management plan, determine response to therapy and provide prognostic updates.***

---

[1] Diagnostic performance is often measured by area under the ROC curve. An area of 1 implies perfect diagnostic performance; an area of 0.5 implies an entirely random (useless) diagnostic performance. Although the performance required is dependent on clinical context, ROC areas > 0.8 are considered `good' and ROC areas > 0.9 are considered excellent.

[2] The GEO database is an international publicly available archive of GeneChip data that can be used in research and product development for interrogation of proprietary gene signatures to demonstrate their preliminary validity for clinical application.

OBJECTIVES

Primary Objective: To evaluate the ability of SeptiCyte Lab (SCL) to monitor the immune status during severe sepsis based upon a gene expression biomarker signature, as well as observed change in clinical progression measures and therapeutic regimens (e.g. IV antibiotics, cardiac inotropes).

Secondary Objectives: A comparison between SCL and a comparator biomarker test, Procalcitonin (PCT), will be conducted to evaluate performance based on a composite secondary endpoint that includes initial diagnosis as well as clinical status between Day 2 to Day 5.

Tertiary Objectives: Assessment of correlations between the SCL signature and survival status at Day 28. Furthermore, circulating levels of IL-6 and IL-10 that are associated with pro-inflammatory and anti-inflammatory phases in the sepsis condition, respectively, will be assessed.

Exploratory Objectives: To investigate proteomic and metabolomic biomarkers which are associated with sepsis but not part of the SCL signature

STUDY PERIOD: 12 months

METHODOLOGY

This clinical trial is designed to evaluate whether a set of previously identified molecular (gene expression) biomarkers for sepsis can be optimized for the purposes of expediting diagnosis and improving clinical treatment plans. The study will continuously monitor up to 70 participants from the ICU, admitted due to severe sepsis or as a result of septic shock. Specifically, sepsis gene expression profiles will be continuously monitored in study participants for up to 10 days from the time of informed consent.

A definitive diagnosis of sepsis may not be known at the time of participation in this clinical study. It is anticipated there will be an approximate 30% attrition rate based on current survival statistics of patients with severe sepsis or in septic shock. Hence, in order to achieve a study cohort of 50 (per protocol) patients a total of 70 ICU patients with severe sepsis or in septic shock will be recruited into this study. Data collected from participants who are not diagnosed with sepsis will only be assessed for frequency of adverse events. Participants (or the substitute health authority) will be informed at the time of consent that their study research samples may not be analysed if sepsis is not confirmed from clinical microbiology testing. In such instances, all research blood and urine samples will be destroyed.

Following a review of the inclusion and exclusion criteria, participants or the substitute health authorities who sign the patient informed consent form (PICF) and meet all of entry criteria will be offered the opportunity to participate in this study. Patients who have clinical signs and/or symptoms of sepsis will be consented and enrolled into the study as soon as possible after admission, and ideally within 24 hours. The final assessment of sepsis will be made by a Principal Investigator as information becomes available.

On Day 1 following informed consent, demography and microbial status will be recorded in the case report form (CRF). Vital signs (blood pressure, heart rate, respiratory rate, oxygen saturation and tympanic temperature), APACHE II score, SOFA scores, concomitant medication, clinical laboratory testing (haematology and biochemistry) and adverse events will be recorded during the intervention and follow up visits for this study, between Days 1 to Day 11. Two PaxGene blood specimens will be collected once daily on Days 1, 2, 3, 5, 7,

and 10 for the purposes of performing SeptiCyte Lab and bacterial load[3] assays. Furthermore (paediatric) EDTA and SST blood specimens, as well as urine specimens (10ml) will be collected on Days 1, 2, 3, 5, 7 and 10 for procalcitonin (PCT) and proteomic/ metabolomic testing, respectively. It should be noted that the total volume of blood to be collected for this research study will be no more than 45ml (rounded up to the nearest 5ml). On Day 28, survival status will be noted in the CRF. A schematic of the investigational plan is presented in Appendix A.

All molecular laboratory studies will be conducted at Mater Pathology in accordance with NATA accredited standard operating procedures (SOP). In brief, RNA extracted (SOP_RNA Isolation) from blood specimens will be reverse-transcribed to create double-stranded complementary DNA (cDNA) and then amplified with specific primers using quantitative multiplex PCR (SOP_cDNA synthesis and qRT PCR). Primers will be developed to enable gene expression of specific molecular gene expression biomarkers identified from pre-clinical and early phase clinical studies that correlate with sepsis. Furthermore, cRNA will be synthesised for GeneChip®Human Exon (microarray) hybridization (SOP_cRNA and Genechip® Hybridization) to better delineate differences in gene expression from patients with sepsis.

NUMBERS OF SUBJECTS

This study will recruit 70 patients from the intensive care unit (ICU), who have clinical signs and symptoms consistent with severe sepsis or septic shock.

It is expected that there will be an approximate 30% attrition rate based on current survival statistics of patients with severe sepsis or in septic shock. Hence, in order to achieve a study cohort of 50 (per protocol) patients a total of 70 ICU patients with serve sepsis or in septic shock will be recruited, to for molecular analysis of immune function.

MAIN CRITERIA FOR INCLUSION

1. Aged over 18 years
2. Body Mass Index < 40
3. Clinical signs and symptoms of severe sepsis or septic shock. Criteria for severe sepsis is based a modified version to the original definitions presented by Bone et al.[2-4] In brief, patients must be admitted to the ICU with two or more signs of systemic inflammation within the last 24 hours, with a proven or suspected source of infection and the sepsis-induced dysfunction of at least one organ or system. The absence of positive culture results will not affect clinical diagnosis of sepsis.
    - Systemic Inflammatory Response (SIRS) Criteria: temperature >38°C or <36°C; heart rate >90 beats/min; respiratory rate >20 breathes/min or a $PaCO_2$ of <4.3 kPa (<32 mm Hg) or mechanical ventilation; and a white blood cell count <4,000 cells/mm^3 (<4 x 10^9 cells/L) or >12,000 cells/mm^3 (>12 x 10^9 cells/L) or >10% immature neutrophils (band forms). (Consensus from the American College of Chest Physicians/ Society of Critical Care Medicine)[3-4]
    - Infection Criteria: evidence of proven or suspected infection as demonstrated by one or more of the following: white cells in a normally sterile body fluid;

[3] The purpose of conducting this additional test is to characterise systemic bacterial load using gene expression analysis which will enable further differentiation between (local) bacterial colonization versus systemic infection

perforated viscus; radiographic evidence of pneumonia in association with the production of purulent sputum; a syndrome related to a high risk of infection[4]

- Organ/System Dysfunction Criteria:

  Cardiovascular – systolic arterial blood pressure of ≤90 mmHg or the mean arterial pressure of ≤70 mmHg for >1 hr, despite adequate fluid resuscitation, adequate intravascular volume status or the use of vasopressors in an attempt to maintain a systolic pressure of ≥90 mmHg or a mean arterial pressure of ≥70 mmHg;

  Pulmonary – PaO_2/FiO_2 ratio ≤250 in the presence of other dysfunctional organs or systems or ≤200 if the lung is the only dysfunctional organ;

  Renal – urine output of <0.5 ml/kg of body weight/ hr for 1 hour, despite adequate fluid resuscitation;

  Unexplained Metabolic Acidosis – pH ≤7.30 or a base deficit of ≥5.0 mmol/L in association with a plasma lactate level that is >1.5 times the upper limit of the local laboratory reference range

  Haematologic – platelet count <80,000/mm^3 (<80,000 μL), or platelet count has decreased by 50% in the 3 days preceding enrolment.[4-5]

4. The patient and substitute health authority are fluent in English (both written and spoken) and the patient or substitute health authority are capable of providing informed consent to participate in the study

---

EXPERIMENTAL ASSAYS:

Human exon genechips will be used as a high throughput analysis system to focus the selection of key gene expression markers in sepsis for application in clinical qRT PCR testing. These Genechip investigations will continue in this Clinical study for the purposes of optimizing the molecular gene expression profile for sepsis. Additionally, the utility of this investigational diagnostic in guiding therapeutic treatment regimens and providing prognostic information via continuous monitoring of a 10-day period will also be examined.

Furthermore, blood and urine specimens will be used for assessment of cytokines and immuno-inflammatory protein biomarkers for further investigation of the immuno-inflammatory response to sepsis.

ENDPOINTS

Primary:

Development of a machine-learning algorithm to predict sepsis status using sepsis gene expression biomarker signatures, clinical progression measures (APACHE II and SOFA), between Day 1 to Day 10.

Secondary:

A comparison of SCL and PCT, based on a composite endpoint including:

- Day 1 sepsis status

- Change in clinical progression and outcome measures (APACHE II and SOFA) between Day 2 and Day 5
- Change in therapeutic regimen between Day 2 and Day 5

Tertiary:

1. To investigate the relationship between the SeptiCyte inflammatory index and survival status at Day 28. The sample size is too small to allow reasonable power for a censored survival analysis. Instead, exploratory analyses will be performed seeking to identify differences in the inflammatory index between those patients who survive (anticipated that two thirds of patients will survive), and those who die (it is anticipated that one third of patients will die). The study will have 80% power to detect a difference between survivors and others of 1.8 standard deviations. Whilst power is too low to provide a definitive answer to research hypotheses about survival, the analysis will nonetheless provide useful information for the design of future studies.
2. To identify broader gene expression changes (not summarised through the SeptiCyte inflammatory index) associated with survival. Analyses will make use of empirical Bayse moderated t tests[4] and Bayesian gene selection algorithms.[5]
3. Assessment of circulating IL-6 and IL-10 that are associated with the pro-inflammatory and anti-inflammatory phases, respectively, of the sepsis condition.

Exploratory:

Assessment of proteomic and metabolomic biomarkers not part of SCL that are associated with the sepsis condition

STATISTICAL ANALYSES

This is a preliminary study, where there is no published work to provide guidance on correlation outcomes between an inflammatory index and clinical outcomes measures (as defined in protocol ATH-100-03-0609). Thus, it is not possible to set a sample size using formal calculations and instead, they have been estimated using industry norms of other similar exploratory studies. Historically, 20 patients have been sufficient to develop a robust inflammatory index.

A Statistical Analysis Plan (SAP) will be prepared by the study statistician prior to data un-blinding. This SAP will provide full details of preposed analysis including presentation of figures, table formats and statistical models.

Genechip®Human Exon (microarray) data will be processed to generate gene and probe level data using the RMA algorithm. A panel of 42 genes chosen *a priori* from a previous proof of concept study of sepsis and validated in a pilot study will be applied to determine correlations between the SeptiCyte Lab inflammatory index and defined clinical progression and outcome measures using a semiparametric regression model. The SeptiCyte Lab inflammatory index will be related to patient survival using logistic regression. The ROC area for this index will be compared with the ROC area for PCT using a bootstrap technique.

---

[4] Gordon K Smyth (2005). Limma: linear models form microarray data in Bioinformatics and Computational Biology Solutions using R and Bioconductor.(R. Gentleman, A.V Carey, S Dudoit, R. Irizarry and W. Huber [Eds.]) pg: 397-420, Springer, New York

[5] Harri T Kiiveri (2008). A general approach to simultaneous model fitting and variable elimination in response models for biological data with many more variables than observations. BMC Bioinformatics. 9:195.

Part 3: MARS study

Protocol Identification: “Molecular Diagnosis and Risk Stratification of Sepsis (MARS)”.

Secondary Identifier: NCT01905033 (ClinicalTrials.gov)

Web link: https://clinicaltrials.gov/ct2/show/NCT01905033

Principal Investigator: T. van der Poll, Academisch Medisch Centrum Universiteit van Amsterdam (AMCUvA).

Sponsor: Academisch Medisch Centrum Universiteit van Amsterdam (AMCUvA)

Collaborators: Center for Translational Molecular Medicine, UMC Utrecht, Radboud UMC Nijmegen, Philips Healthcare, Microbiome, Immunetrics, Biocartis, Immunexpress.

Background: Sepsis is a major cause of in-hospital morbidity and mortality. Current tools available to the clinician to initiate therapy of patients with sepsis mainly comprise of symptom classification systems and culture techniques, which provide aspecific and slow information.

Objective: The ultimate goal of the MARS program is to provide tools to assist the physician at the bedside in tailoring the treatment of an individual patient suffering from sepsis by generating rapid molecular information about the causative pathogen and the host response.

Deliverables: The MARS program seeks to deliver tests ("sample in / result out") that can be used by health care personnel at or close to the bedside and that provide rapid information (within two hours) about the presence or absence of sepsis, the causative pathogen and the risk of the individual patient for sepsis complications and death.

Design: The MARS program is organized into four Work Packages (WPs) along a clinical, discovery and technology platform. In WP3 (Host Response), RNA from blood cells will be analyzed to find novel biomarkers and to develop rapid and easy to perform tests that provide information about the risk profile of the patient. In addition, plasma levels of selected protein biomarkers will be measured for comparison of their value with that of the identified leukocyte molecular signatures.

Hypotheses: (1) It was hypothesized that the 4-gene *SeptiCyte Lab* signature, discovered through analysis of microarray data from the GCP-1 and RTT trials, could be independently validated in multiple cohorts from the MARS study using RT-qPCR technology. (2) It was hypothesized that the performance of *SeptiCyte Lab* would compare favorably with PCT and CRP for discriminating sepsis from infection-negative systemic inflammation.

Proposed Analytical Methods: RT-qPCR to validate the previous microarray results; comparison to procalcitonin (PCT); comparison to C-reactive protein (CRP); comparison to logistic combinations of clinical parameters monitored during ICU stay.

Planned Cohort Sizes: Multiple cohorts totaling approximately 350 patients.

Inclusion Criteria: As described in the Consort Diagram of Figure 1 (in manuscript).

Exclusion Criteria: As described in the Consort Diagram of Figure 1 (in manuscript).

Study Sites: Academic Medical Center (AMC) of Amsterdam; University Medical Centre of Utrecht (UMCU).

Analyses Performed: RT-qPCR measurements on all patients in Validation Cohorts 1-5; PCT measurement on 74 sepsis patients and 137 controls from Validation Cohorts 1+3+5; CRP measurement on 22 sepsis patients and 112 controls from Validation Cohorts 1+3+5; logistic regression analysis of 19 parameters from Table 1 in Levy et al. (2003) for which univariate $p < 0.05$ for separation of sepsis and controls in Validation Cohorts 1+3+5. These analyses have been summarized in manuscript Figures 3,4,6; Tables 3,4; and Supporting Information S11.

MARS Protocol Synopsis: attached (3 pages).

Molecular Diagnosis and Risk Stratification of Sepsis (MARS)

This study is currently recruiting participants. (see Contacts and Locations)

Verified August 2013 by Academisch Medisch Centrum - Universiteit van Amsterdam (AMC-UvA)

Sponsor:
Academisch Medisch Centrum - Universiteit van Amsterdam (AMC-UvA)

Collaborators:
Center for Translational Molecular Medicine
UMC Utrecht
Radboud University
Philips Healthcare
Microbiome
Immunetrics
Check-Points
Biocartis
ImmuneXpress

Information provided by (Responsible Party):
T. van der Poll, Academisch Medisch Centrum - Universiteit van Amsterdam (AMC-UvA)

ClinicalTrials.gov Identifier:
NCT01905033

First received: July 11, 2013
Last updated: August 21, 2013
Last verified: August 2013
History of Changes

Purpose

Background: Sepsis is a major cause of in-hospital morbidity and mortality. Current tools available to the clinician to initiate therapy of patients with sepsis mainly comprise of symptom classification systems and culture techniques, which provide aspecific and slow information.

Objective: The ultimate goal of this program is to assist the physician at the bedside in tailoring the treatment of an individual patient suffering from sepsis by generating rapid molecular information about the causative pathogen and the host response.

Deliverables: Rapid tests ("sample-in-result-out") that can be used by health care personnel at or close to the bedside and that provide rapid information (within two hours) about the presence or absence of sepsis, the causative pathogen and the risk of the individual patient for sepsis complications and death.

Design: The program is organized into four Work Packages (WPs) along a clinical, discovery and technology platform. In WP1 two university hospitals will enroll 7500 patients admitted to the Intensive Care Unit during the first 3 years of the project; 25% - 40% of these patients will have or will develop sepsis. In WP2 (Pathogen Detection), blood obtained from these patients will be used to develop rapid, fully automated DNA-based bedside tests that identify microorganisms and also provide information about their resistance to antibiotics. In WP3 (Host Response), RNA from blood cells will be analyzed to find novel biomarkers and to develop rapid and easy to perform tests that provide information about the risk profile of the patient. In addition, plasma levels of selected protein biomarkers will be measured for comparison of their value with that of the identified leukocyte molecular signatures. WP4 is responsible for the ICT management of the project. The Clinical Platform (covered by WP1 and WP4) delivers patient data and biological samples to the discovery and technology platforms. The Discovery Platform (covered by WP2 and WP3) uses patient data and biological samples to develop tests for detection of the infectious agent causing sepsis and for stratification of patients according to their risk for sepsis complications, including death. The results generated within the discovery platform will be delivered to the technology platform. The Technology Platform (part of WP2 and WP3) has the specific aim to develop rapid assays that run on a fully automated (micro)fluidics platform that is so easy to operate that it can be used in decentralized settings such as (close to) the ICU. The developed assays will make use of the knowledge generated in the discovery platform.

Condition
Sepsis

Study Type: Observational [Patient Registry]
Study Design: Observational Model: Cohort

	Time Perspective: Prospective
Target Follow-Up Duration:	1 Year
Official Title:	Molecular Diagnosis and Risk Stratification of Sepsis

Resource links provided by NLM:

MedlinePlus related topics: Sepsis

U.S. FDA Resources

Further study details as provided by Academisch Medisch Centrum - Universiteit van Amsterdam (AMC-UvA):

Primary Outcome Measures:

- Molecular information about causative pathogens and the host response in patients with sepsis [Time Frame: One year] [Designated as safety issue: No]

Secondary Outcome Measures:

- Stratification of septic patients by severity and type of immune response to infection [Time Frame: Five years] [Designated as safety issue: No]

Biospecimen Retention: Samples With DNA

Whole blood, plasma, RNA, DNA.

Estimated Enrollment:	7500
Study Start Date:	January 2011
Estimated Study Completion Date:	June 2014
Estimated Primary Completion Date:	June 2014 (Final data collection date for primary outcome measure)

Eligibility

Ages Eligible for Study:	18 Years and older
Genders Eligible for Study:	Both
Accepts Healthy Volunteers:	No
Sampling Method:	Probability Sample

Study Population

In 3-4 years all patients> 18 years in the Intensive Care Units of the AMC Amsterdam and UMC Utrecht will be included in the study with the exemption of elective cardiac surgery patients with an uncomplicated stay.

Criteria

Inclusion Criteria:

- All patients> 18 years in the Intensive Care Units of the AMC Amsterdam and UMC Utrecht.

Exclusion Criteria:

- Elective cardiac surgery patients with an uncomplicated stay.

Contacts and Locations

Choosing to participate in a study is an important personal decision. Talk with your doctor and family members or friends about deciding to join a study. To learn more about this study, you or your doctor may contact the study research staff using the Contacts provided below. For general information, see Learn About Clinical Studies.

Please refer to this study by its ClinicalTrials.gov identifier: NCT01905033

Contacts

Contact: Tom van der Poll, Prof. +31205665910 t.vanderpoll@amc.uva.nl

Locations

Netherlands

Academic Medical Center **Recruiting**

Amsterdam, Noord-Holland, Netherlands, 1105 AZ
Contact: Tom van der Poll, Prof.
Principal Investigator: Tom van der Poll, Prof.

University Medical Center Utrecht **Recruiting**
Utrecht, Netherlands, 3584 CX
Contact: Marc J Bonten, Prof. mbonten@umcutrecht.nl
Principal Investigator: Marc J. Bonten, Prof.

Sponsors and Collaborators

Academisch Medisch Centrum - Universiteit van Amsterdam (AMC-UvA)

Center for Translational Molecular Medicine

UMC Utrecht

Radboud University

Philips Healthcare

Microbiome

Immunetrics

Check-Points

Biocartis

ImmuneXpress

Investigators

Principal Investigator: Tom van der Poll, Prof. Academisch Medisch Centrum - Universiteit van Amsterdam (AMC-UvA)

More Information

No publications provided by Academisch Medisch Centrum - Universiteit van Amsterdam (AMC-UvA)

Additional publications automatically indexed to this study by ClinicalTrials.gov Identifier (NCT Number):

Klein Klouwenberg PM, Zaal IJ, Spitoni C, Ong DS, van der Kooi AW, Bonten MJ, Slooter AJ, Cremer OL. The attributable mortality of delirium in critically ill patients: prospective cohort study. BMJ. 2014 Nov 24;349:g6652. doi: 10.1136/bmj.g6652.

Responsible Party:	T. van der Poll, Prof. dr. T. van der Poll, Academisch Medisch Centrum - Universiteit van Amsterdam (AMC-UvA)
ClinicalTrials.gov Identifier:	NCT01905033 History of Changes
Other Study ID Numbers:	10-056
Study First Received:	July 11, 2013
Last Updated:	August 21, 2013
Health Authority:	Netherlands: Medical Ethics Review Committee (METC)

Keywords provided by Academisch Medisch Centrum - Universiteit van Amsterdam (AMC-UvA):
Sepsis
Pneumonia
Peritonitis
Biomarkers
Transcriptomics
Pathogen detection

Additional relevant MeSH terms:
Sepsis
Toxemia
Infection
Inflammation
Pathologic Processes
Systemic Inflammatory Response Syndrome

ClinicalTrials.gov processed this record on May 25, 2015
