# Supplementary material for: A Molecular Host Response Assay to Discriminate Between Sepsis and Infection-Negative Systemic Inflammation in Critically Ill Patients: Discovery and Validation in Independent Cohorts
Source: PLoS Med. 2015 Dec 8;12(12):e1001916. doi: 10.1371/journal.pmed.1001916 (PMC4672921; doi:10.1371/journal.pmed.1001916)
Supplement: S2 Text — (PDF) [file pmed.1001916.s009.pdf]

S2 Text for McHugh et al., "A Molecular Host Response Assay to Discriminate Between Sepsis and Infection-Negative Systemic Inflammation in Critically Ill Patients: Discovery and Validation in Independent Cohorts"

Definition and Selection of Validation Cohorts

1. Definitions

ICU Admission Time: if a time-stamp is available, then the ICU Admission Time will equal the time-stamp. If no time-stamp is available, then the ICU Admission Time will be assumed to equal 12:00 (noon) on the indicated admission date (with an uncertainty of ±12 hours).

Infection Likelihood: the most plausible infection likelihood classification resulting from primary or secondary infection site(s), across all Relevant Sepsis Events.

Relevant Sepsis Event: any Sepsis Event occurring no more than 3 days prior to ICU admission, or no more than 2 days following ICU admission.

Sepsis Event: defined operationally as having occurred if a patient displayed 2 or more criteria for systemic inflammation according to [1-7] and was given systemic therapeutic antibiotics by the attending physician. In other words, a Sepsis Event was deemed to have occurred when the attending physician had a strong enough suspicion of infection that systemic therapeutic antibiotics were administered to the patient. Sepsis Events occurring after ICU admission were observed and dated directly by the attending physician. Sepsis Events occurring before ICU admission were identified retrospectively by the attending physician, through a process of inference based on the patient or caregiver's description of prior symptoms. Each Sepsis Event was retrospectively adjudicated to have an infection likelihood of none, possible, probable or definite (culture proven).

2. Selection of Patients from MARS Study & Exclusion Criteria

The MARS study employed the following general inclusion & exclusion criteria:

Inclusion: All patients >18 years of age admitted to the Amsterdam and Utrecht ICUs.

Exclusion: Elective cardiac surgery patients with an uncomplicated stay.

Of the 7,398 patients enrolled in the MARS study, 2,109 patients who enrolled from November 30, 2012 onwards were available to Immunexpress for the present study. Of this subset, 1,151 patients satisfied the following study-specific inclusion and exclusion criteria:

Inclusion:

1. Age between 18 and 89 years;
2. Exhibited at least two criteria of systemic inflammation.

Exclusion:

1. Patient excluded if the sepsis event nearest to ICU admission was earlier than 72 hours prior to ICU admission, or later than 48 hours after ICU admission;[1]

2. Patient excluded if blood samples were drawn more than 24 hours after ICU admission;

3. Patient excluded if a systemic therapeutic antibiotic regimen, other than prophylactic antibiotics routinely given after surgery, was administered more than 24 hours prior to admission in the ICU;

4. Microbiology test results excluded for specimens collected outside of a 72-hour window on either side of ICU admission.[2]

Note: hospital readmissions were allowed, subject to all the exclusion criteria listed above.

A flow diagram for selection of patients for this study has been presented as Figure 1 in the main text. A mapping of the patients from the Amsterdam and Utrecht ICUs to the five Validation Cohorts is presented in **Figure 1** below.

[1] Nearest sepsis event less than 48 hours after ICU admission is taken as evidence for community acquired infection. Nearest sepsis event greater than 48 hours after ICU admission is taken as evidence for hospital acquired infection.

[2] A patient who satisfied the study criteria might be included in the study, but some of his or her microbiology test results might be excluded from consideration based on specimen collection time.

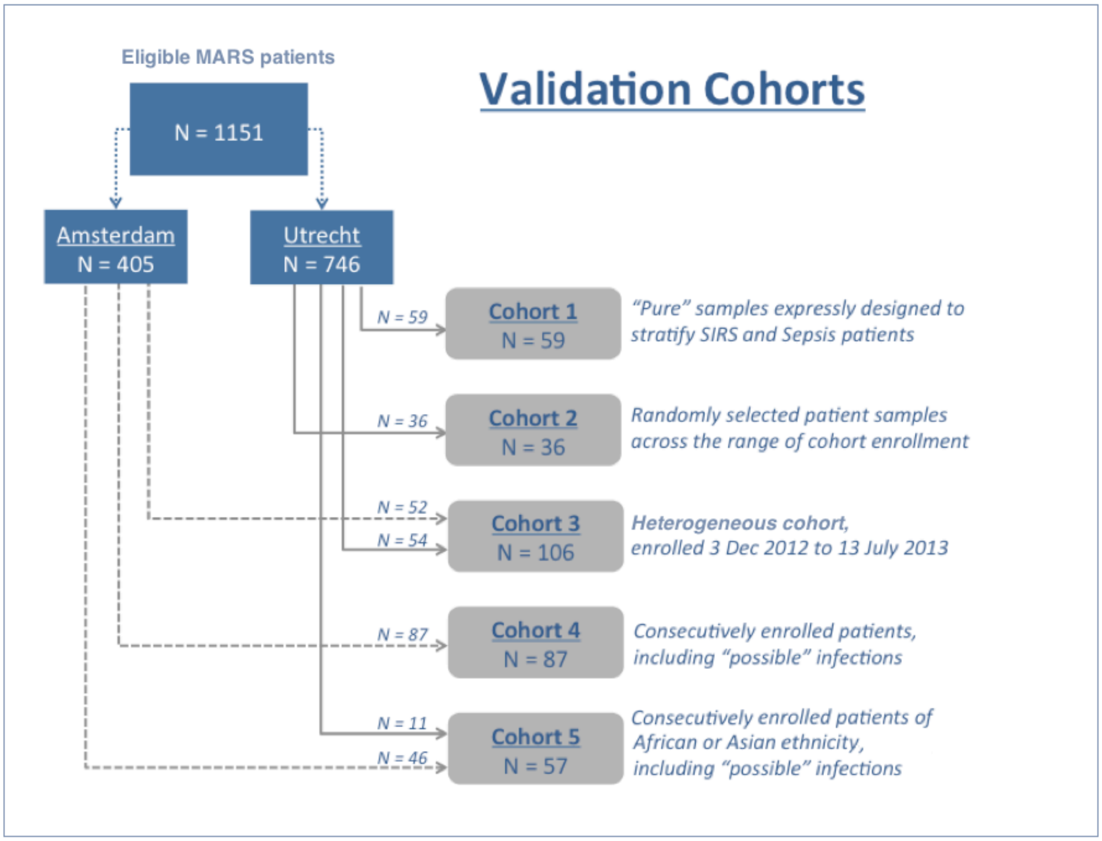

Figure 1: *Mapping of Validation Cohorts to the MARS Study Sites*

3. Procedure for Classifying Patients

A total of 345 patients satisfying the above criteria were selected to define the five validation cohorts of the present study. Patients in each validation cohort were characterized and classified by the following three-step process:

Step 1: Patients in each cohort were subjected to daily assessments by the attending physician(s), to estimate: 1) the severity of the Sepsis Event (if such an event occurred) and 2) the infection likelihood. In addition, physiological data were recorded daily. Patient information was analyzed each day to produce an infection likelihood assessment (definite, probable, possible or none) [5,6] and the previous day's assessment was overwritten.

Step 2: Retrospective analysis of all patient information, at a median time of 3 months post-admission, led to a final determination of infection likelihood and an identification of primary and any secondary sites of infection. Infection likelihood was assessed after taking into account all available information in the patient's medical chart. Infection likelihood was scored as definite (culture proven), probable, possible or none.

To perform the evaluation, two trial physicians on site (at either the Amsterdam or Utrecht ICU) examined each patient's chart. (The two physicians were randomly selected from a pool of 8 physicians tasked with assessing patients.) A third trial physician was used to adjudicate in cases of discordance over the infection likelihood as per [5].

Step 3: Patients were classified as follows:

- *Controls (Infection-Negative Systemic Inflammation):* This group consisted of: 1) patients who exhibited ≥2 clinical signs of systemic inflammation, but who were never assessed for infection likelihood, because they did not have a Sepsis Event, were not suspected of sepsis, and were never treated for sepsis; 2) patients who exhibited ≥2 clinical signs of systemic inflammation and experienced a Sepsis Event, and therefore as a precaution were given therapeutic systemic antibiotics, but who were then retrospectively adjudicated to have an infection likelihood of none.

- *Cases (Sepsis):* This group consisted of: 1) patients who experienced a Sepsis Event and were then retrospectively adjudicated to have an infection likelihood of probable; 2) patients who experienced a Sepsis Event and were then retrospectively adjudicated to have an infection likelihood of definite (culture proven).

- *Infection Likelihood of Possible:* Patients who experienced a Sepsis Event and were given therapeutic systemic antibiotics as a precaution, but who were then retrospectively adjudicated to have an infection likelihood of possible could not be classified with certainty as either cases or controls. These patients were excluded from performance analyses.

Note that, in proceeding from Step 2 to Step 3, there has been a translation from an Infection Likelihood classification (ordinal scale with the four values of definite, probable, possible, none) to a case/control classification (ordinal scale with the three values of case, control, and infection likelihood possible). Difficulties inherent in the process of translating between reference scales, when each reference scale is imperfect, have been discussed in [5, 8-10].

4. Definition of Validation Cohorts

Table 1 defines the five validation cohorts of this study.

Table 1: *Definition of the validation cohorts of this study*

Cohort	Infection Likelihood Assessment	Category
1 (n=59)	None/No Sepsis Event = 35 (59.3%)	Controls = 35 (59.3%)
	Possible = 0 (0%)	Cases = 24 (40.7%)
	Probable = 6 (10.2%)	
	Definite = 18 (30.5%)	
2 (n=36)	None/No Sepsis Event = 27 (75.0%)	Controls = 27 (90.0%)
	Possible = 6 (16.7%)	Cases = 3 (10.0%)
	Probable = 3 (8.3%)	
	Definite = 0 (0%)	
3 (n=106)	None/No Sepsis Event = 77 (72.6%)	Controls = 77 (72.6%)
	Possible = 0 (0%)	Cases = 29 (27.4%)
	Probable = 10 (9.4%)	
	Definite = 19 (17.9%)	
4 (n=87)	None/No Sepsis Event = 47 (54.0%)	Controls = 47 (70.1%)
	Possible = 20 (23.0%)	Cases = 20 (29.9%)
	Probable = 7 (8.0%)	
	Definite = 13 (14.9%)	
5 (n=57)	None/No Sepsis Event = 25 (43.9%)	Controls = 25 (54.3%)
	Possible = 11 (19.3%)	Cases = 21 (45.7%)
	Probable = 10 (17.5%)	
	Definite = 11 (19.3%)	
Total (=345)	None/No Sepsis Event = 211 (61.2%)	Controls = 211 (68.5%)
	Possible = 37 (10.7%)	Cases = 97 (31.5%)
	Probable = 36 (10.4%)	
	Definite = 61 (17.7%)	

Validation Cohort 1 (n=59) consisted only of patients for which the diagnosis as case or control was definite or probable. By design, this cohort did not include any patients with an infection likelihood of possible, whereas in the entire MARS database ~15% of patients are classified with an infection likelihood of possible. The patients in this cohort were admitted to the Utrecht ICU from December 2012 to March 2013, but were not enrolled sequentially. RT-qPCR data for this cohort were generated in July 2013.

Validation Cohort 2 (n=36) consisted of *36* patients exhibiting ≥ 2 criteria of

systemic inflammation, randomly picked from the Amsterdam ICU (n=19) or Utrecht ICU (n=17) over the entire available dates of the study (December 2012 to July 2013). This cohort included 6 patients with an infection likelihood of possible. This cohort was used to check that the *SeptiCyte Lab* signature was not skewed with respect to study enrolment date. RT-qPCR data for this cohort were generated in July and October 2013 (two batches).

Validation Cohort 3 (n=106) was drawn from an initial set of n=1004 patients consecutively admitted to the Amsterdam ICU (n=273) and Utrecht ICU (n=731) from December 2012 to July 2013. From this initial set, 150 patients with an infection likelihood of possible were deliberately excluded. An additional four patients were excluded because insufficient data were captured to meet the minimum reporting requirements for retrospective physician evaluation. From the remaining pool of 850 patients, random draws were made of 75 patients from the Amsterdam ICU and 74 patients from the Utrecht ICU. After then applying the study exclusion criteria, the final numbers of patients from the two study centers were 52 patients from the Amsterdam ICU, and 54 patients from the Utrecht ICU. RT-qPCR data for this cohort were generated in November 2013.

Validation Cohort 4 (n=87) consisted of patients exhibiting ≥ 2 criteria of systemic inflammation, drawn sequentially from the Amsterdam ICU from March 2013 to June 2013. Patients with an infection likelihood of possible were included. This cohort was used to assess performance in a real-world setting (sequential patients), and also the feasibility of porting the *SeptiCyte Lab* assay from one brand of RT-qPCR reagents to another brand of reagents. RT-qPCR data for this cohort were generated in August 2014.

Validation Cohort 5 (n=57) consisted of patients of Black or Asian ethnicity consecutively enrolled at the Amsterdam ICU (N=46) or Utrecht ICU (n=11) from November 2012 to August 2013. This cohort was used to test the performance of the *SeptiCyte Lab* classifier for bias due to ethnicity. This cohort included 11 patients with an infection likelihood of possible. RT-qPCR data for this cohort were generated in April 2014.

5. References

1. Bone RC, Balk RA, Cerra FB, Dellinger RP, Fein AM, Knaus WA, Schein RMH, Sibbald WJ: Definitions for sepsis and organ failure and guidelines for the use of innovative therapies in sepsis. The ACCP/SCCM Consensus Conference Committee. American College of Chest Physicians/Society of Critical Care Medicine. Chest 1992; 101:1644-1655

2. Bone RC, Sprung CL, Sibbald WJ: Definitions for sepsis and organ failure. Crit Care Med 1992; 20:724-726

3. Muckart DJ, Bhagwanjee S: American College of Chest Physicians/Society of Critical Care Medicine Consensus Conference definitions of the systemic inflammatory response syndrome and allied disorders in relation to critically injured patients. Crit Care Med 1997; 25: 1789-1795

4. Levy MM, Fink MP, Marshall JC, Abraham E, Angus D, Cook D, Cohen J, Opal SM, Vincent JL, Ramsay G: 2001 SCCM/ESICM/ACCP/ATS/SIS International Sepsis Definitions Conference. Crit Care Med 2003; 31(4):1250-1256

5. Klein Klouwenberg PMC, Ong DSY, Monten MJM, Cremer OL: Classification of sepsis, severe sepsis and septic shock: the impact of minor variations in data capture and definition of SIRS criteria. Intensive Care Med 2012; 38: 811-819

6. Klein Klouwenberg PM, Ong DS, Bos LD, de Beer FM, van Hooijdonk RT, Huson MA, Straat M, van Vught LA, Wieske L, Horn J, Schultz MJ, van der Poll T, Bonten MJ, Cremer OL: Interobserver agreement of centers for disease control and prevention criteria for classifying infections in critically ill patients. Crit Care Med 2013; 41: 2373-2378.

7. Dellinger RP, Levy MM, Rhodes A, Annane D, Gerlach H, Opal SM, Sevransky JE, Sprung CL, Douglas IS, Jaeschke R, Osborn TM, Nunnally ME, Townsend SR, Reinhart K, Kleinpell RM, Angus DC, Deutschman CS, Machado FR, Rubenfeld GD, Webb SA, Beale RJ, Vincent JL, Moreno R; Surviving Sepsis Campaign Guidelines Committee including the Pediatric Subgroup: Surviving sepsis campaign: international guidelines for management of severe sepsis and septic shock: 2012. Crit Care Med. 2013; 4: 580-637. PMID: 23353941

8. Rutjes AW, Reitsma JB, Coomarasamy A, Khan KS, Bossuyt PM: Evaluation of diagnostic tests when there is no gold standard. A review of methods.

Health Technol Assess 2007; 11(50): iii, ix-51. PMID: 18021577

9. Weiss M, Huber-Lang M, Taenzer M, Traeger K, Altherr J, Kron M, Hay B, Schneider M: Different patient case mix by applying the 2003 SCCM/ESICM/ACCP/ATS/SIS sepsis definitions instead of the 1992 ACCP/SCCM sepsis definitions in surgical patients: a retrospective observational study. BMC Med Inform Decis Mak. 2009; 9:25-33

10. Bertens LC, Broekhuizen BD, Naaktgeboren CA, Rutten FH, Hoes AW, van Mourik Y, Moons KG, Reitsma JB: Use of expert panels to define the reference standard in diagnostic research: a systematic review of published methods and reporting.
PLoS Med 2013; 10(10): e1001531. PMID: 24143138
