# Supplementary material for: A Molecular Host Response Assay to Discriminate Between Sepsis and Infection-Negative Systemic Inflammation in Critically Ill Patients: Discovery and Validation in Independent Cohorts
Source: PLoS Med. 2015 Dec 8;12(12):e1001916. doi: 10.1371/journal.pmed.1001916 (PMC4672921; doi:10.1371/journal.pmed.1001916)
Supplement: S3 Text — (PDF) [file pmed.1001916.s010.pdf]

S3 Text for McHugh et al., "A Molecular Host Response Assay to Discriminate Between Sepsis and Infection-Negative Systemic Inflammation in Critically Ill Patients: Discovery and Validation in Independent Cohorts"

STARD Checklist

1. Identify the article as a study of diagnostic accuracy (recommend MeSH heading 'sensitivity and specificity').

Title:
A Molecular Host Response Assay to Discriminate Between Sepsis and Infection-Negative Systemic Inflammation in Critically Ill Patients: Discovery and Validation in Independent Cohorts

Key Words:
systemic inflammation; sepsis; infection; classifier; microarray; RT-qPCR; diagnostic accuracy, likelihood ratios

2. State the research questions or study aims, such as estimating diagnostic accuracy or comparing accuracy between tests or across participant groups.

Abstract:
We hypothesized that a molecular classifier based on peripheral blood RNAs could be discovered that would: 1) determine which patients with systemic inflammation had sepsis; 2) be robust across independent patient cohorts; 3) be insensitive to disease severity; and 4) provide diagnostic utility. The goal of this study was to identify and validate such a molecular classifier.

3. Describe the study population: the inclusion and exclusion criteria, setting and locations where data were collected.

Discovery Cohort
Materials and Methods:
Cases (confirmed or probable sepsis) and controls (post-surgical patients with infection negative systemic inflammation) were recruited from the intensive care units (ICU) of five tertiary care settings (Wesley Hospital, Mater Adult Hospital, Mater Private Hospital, Princess Alexandra Hospital, and Royal Brisbane & Women's Hospital) within the Brisbane, Australia metropolitan area. The patients were recruited within two formal clinical studies designated GCP-1 (Australian Dept. Health & Ageing CTN Number 044/2008) and RTT (Australian New Zealand Clinical Trials Registry identifier ACTRN12610000465055).

Inclusion Criteria: All study participants were recruited from ICUs in the above hospitals, were 18 years or older, and provided written informed consent either as individuals or through surrogate decision-makers.

Exclusion Criteria: Patients were excluded if they had body mass index ≥40, displayed any systemic immunological disorders, were transplant recipients or currently receiving chemotherapy treatment for cancer or immunosuppressed for any other known reason, or had chronic localized bacterial or fungal infections.

Validation Cohorts

Materials and Methods:

Patients for the present study were selected from the Molecular Diagnosis and Risk Stratification of Sepsis (MARS) study (ClinicalTrials.gov Identifier NCT01905033), a prospective observational cohort study in the Netherlands designed to produce molecular information relevant to sepsis diagnosis and management. The MARS study recruited ICU patients across two tertiary teaching hospitals: the Academic Medical Center (AMC) of Amsterdam, and the University Medical Centre of Utrecht (UMCU). Patients in the MARS study consisted of adults admitted to ICU, with the exclusion of cardiac elective surgery patients with an uncomplicated short stay. Patients admitted to ICU / enrolled in the MARS study from December 2012 to July 2013 were made available for inclusion in the present study.

[Inclusion and Exclusion Criteria] For patients enrolled in the MARS study, a Sepsis Event was defined operationally to have occurred when a patient displayed two or more signs of systemic inflammation, and was given therapeutic systemic antibiotics by the attending physician within 24 hours of ICU admission. … Patients having Sepsis Events within the interval from three days before ICU admission to two days after ICU admission were considered for inclusion in the present study. Patients were excluded if the Sepsis Event nearest to ICU admission fell outside this interval.

Results, and S2 Text:

The process of including/excluding patients to create the Validation Cohorts is further described in the flow diagram of Figure 1, and in S2 Text (PDF file) entitled "Definition and Selection of Validation Cohorts".

4. Describe participant recruitment: was recruitment based on presenting symptoms, results from previous tests, or the fact that the participants had received the index tests or the reference standard?

Recruitment of all patients was based on presenting symptoms, as detailed below.

Discovery Cohort

Materials and Methods:

Cases (confirmed or probable sepsis) and controls (post-surgical patients with infection negative systemic inflammation) were recruited from the intensive care units (ICU) of five tertiary care settings (Wesley Hospital, Mater Adult Hospital, Mater Private Hospital, Princess Alexandra Hospital, and Royal Brisbane & Women's Hospital) within the Brisbane, Australia metropolitan area.

Inclusion Criteria: All study participants were recruited from ICUs in the above hospitals, were 18 years or older, and provided written informed consent either as individuals or through surrogate decision-makers.

Exclusion Criteria: Patients were excluded if they had body mass index ≥40, displayed any systemic immunological disorders, were transplant recipients or currently receiving chemotherapy treatment for cancer or immunosuppressed for any other known reason, or had chronic localized bacterial or fungal infections.

Patients were recruited sequentially at each study site, subject to the stated inclusion and exclusion criteria.

<u>Results:</u>

The Discovery Cohort (n=105) consisted of consecutively enrolled patients in two groups: a post-surgical control group with infection-negative systemic inflammation (n=31), and a sepsis group (n=74). The control group was composed entirely of patients who underwent elective invasive surgery for non-infection related conditions. The sepsis group was composed of patients diagnosed as septic upon ICU admission.

Heterogeneity of Cases (Septic Patients): Besides the specific exclusions listed in footnote #1 of Table 1, no patients were excluded based on predisposing condition, organ system affected, co-morbidities, presence of natural immunosuppression or immunosenescence, therapies, or type of pathogen detected. Patients with both systemic and local infections were included. A wide variety of pathogens were identified including Gram-positive bacteria, Gram-negative bacteria, fungi and mixed infections. Viral infections were generally not tested for.

<u>Validation Cohorts</u>

<u>Materials and Methods:</u>

Patients for the present study were selected from the Molecular Diagnosis and Risk Stratification of Sepsis (MARS) study (ClinicalTrials.gov Identifier NCT01905033), a prospective observational cohort study in the Netherlands designed to produce molecular information relevant to sepsis diagnosis and management. The MARS study recruited ICU patients across two tertiary teaching hospitals: the Academic Medical Center (AMC) of Amsterdam, and the University Medical Centre of Utrecht (UMCU). Patients in the MARS study consisted of adults admitted to ICU, with the exclusion of cardiac elective surgery patients with an uncomplicated short stay. Patients admitted to ICU / enrolled in the MARS study from December 2012 to July 2013 were made available for inclusion in the present study.

For patients enrolled in the MARS study, a Sepsis Event was defined *operationally* to have occurred when a patient displayed two or more signs of systemic inflammation, and was given therapeutic systemic antibiotics by the attending physician within 24 hours of ICU admission. In other words, a Sepsis Event was deemed to have occurred when the ICU clinician had sufficient suspicion of sepsis to prescribe therapeutic systemic antibiotics. In some cases, a Sepsis Event was adjudicated retrospectively to have occurred several days before ICU admission, but was unrecognized at the time of occurrence. Patients having Sepsis Events within the interval from three days before ICU admission to two days after ICU admission were considered for inclusion in the present study. Patients were excluded if the Sepsis Event nearest to ICU admission fell outside

this interval. For the Sepsis Event nearest to ICU admission, a physician-assessed infection likelihood estimate of none, possible, probable, or definite was assigned retrospectively according to Center for Disease Control and Prevention and International Sepsis Forum consensus definitions [4, 24, 26]. See S2 Text, "Definition and Selection of Validation Cohorts" for additional detail.

Patients in each of the five Validation Cohorts were classified as either sepsis cases or infection-negative systemic inflammation controls. Patients were classified as sepsis cases if they experienced a Sepsis Event and were then adjudicated to have infection likelihood probable or definite for that event. Patients were classified as controls if: (1) they displayed two or more symptoms of systemic inflammation but were never given therapeutic systemic antibiotics (i.e. did not have a Sepsis Event); or (2) they displayed two or more symptoms of systemic inflammation and were given therapeutic systemic antibiotics (i.e. operationally defined to have had a Sepsis Event) but were then retrospectively adjudicated to have an infection likelihood of none. Some patients could not be classified with certainty into either the sepsis or control category. These were patients who operationally had a Sepsis Event but who were retrospectively adjudicated to have an infection likelihood of possible, meaning it ultimately was unclear whether the patient had sepsis or not. These patients were excluded from performance analyses but included in an analysis of factors leading to classification uncertainty.

5. Describe participant sampling: was the study population a consecutive series of participants defined by the selection criteria in item 3 and 4? If not, specify how participants were further selected.

Discovery Cohort

Materials and Methods:

Patients were recruited sequentially at each study site, subject to the stated inclusion and exclusion criteria. Patients for the GCP-1 study were recruited from July 2008 to August 2009, and patients for the RTT study were recruited from May 2010 to December 2011.

Results:

The Discovery Cohort (n=105) consisted of consecutively enrolled patients classified as either cases (n=74) or controls (n=31).

Validation Cohorts

Results:

A total of 345 patients from the MARS study were selected for analysis and were grouped into five Validation Cohorts. Figure 1 presents a flow diagram indicating the inclusion and exclusion criteria used for patient selection. The Validation Cohorts comprising 59, 36, 106, 87 and 57 patients respectively (described below) were chosen for different purposes and accordingly had different clinical and demographic characteristics (Table 2, S2 Text, and below). A mapping of the patients in the Validation Cohorts back to the MARS study sites is given in S2 Text. The Validation Cohorts are briefly described as follows.

Validation Cohort 1 (n=59 consisting of 24 cases, 35 controls) contained only patients diagnosed with high confidence as having either sepsis or infection-negative systemic inflammation. This cohort consisted of patients admitted to the Utrecht ICU from December 2012 to March 2013, but not sequentially.

Validation Cohort 2 (n=36 consisting of 3 cases, 27 controls, 6 infection likelihood possible) contained patients that were randomly picked from the Amsterdam ICU (n=19) or Utrecht ICU (n=17) with ICU admission dates spanning the entire time frame of interest (December 2012 to July 2013). This cohort was used to test whether the score generated by SeptiCyte Lab exhibited any bias with respect to ICU admission date.

Validation Cohort 3 (n=106 consisting of 29 cases, 77 controls) was drawn from a consecutive sequence of 775 patients admitted to the Amsterdam ICU and Utrecht ICU from December 2012 to mid-July 2013. From this initial set of patients, 91 with an infection likelihood of possible (91/775 = 11.7%) were deliberately excluded. An additional four patients were excluded because insufficient data were captured to meet the minimum reporting requirements for retrospective physician adjudication. From the remaining pool (n=680), patients were randomly drawn to define this cohort (n=52 from Amsterdam and n=54 from Utrecht).

Validation Cohort 4 (n=87 consisting of 20 cases, 47 controls, 20 infection likelihood possible) consisted of patients who were consecutively admitted to the Amsterdam ICU from mid-March to mid-June, 2013. This cohort was used to assess performance in a real-world setting (i.e. sequential patients).

Validation Cohort 5 (n=57 consisting of 21 cases, 25 controls, 11 infection likelihood possible) contained exclusively patients of African or Asian ethnicity, who were consecutively admitted to the Amsterdam ICU (n=46) or Utrecht ICU (n=11) from December 2012 through July 2013. This cohort was used to determine if the performance of *SeptiCyte Lab* was affected by ethnicity in a real-world setting (i.e. sequential patients).

6. Describe data collection: was data collection planned before the index test and reference standard were performed (prospective study) or after (retrospective study)?

Discovery Cohort
Materials and Methods:

The study protocols were finalized and the requisite ethics approvals were obtained prior to the recruitment of patients in each study.

Validation Cohorts
Materials and Methods:

Patients for the present study were selected from the Molecular Diagnosis and Risk Stratification of Sepsis (MARS) study (ClinicalTrials.gov Identifier NCT01905033), a prospective observational cohort study in the Netherlands designed to produce molecular information relevant to sepsis diagnosis and management. … Patients admitted to ICU / enrolled in the MARS study from December 2012 to July 2013 were made available for inclusion in the present study.

All final classification of patients as either sepsis cases or controls was completed within three months of ICU admission, and locked before gene expression analysis of blood samples was initiated.

7. Describe the reference standard and its rationale.

Discovery Cohort

Materials and Methods:

Final diagnosis of sepsis or infection-negative systemic inflammation was made by retrospective physician assessment using all available clinical and microbiological data and according to the ACCP/SCCM Consensus Statement for sepsis [23]. Consensus evaluations were made by two ICU physicians for the GCP-1 study and four ICU physicians for the RTT study, and were locked before microarray analysis of blood samples was initiated.

Validation Cohorts

Materials and Methods:

For the Sepsis Event nearest to ICU admission, a physician-assessed infection likelihood estimate of none, possible, probable, or definite was assigned retrospectively according to Center for Disease Control and Prevention and International Sepsis Forum consensus definitions [4, 24, 26]. See S2 Text for additional detail.

Patients in each of the five Validation Cohorts were classified as either cases (sepsis) or controls (infection-negative systemic inflammation). Patients were classified as cases if they experienced a Sepsis Event and were then adjudicated to have infection likelihood probable or definite for that event. Patients were classified as controls if: (1) they displayed two or more symptoms of systemic inflammation but were never given therapeutic systemic antibiotics (i.e. did not have a Sepsis Event); or (2) they displayed two or more symptoms of systemic inflammation and were given therapeutic systemic antibiotics (i.e. operationally defined to have had a Sepsis Event) but were then retrospectively adjudicated to have an infection likelihood of none. Patients were assigned an infection likelihood of possible if they operationally had a Sepsis Event, but upon retrospective adjudication could not be classified with certainty as either a case or control. These patients were excluded from performance analyses but included in an analysis of factors leading to classification uncertainty.

8. Describe technical specifications of material and methods involved including how and when measurements were taken, and/or cite references for index tests and reference standard.

REFERENCE STANDARD

Discovery Cohort
Materials and Methods:

Final diagnosis of sepsis or infection-negative systemic inflammation was made by retrospective physician assessment using all available clinical and microbiological data and according to the ACCP/SCCM Consensus Statement for sepsis [23]. Consensus evaluations were made by two ICU physicians for the GCP-1 study and four ICU physicians for the RTT study, and were locked before microarray analysis of blood samples was initiated.

Validation Cohorts
Materials and Methods:

For the Sepsis Event nearest to ICU admission, a physician-assessed infection likelihood estimate of none, possible, probable, or definite was assigned retrospectively according to Center for Disease Control and Prevention and International Sepsis Forum consensus definitions [4, 24, 26]. See S2 Text for additional detail.

INDEX TESTS

Discovery Cohort
Materials and Methods:

For collection of microarray data from the Discovery Cohort, the data collection methods are described in detail in the text. We believe a detailed description of methods is necessary to allow reproduction in other laboratories.

Additionally, we present a detailed description of the techniques used to analyze the microarray data to identify the SeptiCyte Lab classifier. We employ relatively novel techniques for identify the classifier, which to our knowledge have not been previously published or referenced in the sepsis literature.

Validation Cohorts
Materials and Methods:

Data were acquired on multiple platforms as follows: Discovery Cohort on Affymetrix microarrays; Validation Cohort 1 on the Applied Biosystems (ABI) 7900HT Fast Real-Time PCR System, using TaqMan Low Density Array (TLDA) cards; Validation Cohorts 2, 3, 5 on the ABI 7500 Fast Real-Time PCR system using Life Technologies AmpliTaq RT-qPCR chemistry in strip tubes; Validation Cohort 4 on the ABI 7500 Fast Real-Time PCR system using Asuragen single-tube RT-qPCR chemistry in strip tubes. All PCR reactions were singleplex. S1 Data, entitled "Translation Between Microarray and RT-qPCR Formats", presents the primers, probes, dyes and quenchers for the four singleplex qPCR assays; the RT and qPCR buffers and thermal cycling programs; and also a series

of comparison tests from which linear shift formulae were derived for comparing and combining data across platforms. SeptiScores were adjusted to values that would be observed with the Asuragen RT-qPCR chemistry, using the linear shift formulae specified in S1 Data.

9. Describe definition of and rationale for the units, cutoffs and/or categories of the results of the index tests and the reference standard.

REFERENCE STANDARD

Discovery Cohort
Materials and Methods:

Final diagnosis of sepsis or infection-negative systemic inflammation was made by retrospective physician assessment using all available clinical and microbiological data and according to the ACCP/SCCM Consensus Statement for sepsis [23]. Consensus evaluations were made by two ICU physicians for the GCP-1 study and four ICU physicians for the RTT study….

Validation Cohorts
Materials and Methods:

Patients in each of the five Validation Cohorts were classified as either sepsis cases or infection-negative systemic inflammation controls. Patients were classified as sepsis cases if they experienced a Sepsis Event and were then adjudicated to have infection likelihood probable or definite for that event. Patients were classified as controls if: (1) they displayed two or more symptoms of systemic inflammation but were never given therapeutic systemic antibiotics (i.e. did not have a Sepsis Event); or (2) they displayed two or more symptoms of systemic inflammation and were given therapeutic systemic antibiotics (i.e. operationally defined to have had a Sepsis Event) but were then retrospectively adjudicated to have an infection likelihood of none. Patients were assigned an infection likelihood of possible if they operationally had a Sepsis Event, but upon retrospective adjudication could not be classified with certainty as either a case or control. These patients were excluded from performance analyses but included in an analysis of factors leading to classification uncertainty.

INDEX TESTS

Discovery Cohort
Materials and Methods:

We present a detailed description of the techniques used to analyze the microarray data to identify the SeptiCyte Lab classifier, in the text. We employ relatively novel techniques for identify the classifier, which to our knowledge have not been previously published or referenced in the sepsis literature.

Validation Cohorts

Materials and Methods:

A formula is presented in the text, which calculates the SeptiScore from a combination of observed Ct values of the individual measured RNA transcript levels….the output of SeptiCyte Lab in terms of RT-qPCR is a quantitative score: SeptiScore = $-C_{t,1} + C_{t,2} + -C_{t,3} + C_{t,4}$ where C_t = threshold cycle number and 1 = PLAC8, 2 = PLA2G7, 3 = LAMP1, 4 = CEACAM4 (Eq. 4).

(Results; Discussion):

A rationale is presented for considering different ranges of the SeptiScore, based on likelihood ratio calculations.

From the cumulative distributions of the SeptiScore for the sepsis and infection-negative systemic inflammation groups, we calculate likelihood ratios (LR) for different ranges of the SeptiScore, as indicated in Table 6.

Table 6. SeptiScores, likelihood ratios, and disease probabilities.[1]

**Score**	**N control**	**N sepsis**	**% of patients**	**% of controls**	**% of cases**	**Control:Case Ratio**	**LR**[2]	**Pre-test probability**[3]	**Post-test probability**
< 4	81	1	28.9	38.0	1.2	32:1	0.028	30%	1.2%
4 to 6	88	14	33.1	41.3	13.7	3:1	0.36	30%	16%
6 to 9	35	45	26.0	16.4	56.3	1:3.4	2.88	30%	55%
> 9	9	35	14.3	4.2	79.5	1:19	8.72	30%	79%

A test to discriminate sepsis from infection-negative systemic inflammation would need to lower the post-test probability of sepsis to a very low value (~1%) to be consistent with an experienced physician's decision to withhold antibiotics from a patient suspected of sepsis. Using the reported sepsis prevalence of ~30% in Dutch ICUs [60] and a SeptiScore less than 4, our calculations show a NPV of 98.8% which, if validated in a population-based cohort, may be sufficient for a clinician to withhold antibiotics, at least until follow-up diagnostic results are available.

10. Describe the number, training and expertise of the persons executing and reading the index tests and the reference standard.

Discovery Cohort
Materials and Methods:

Consensus evaluations were made by two ICU physicians for the GCP-1 study and four ICU physicians for the RTT study, and were locked before microarray analysis of blood samples was initiated.

Validation Cohorts
S2 Text:

To perform the evaluation, two trial physicians on site (at either the Amsterdam or Utrecht ICU) examined each patient's chart. (The two physicians were randomly selected from a pool of 8 physicians tasked with assessing patients.) A third trial physician was used to adjudicate in cases of discordance over the infection likelihood as per Klein-Klouwenberg et al. (2012).

11. Describe whether or not the readers of the index tests and reference standard were blind (masked) to the results of the other test and describe any other clinical information available to the readers.

Discovery Cohort
Materials and Methods:

Consensus evaluations were made by two ICU physicians for the GCP-1 study and four ICU physicians for the RTT study, and were locked before microarray analysis of blood samples was initiated.

Validation Cohorts
Materials and Methods:

All final classification of patients as either sepsis cases or controls was completed within three months of ICU admission, and locked before gene expression analysis of blood samples was initiated.

12. Describe methods for calculating or comparing measures of diagnostic accuracy, and the statistical methods used to quantify uncertainty (e.g., 95% confidence intervals).

Materials and Methods:

For all classifiers, accuracy of classifying cases vs. controls was evaluated by ROC curve analysis, with AUC used to quantify performance. AUCs and 95% confidence intervals were computed by resampling using the pROC package, version 1.5.4 [35], and were reported to two significant figures. Differences in AUC between pairs of ROC curves were evaluated for significance by Venkatramen's method [34] for microarray data from the Discovery Cohort, and by DeLong's test [36] for RT-qPCR data from the Validation Cohorts; p-values for the comparisons were calculated to two significant figures, with $p<0.05$ considered statistically significant. We did not use 2x2 contingency tables to assess performance to avoid loss of any diagnostic information contained in the full ROC curves [37-40].

13. Describe methods for calculating test reproducibility, if done.

Formal reproducibility studies were not conducted. However, estimates of assay variability can be obtained by analyzing the degree scatter of data about the lines of best fit, as measured by the correlation coefficient R^2, in the platform-to-platform comparisons described in S1 Data.

(Data were acquired on multiple platforms in the present study, and a series of platform-to-platform comparisons is presented in S1 Data. Data were acquired as follows: Discovery Cohort on Affymetrix microarrays; Validation Cohorts 1 on the Applied Biosystems (ABI) 7900HT Fast Real-Time PCR System, using TaqMan Low Density Array (TLDA) cards; Validation Cohorts 2, 3, 5 on the ABI 7500 Fast Real-Time PCR system using Life Technologies AmpliTaq RT-qPCR chemistry in strip tubes; Validation Cohort 4 on the ABI 7500 Fast Real-Time PCR system using Asuragen single-tube RT-qPCR chemistry in strip tubes. All PCR reactions were singleplex.)

14. Report when study was done, including beginning and ending dates of recruitment.

Discovery Cohort

Materials and Methods:

Patients were recruited sequentially at each study site, subject to the stated inclusion and exclusion criteria. Patients for the GCP-1 study were recruited from July 2008 to August 2009, and patients for the RTT study were recruited from May 2010 to December 2011. Final diagnosis of sepsis or infection-negative systemic inflammation was made by retrospective physician assessment using all available clinical and microbiological data and according to the ACCP/SCCM Consensus Statement for sepsis [23]. Consensus evaluations were made by two ICU physicians for the GCP-1 study and four ICU physicians for the RTT study, and were locked before microarray analysis of blood samples was initiated. Microarray analysis was conducted from November 2011 to April 2012 for the GCP-1 study, and from November 2011 to July 2012 for the RTT study.

Validation Cohorts

S2 Text

Validation Cohort 1: admitted to the Utrecht ICU from December 2012 to March 2013, but were not enrolled sequentially. RT-qPCR data for this cohort were generated in July 2013.

Validation Cohort 2: randomly picked from the Amsterdam ICU (n=19) or Utrecht ICU (n=17) over the entire available dates of the study (December 2012 to July 2013). RT-qPCR data for this cohort were generated in July and October 2013 (two batches).

Validation Cohort 3: drawn from an initial set of n=1004 patients consecutively admitted to the Amsterdam ICU (n=273) and Utrecht ICU (n=731) from December 2012 to July 2013. … After then applying the study exclusion criteria, the final numbers of patients from the two study centers were 52 patients from the Amsterdam ICU, and 54 patients from the Utrecht ICU. RT-qPCR data for this cohort were generated in November 2013.

Validation Cohort 4: drawn sequentially from the Amsterdam ICU from March 2013 to June 2013. RT-qPCR data for this cohort were generated in August 2014.

Validation Cohort 5: consecutively enrolled at the Amsterdam ICU (N=46) or Utrecht ICU (n=11) from November 2012 to August 2013. RT-qPCR data for this cohort were generated in April 2014.

15. Report clinical and demographic characteristics of the study population (e.g., age, sex, spectrum of presenting symptoms, comorbidity, current treatments, recruitment centers).

Discovery Cohort

Results:

The characteristics of the Discovery Cohort are described in Table 1. In addition, the following note is added: *Heterogeneity of Cases (Septic Patients):* Besides the specific exclusions listed in footnote #1 of Table 1, no patients were excluded based on predisposing condition, organ system affected, co-morbidities, presence of natural immunosuppression or immunosenescence, therapies, or type of pathogen detected. Patients with both systemic and local infections were included. A wide variety of pathogens were identified including Gram-positive bacteria, Gram-negative bacteria, fungi and mixed infections. Viral infections were generally not tested for.

Validation Cohorts

Results:

The selection process led to definition of five Validation Cohorts comprising 59, 36, 106, 87 and 57 patients respectively … which were chosen for different purposes and accordingly had different clinical and demographic characteristics (Table 2, S2 Text). The Validation Cohorts are briefly described as follows.

Validation Cohort 1 (n=59 consisting of 24 cases, 35 controls) contained only patients diagnosed with high confidence as having either sepsis or infection-negative systemic inflammation. This cohort consisted of patients admitted to the Utrecht ICU from December 2012 to March 2013, but not sequentially.

Validation Cohort 2 (n=36 consisting of 3 cases, 27 controls, 6 infection likelihood possible) contained patients that were randomly picked from the Amsterdam ICU (n=19) or Utrecht ICU (n=17) with ICU admission dates spanning the entire time frame of interest (December 2012 to July 2013). This cohort was used to test whether the score generated by SeptiCyte Lab exhibited any bias with respect to ICU admission date.

Validation Cohort 3 (n=106 consisting of 29 cases, 77 controls) was drawn from a consecutive sequence of 775 patients admitted to the Amsterdam ICU and Utrecht ICU from December 2012 to mid-July 2013. From this initial set of patients, 91 with an infection likelihood of possible were deliberately excluded. An additional four patients were excluded because insufficient data were captured to meet the minimum reporting requirements for retrospective physician adjudication. From the remaining pool (n=680), patients were randomly drawn to define this cohort (n=52 from Amsterdam and n=54 from Utrecht).

Validation Cohort 4 (n=87 consisting of 20 cases, 47 controls, 20 infection likelihood possible) consisted of patients who were consecutively admitted to the Amsterdam ICU from mid-March to mid-June, 2013. This cohort was used to assess performance in a real-world setting (i.e. sequential patients).

Validation Cohort 5 (n=57 consisting of 21 cases, 25 controls, 11 infection likelihood possible) contained exclusively patients of African or Asian ethnicity, who were consecutively admitted to the Amsterdam ICU (n=46) or Utrecht ICU (n=11) from December 2012 through July 2013. This cohort was used to determine if the performance of SeptiCyte Lab was affected by ethnicity in a real-world setting (i.e. sequential patients).

16. Report the number of participants satisfying the criteria for inclusion that did or did not undergo the index tests and/or the reference standard; describe why participants failed to receive either test (a flow diagram is strongly recommended).

<u>Discovery Cohort</u>
<u>Results:</u>

Footnotes # 1, 2 to Table 1 describe 39 patient exclusions that were made, starting from an initial set of 144 patients, to arrive at the final Discovery Cohort of 105 patients.

<u>Validation Cohorts</u>
<u>Materials and Methods:</u>

Patients were assigned an infection likelihood of possible if they operationally had a Sepsis Event, but upon retrospective adjudication could not be classified with certainty as either a case or control. These patients were excluded from performance analyses but included in an analysis of factors leading to classification uncertainty.

<u>Results:</u>

A total of 345 patients from the MARS study were selected for analysis and were grouped into five Validation Cohorts. Figure 1 presents a flow diagram indicating the inclusion and exclusion criteria used for patient selection. The Validation Cohorts comprising 59, 36, 106, 87 and 57 patients respectively were chosen for different purposes and accordingly had different clinical and demographic characteristics (Table 2,

S2 Text). A mapping of the patients in the Validation Cohorts back to the MARS study sites is given in S2 Text. Footnote #1 to Table 2 describes the exclusion from analysis of patients with an infection likelihood of possible.

17. Report time interval from the index tests to the reference standard, and any treatment administered between them.

Discovery Cohort
Materials and Methods:

Patients for the GCP-1 study were recruited from July 2008 to August 2009, and patients for the RTT study were recruited from May 2010 to December 2011. …Consensus evaluations were made by two ICU physicians for the GCP-1 study and four ICU physicians for the RTT study, and were locked before microarray analysis of blood samples was initiated. Microarray analysis was conducted from November 2011 to April 2012 for the GCP-1 study, and from November 2011 to July 2012 for the RTT study.

Validation Cohorts
Materials and Methods:

All final classification of patients as either sepsis cases or controls was completed within three months of ICU admission, and locked before gene expression analysis of blood samples was initiated. The precise dates of ICU admission and data generation for the Validation Cohorts (VC) were as follows. VC1: ICU admission December 2012 to March 2013; data generated July 2013. VC2: ICU admission December 2012 to March 2013; data generated July and October 2013 (two batches). VC3: ICU admission December 2012 to July 2013; data generated November 2013. VC4: ICU admission March 2013 to June 2013; data generated August 2014. VC5: ICU admission December 2012 to July 2013; data generated April 2014.

Note: All studies described in this work are *observational* only, and had no impact upon patient treatment or patient care.

18. Report distribution of severity of disease (define criteria) in those with the target condition; other diagnoses in participants without the target condition.

Discovery Cohort
Results:

Heterogeneity of Cases (Septic Patients): Besides the specific exclusions listed in footnote #1 of Table 1, no patients were excluded based on predisposing condition, organ system affected, co-morbidities, presence of natural immunosuppression or immunosenescence, therapies, or type of pathogen detected. Patients with both systemic and local infections were included. A wide variety of pathogens were identified including Gram-positive bacteria, Gram-negative bacteria, fungi and mixed infections. Viral infections were generally not tested for.

Validation Cohorts
Results:

Section 5 of Results examines disease severity as a potential confounding variable. Severity of disease could be a confounding variable in using SeptiCyte Lab to discriminate cases from controls. To address this concern a ROC curve analysis on the entire patient pool (n=308, excluding 37 patients with an infection likelihood of possible) was conducted using either the Sequential Organ Failure Assessment (SOFA) score or the Acute Physiology and Chronic Health Evaluation (APACHE) IV score as a classifier. …Additionally, each stratum of patients, characterized by a range of APACHE IV or SOFA scores, was analyzed to determine whether the performance of SeptiCyte Lab was stratum-dependent.

19. Report a cross tabulation of the results of the index tests (including indeterminate and missing results) by the results of the reference standard; for continuous results report the distribution of the test results by the results of the reference standard.

Discovery Cohort
Results:

The performance of gene expression ratios compared to the reference standard is described by ROC curves. From the Affymetrix core data set of >30,000 RefSeq-annotated transcripts, the top ranking ratio was *PLA2G7/PLAC8* with an AUC of 0.98. In an effort to further improve the AUC other high-ranking ratios were added to this top ratio. By adding *CEACAM4/LAMP1,* the AUC could be improved to 1.00. It must be stressed that neither the AUC of 0.98 nor the AUC of 1.00 obtained in this stage of analysis should be considered realistic estimates of the performance of this classifier on independent samples. These AUCs are merely the maximized values of the objective function used in the greedy search algorithm.

Highest discriminative power was obtained by combining the four RNA expression values into two ratios, and the addition of other RNA expression values or ratios did not significantly enhance performance. The combination of *PLA2G7/PLAC8* and *CEACAM4/LAMP1* RNA expression ratios, specified by Eq. (3), is referred to as the SeptiCyte Lab classifier.

The performance of individual RNA biomarkers comprising SeptiCyte Lab, in comparison to the reference standard is described by the heat map and scatter plots of Figure 2A, 2B.

Validation Cohorts
Results:

The performance of the *SeptiCyte® Lab* classifier compared to the reference standard is described by ROC curves in Figure 3 (Validation Cohort 1), Figure 4 (Validation Cohorts 3, 4, 5), and Table 4 (stratification by age, gender, ethnicity).

Table 4. Diagnostic performance of SeptiCyte Lab in the Validation Cohorts.

Validation Cohort	N analyzed	N excluded[1]	N controls	N cases	AUC[2] (95% CI)	Accuracy[4] (95% CI)	Sensitivity[4] (95% CI)	Specificity[4] (95% CI)	PPV[4] (95% CI)	NPV[4] (95% CI)	+LR[4] (95% CI)	-LR[4] (95% CI)
1	59	none	35	24	0.95 (0.91-1.00)	0.86 (0.75-0.94)	0.79 (0.58 –0.93)	0.91 (0.77-0.98)	0.86 (0.68-0.95)	0.86 (0.74-0.93)	9.24 (3.07-27.78)	0.23 (0.11-0.50)
2	30	6	27	3	0.77 (0.59-0.94)	0.70 (0.51-0.85)	1.00 (0.29-1.00)	0.67 (0.46-0.84)	0.17 (0.09-0.26)	0.97 (0.88-0.99)	3.00 (1.76-5.11)	0 (0-2.73)
3	106	none	77	29	0.93 (0.88-0.97)	0.76 (0.66-0.83)	0.97 (0.82-1.00)	0.675 (0.56-0.78)	0.53 (0.45-0.61)	0.98 (0.88-1.00)	2.97 (2.14-4.13)	0.051 (0.007-0.35)
4	67	20	49	18	0.85 (0.75-0.95)	0.51 (0.38-0.63)	1.00 (0.82-1.00)	0.33 (0.20-0.48)	0.27 (0.22-0.31)	0.95 (0.78-0.98)	1.48 (1.22-1.80)	0 (0-1.31)
5 (Black + Asian)	46	11	25	21	0.92 (0.85-1.00)	0.74 (0.59-0.86)	1.00 (0.84-1.00)	0.52 (0.31-0.72)	0.53 (0.43-0.62)	0.94 (0.74-0.98)	2.08 (1.38-3.13)	0 (0-0.71)
1+2+3+4	262	26	188	74	0.87 (0.82-0.91)	0.71 (0.65-0.76)	0.92 (0.83-0.97)	0.63 (0.55-0.70)	0.46 (0.41-0.51)	0.96 (0.91-0.98)	2.47 (2.03-3.01)	0.13 (0.060-0.28)
2+3+4+5	249	37	178	71	0.89 (0.85-0.93)	0.68 (0.62-0.74)	0.99 (0.92-1.00)	0.56 (0.48-0.63)	0.42 (0.38-0.46)	0.99 (0.94-1.00)	2.22 (1.88-2.62)	0.025 (0.004-0.18)
1+2+3+4+5	308	37	213	95	0.88 (0.84-0.92)	0.71 (0.66-0.76)	0.94 (0.87-0.98)	0.62 (0.55-0.68)	0.48 (0.44-0.52)	0.96 (0.92-0.98)	2.43 (2.04-2.91)	0.10 (0.047-0.24)
1+2+3+4+5, male only[3]	167	21	116	51	0.86 (0.81-0.92)	0.70 (0.62-0.77)	0.94 (0.84-0.99)	0.60 (0.50-0.68)	0.46 (0.41-0.52)	0.96 (0.90-0.99)	2.32 (1.84-2.93)	0.099 (0.032-0.30)
1+2+3+4+5, female only[3]	141	15	97	44	0.90 (0.84-0.95)	0.73 (0.65-0.80)	0.93 (0.81-0.99)	0.64 (0.54-0.73)	0.51 (0.44-0.57)	0.96 (0.89-0.99)	2.58 (1.96-3.41)	0.11 (0.035-0.32)
1+2+3+4+5,	182	20	125	57	0.89	0.72	0.95	0.62	0.49	0.97	2.47	0.085

<64 years					(0.84-0.94)	(0.65-0.78)	(0.85-0.99)	(0.54-0.70)	(0.44-0.55)	(0.91-0.99)	(1.96-3.11)	(0.028-0.26)
1+2+3+4+5, ≥64 years	126	17	88	38	0.87 (0.80-0.93)	0.71 (0.62-0.78)	0.92 (0.79-0.93)	0.61 (0.50-0.72)	0.46 (0.40-0.53)	0.96 (0.88-0.98)	2.38 (1.80-3.15)	0.13 (0.043-0.39)

[1] Patients with an infection likelihood of possible were excluded from performance analysis.
[2] Abbreviations used in this table: AUC, area under curve; CI, confidence interval; -LR, negative likelihood ratio; +LR, positive likelihood ratio; NPV, negative predictive value; PPV, positive predictive value.
[3] Sex was not recorded for one patient.
[4] For calculation of accuracy, sensitivity, specificity, PPV, NPV, +LR, -LR a binary cutoff of 3.100 for the SeptiScore was assumed.

In S6 Data, entitled "Analysis of Patients Classified as Infection Likelihood Possible", we investigate the behavior of SeptiCyte Lab in the patients classified as infection likelihood possible, who were excluded from performance analyses.

20. Report any adverse events from performing the index tests or the reference standard.

Not Applicable. The results of this investigation did not affect treatment decisions for any of the enrolled patients.

21. Report estimates of diagnostic accuracy and measures of statistical uncertainty (e.g., 95% confidence intervals).

<u>Discovery Phase</u>
<u>Results:</u>

From the Affymetrix core data set of >30,000 RefSeq-annotated transcripts, the top ranking ratio was *PLA2G7/PLAC8* with an AUC of 0.98. In an effort to further improve the AUC other high-ranking ratios were added to this top ratio. By adding *CEACAM4/LAMP1,* the AUC could be improved to 1.00. It must be stressed that neither the AUC of 0.98 nor the AUC of 1.00 obtained in this stage of analysis should be considered realistic estimates of the performance of this classifier on independent samples. These AUCs are merely the maximized values of the objective function used in the greedy search algorithm.

<u>Materials and Methods:</u>

AUC 95% confidence intervals were computed by resampling.

<u>Validation Phase</u>
<u>Results:</u>

The performance of the SeptiCyte Lab classifier compared to the reference standard is described by ROC curves in Figure 3 (Validation Cohort 1), Figure 4 (Validation Cohorts 3, 4, 5), and Table 3 (stratification by age, gender, ethnicity).

22. Report how indeterminate results, missing responses and outliers of the index tests were handled.

<u>Validation Phase</u>
<u>Results and S6 Data:</u>

To estimate the diagnostic performance of SeptiCyte Lab using ROC curve analysis, we first removed patients with an infection likelihood of possible. These are patients for which an assignment of infection likelihood, and therefore classification as either cases or controls could not be made with high confidence. However, by excluding these patients, spectrum bias may be introduced into estimates of performance [70-73]. To address this concern, a Kolmogorov-Smirnov test was used to determine whether the statistical

distribution of SeptiScores was different for patients with possible infection likelihood, as compared to patients with an unambiguous classification. The analysis was based on the entire available dataset (n=345) and compared all 37 cases of infection likelihood possible to the remaining 308 samples with known disease status. No significant difference was found (p = 0.37) between the cumulative distributions of the SeptiScore for these two classes of patients (see S6 Data). Thus, if only the SeptiScore is considered, patients with infection likelihood possible are indistinguishable from patients of known disease status.

23. Report estimates of variability of diagnostic accuracy between subgroups of participants, readers or centers, if done.

Validation Phase

Results:

In addition to testing SeptiCyte Lab performance on Validation Cohort 5 containing differing patient ethnicities, additional tests for the robustness of SeptiCyte Lab were performed by stratifying the entire dataset (n=308, with 37 cases of possible infection likelihood removed) on gender [64,65] or age (<64 and ≥64 years) [66-69]. By DeLong's test [36], the AUCs for these strata did not show significant differences (female vs. male: p = 0.52; age <64 years vs. age ≥64 years: p = 0.70). Thus, SeptiCyte Lab was able to differentiate sepsis from infection-negative systemic inflammation across both genders and a range of ages with high accuracy (AUC = 0.9).

Severity of disease could be a confounding variable in using SeptiCyte Lab to discriminate cases from controls. To address this concern a ROC curve analysis on the entire patient pool (n=308, excluding 37 patients with an infection likelihood of possible) was conducted using either the Sequential Organ Failure Assessment (SOFA) score or the Acute Physiology and Chronic Health Evaluation (APACHE) IV score as a classifier. This analysis, summarized in Figure 5, Panel A, revealed only a weak discrimination (AUC = 0.66 for APACHE IV; AUC = 0.52 for SOFA) in contrast to the strong discrimination (AUC = 0.88) achieved by SeptiCyte Lab.

Additionally, each stratum of patients, characterized by a range of APACHE IV or SOFA scores, was analyzed to determine whether the performance of SeptiCyte Lab was stratum-dependent. Comparison of ROC curves from individual strata revealed no significant differences (p > 0.23 for each APACHE IV comparison; p > 0.30 for each SOFA comparison). Results of this secondary analysis are summarized in Figure 5, Panel B, and presented in greater detail in S5 Data, entitled "Disease Severity as a Potential Confounding Variable". Thus, with respect to discrimination of sepsis and infection-negative systemic inflammation by SeptiCyte Lab, any confounding effect of disease severity, as measured by APACHE IV or SOFA score, appeared small.

24. Report estimates of test reproducibility, if done.

Formal reproducibility studies were not conducted. However, estimates of assay variability can be obtained by analyzing the degree scatter of data about the lines of best

fit, as measured by the correlation coefficient R^2, in the platform-to-platform comparisons described in S1 Data.

(Data were acquired on multiple platforms in the present study, and S1 Data presents a series of platform-to-platform comparisons. Data were acquired as follows: Discovery Cohort on Affymetrix microarrays; Validation Cohort 1 on the Applied Biosystems (ABI) 7900HT Fast Real-Time PCR System, using TaqMan Low Density Array (TLDA) cards; Validation Cohorts 2, 3, 5 on the ABI 7500 Fast Real-Time PCR system using Life Technologies AmpliTaq RT-qPCR chemistry in strip tubes; Validation Cohort 4 on the ABI 7500 Fast Real-Time PCR system using Asuragen single-tube RT-qPCR chemistry in strip tubes. All PCR reactions were singleplex.)

25. Discuss the clinical applicability of the study findings.

Discussion:

Clinical Utility: Early and accurate detection of sepsis, followed by appropriate therapeutic intervention, is critical for reducing patient morbidity and mortality. By the time sepsis reaches the advanced stage of septic shock, the nature of the problem is clear, but therapeutic intervention may be dangerously late. In the cohorts tested, we found that SeptiCyte Lab could distinguish cases from controls with a diagnostic accuracy approaching AUC 0.9, within several hours of the first suspicion of sepsis. When the test was run in binary mode with an appropriate cutoff, a high negative predictive value (95%) was obtained. In our cohorts, SeptiCyte Lab outperformed PCT (currently the only FDA-cleared protein biomarker of sepsis). The test retained high performance in patients showing few signs of organ dysfunction (i.e. low APACHE IV or SOFA scores), thus appeared unlinked to sepsis severity and able to diagnose sepsis early in the absence of multiple clinical signs.

Ultimately, the general clinical utility of SeptiCyte Lab will be evaluated through multiple validation studies in a variety of clinical settings, which will include patients with less definitive sepsis diagnoses [78]. If appropriately validated in further clinical cohorts, the information delivered by SeptiCyte Lab, in conjunction with available clinical parameters, may provide the physician with the ability to not only recognize sepsis in its early stages, but to also implement a targeted early treatment regime. It is expected that early, goal directed therapy will help prevent or minimize progression to multi-organ dysfunction. Low SeptiScores, which in our cohorts correlated with low sepsis probability, could also provide physicians with an objective basis for reducing or eliminating antibiotic treatment for patients who display 'sterile' systemic inflammation.

We have argued above that the accuracy, robustness and fast turnaround time of SeptiCyte Lab will have clinical utility for physicians attempting to rapidly diagnose sepsis and make appropriate therapeutic choices. However, because sepsis has a high risk of morbidity and mortality, attending ICU physicians will tend to prescribe antibiotics in cases for which there is simply a suspicion of sepsis [82]. A test to discriminate sepsis from infection-negative systemic inflammation would need to lower the post-test probability of sepsis to a very low value (~1%) to be consistent with an experienced physician's decision to withhold antibiotics from a patient suspected of sepsis. Using the

reported sepsis prevalence of ~30% in Dutch ICUs [60] and a SeptiScore less than 4, our calculations show a NPV of 98.8% which, if validated in a population-based cohort, may be sufficient for a clinician to withhold antibiotics, at least until follow-up diagnostic results are available.

Conclusions:

In combination with clinical parameters and clinical judgment, SeptiCyte Lab may provide physicians with enhanced confidence in therapeutic decision making for patients with systemic inflammation. Further clinical studies are required to confirm these findings.
